# MUC1-C dependency in drug resistant HR+/HER2− breast cancer identifies a new target for antibody-drug conjugate treatment

**DOI:** 10.1038/s41523-025-00751-w

**Published:** 2025-04-26

**Authors:** Ayako Nakashoji, Atrayee Bhattacharya, Hiroki Ozawa, Naoki Haratake, Keisuke Shigeta, Atsushi Fushimi, Nami Yamashita, Akira Matsui, Shoko Kure, Tomoe Kameyama, Makoto Takeuchi, Kazumasa Fukuda, Takamichi Yokoe, Aiko Nagayama, Tetsu Hayahsida, Yuko Kitagawa, Renyan Liu, Antonio Giordano, Rinath Jeselsohn, Geoffrey I. Shapiro, Donald Kufe

**Affiliations:** 1 Dana-Farber Cancer Institute, Harvard Medical School, Boston, MA USA; 2 Department of Breast Surgery, National Hospital Organization Tokyo Medical Center, Tokyo, Japan; 3 Department of Surgery, Keio University School of Medicine, Tokyo, Japan

**Keywords:** Breast cancer, Cancer therapy, Target identification

## Abstract

Treatment of hormone receptor (HR)-positive, HER2-negative breast cancer (HR+/HER2− BC) is limited by resistance to endocrine therapy (ET) and CDK4/6 inhibitors. There is no known common pathway that confers resistance to these agents. We report that (i) the *MUC1* gene is upregulated in HR+/HER2− BCs and (ii) the MUC1-C protein regulates estrogen receptor alpha (ER)-driven transcriptomes. Mechanistically, we demonstrate that MUC1-C is necessary for expression of SRC-3 and MED1 coactivators that drive ER-mediated target gene transcription. Cells with *ESR1* mutations that confer ET resistance, as well as cells with acquired resistance to the CDK4/6 inhibitor abemaciclib, are dependent on MUC1-C for (i) expression of these coactivators and ER target genes, (ii) survival, and (iii) self-renewal capacity. In support of these results, we show that treatment of HR+/HER2− BC cells with an anti-MUC1-C antibody-drug conjugate (ADC) effectively inhibits survival, self-renewal and tumorgenicity. These findings indicate that MUC1-C is a common effector of drug-resistant HR+/HER2− BC cells and is a potential target for their treatment.

## Introduction

Hormone receptor (HR)-positive, HER2-negative breast cancer (HR+/HER2− BC) accounts for 70% of BCs^1^. HR+/HER2− BC is conventionally treated with endocrine therapy (ET) comprised of aromatase inhibitors and selective ER modulators/degraders (SERMs/SERDs)^2–4^. ET reduces the risk of recurrence in the adjuvant setting and improves survival in patients with metastatic disease^2,3^. Nonetheless, resistance to ET is an inevitable outcome^2–4^. Mutations in the *ESR1* gene are a common mechanism for ET resistance, often occurring in the estrogen receptor alpha (ER) ligand binding domain at amino acids Y537 and D538^5–10^.

HR+/HER2− BCs are also treated with agents, such as palbociclib, abemaciclib and ribociclib, that target ER-mediated cyclin-dependent kinase 4 (CDK4)/CDK6 signaling^2,4,11^. Upregulation of the CDK4/6 pathway in HR+/HER2− BCs drives the G1/S phase transition by inactivating the retinoblastoma (RB) protein and inducing E2F transcription factor (TF) functions^12^. Combining agents that target ER signaling with CDK4/6 inhibitors has improved clinical outcomes^2,4,13,14^. However, HR+/HER2− BCs invariably develop resistance to CDK4/6 inhibitors by mechanisms that have been associated with loss of functional RB, increased CDK6 levels, activation of the cyclin E/CDK2 axis, and/or upregulation of receptor tyrosine kinase (RTK) and PI3K signaling^12^. Noteworthy is that no common pathway has been identified linking pleotropic resistance to both ET and CDK4/6 inhibitors, limiting potential therapeutic strategies to treat HR+/HER2- BCs unresponsive to these agents.

The *MUC1* gene was identified based on its overexpression in human breast cancers^15^. *MUC1* evolved in mammals to promote placentation and protection of barrier tissues^16^. *MUC1* encodes an oncogenic C-terminal (MUC1-C) subunit that is expressed at the apical membranes of polarized breast epithelial cells^16^. In response to the loss of homeostasis by biotic and abiotic insults, activation of MUC1-C contributes to wound repair by driving inflammatory, proliferative, and remodeling pathways^16^. These responses are theoretically reversible; however, in settings of chronic inflammation, prolonged activation of MUC1-C promotes cancer progression^16^. *MUC1* is overexpressed in HR+ BCs and is associated with poor clinical outcomes^17,18^. Direct interaction of ER and MUC1-C suppresses ER ubiquitylation and degradation by mechanisms that have remained unclear^19^.

The present studies demonstrate that MUC1-C is a common effector of HR+/HER2− BC cell resistance to agents targeting ER and CDK4/6 signaling. We report that MUC1-C confers resistance to these agents by regulating the ER coactivators (i) steroid receptor coactivator 3 (SRC-3/AIB1/NCOA3), and (ii) mediator subunit 1 (MED1). The identification of this common pathway of HR+/HER2− BC resistance lends support for MUC1-C as a target for the treatment of refractory disease, which has limited therapeutic options. Antibody-drug conjugates (ADCs) are approved for the treatment of metastatic BC, including HR+/HER2-low and HR+/HER2−^20–23^. In extending MUC1-C dependency of drug-resistant HR+/HER2− BCs, we demonstrate that an anti-MUC1-C ADC is effective against these recalcitrant cancers, uncovering a potential new approach for their treatment.

## Results

### *MUC1* is upregulated in HR+/HER2− BCs and regulates ER-driven gene transcriptomes

**Fig. 1:**
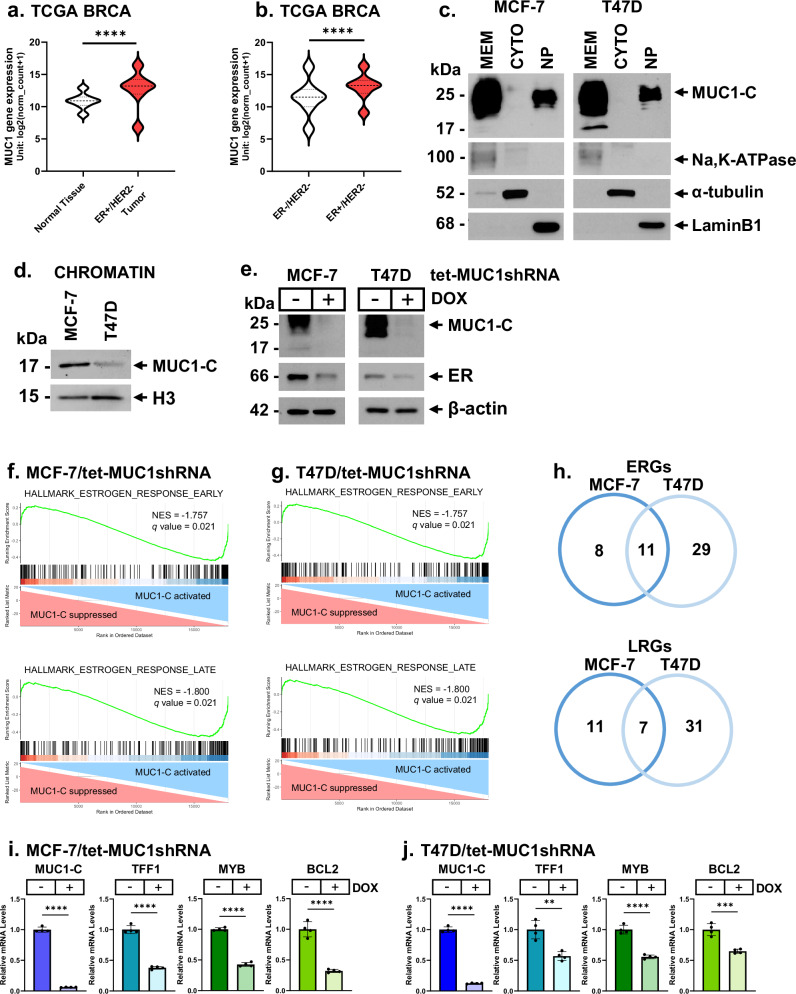
*MUC1* is upregulated in HR+/HER2− BCs and regulates ER-driven gene transcriptomes. **a** and **b** Analysis of the TCGA BRCA dataset for MUC1 expression in HR+/HER2− BCs vs normal breast tissue (**a**) and HR+/HER2− vs. ER−/HER2− BCs (**b**). **c** MCF-7 and T47D cell membrane (MEM), cytosolic (CYTO) and nucleoplasm (NP) were immunoblotted with antibodies against the indicated proteins. **d** Chromatin from MCF-7 and T47D cells was immunoblotted with antibodies against the indicated proteins. **e** Lysates from MCF-7/tet-MUC1shRNA and T47D/tet-shMUC1shRNA cells treated with vehicle or DOX for 7 days were immunoblotted with antibodies against the indicated proteins. **f** and **g**. GSEA of RNA-seq data from MCF-7 cells (**f)** and T47D cells (**g**) with MUC1-C silencing using the HALLMARK ESTROGEN RESPONSE EARLY (upper panels) and HALLMARK ESTROGEN RESPONSE LATE (lower panels) gene signatures. **h** Common downregulated ERGs and LRGs in MCF-7 and T47D cells with MUC1-C silencing. **i** and **j** MCF-7/tet-MUC1shRNA (**i**) and T47D/tet-MUC1shRNA (**j**) cells treated with vehicle or DOX for 7 days were analyzed for MUC1-C, TFF1, MYB and BCL2 transcripts by qRT-PCR using primers listed in Supplemental Table S2. The results (mean ± SD of 4 determinations) are expressed as relative levels compared to that obtained for vehicle-treated cells (assigned a value of 1).

Analysis of the TCGA BRCA dataset demonstrated that (i) *MUC1* is significantly upregulated in (i) ER+ vs. ER−, and (ii) HER2+ vs. HER2− BCs (Supplemental Fig. S1a). MUC1 expression in all HER2+ tumors (ER+ and ER− subtypes) vs. ER+/HER2− tumors was not significantly different; whereas, comparison of ER+/HER2− vs. ER−/HER2+ tumors was statistically significant (Supplemental Fig. S1a). We also found that *MUC1* significantly correlates with expression of *ESR1* and the ER target genes *TFF1* and *MYB* (Supplemental Fig. S1b). Further analysis identified upregulation of *MUC1* in (i) ER+/HER2− BCs vs. normal breast tissue (Fig. 1a), and (ii) ER+/HER2− vs. ER−/HER2− BCs (Fig. 1b). *MUC1* encodes an oncogenic MUC1-C protein comprised of 58 aa extracellular, 28 aa transmembrane and 72 aa cytoplasmic domains (Supplemental Fig. S1c). MUC1-C is expressed as ~25 kDa glycosylated and 17 kDa unglycosylated proteins (Supplemental Fig. S1c)^24^. The MUC1-C 25 kDa glycoprotein was detectable in the cell membrane and nucleoplasm of HR+/HER2− MCF-7 and T47D cells (Fig. 1c). Consistent with recent studies in NSCLC and HNSCC cells^25,26^, we found that the MUC1-C 17 kDa protein, but not the 25 kDa glycoprotein, localizes to chromatin in MCF-7 and T47D cells (Fig. 1d). In chromatin, the MUC1-C 17 kDa protein interacts with transcription factors (TFs) and epigenomic effectors to regulate gene expression^25,26^. In this way, MUC1-C forms an E2-dependent complex with ER^19^; however, the functional significance of this interaction, particularly in resistant HR+/HER2− BCs, has remained unclear^16^. Here, we found that inducible silencing of MUC1-C in MCF-7 and T47D, as well as ZR-75-1 HR+/HER2−, cells is associated with downregulation of ER expression (Fig. 1e; Supplemental Fig. S1d). ER suppression was also observed when targeting MUC1-C with (i) stable silencing using a second MUC1shRNA#2 (Supplemental Fig. S1e), and (ii) the GO-203 inhibitor, which blocks the MUC1-C CQC motif in the cytoplasmic domain that is necessary for nuclear import and function (Supplemental Fig. S1f)^16^. Based on these results, RNA-seq was performed on DOX-treated (i) MCF-7/tet-MUC1shRNA (Fig. 1f; Supplemental Fig. S1g) and (ii) T47D/tet-MUC1shRNA (Fig. 1g; Supplemental Fig. S1h) cells which demonstrated that MUC1-C silencing associates with downregulation of the HALLMARK ESTROGEN RESPONSE EARLY and HALLMARK ESTROGEN RESPONSE LATE, as well as the HALLMARK E2F TARGETS and HALLMARK G2M CHECKPOINT, gene signatures. Among the ER Early Response Genes (ERGs), 11 were commonly suppressed in MCF-7 and T47D cells (FDR < 0.05)(Fig. 1h; Supplemental Table S1). Additionally, 7 ER Late Response Genes (LRGs) were downregulated in both cell lines (Fig. 1h; Supplemental Table S1). Suppression of ER target genes *TFF1*^27^, *MYB*^28^ and *BCL2*^29^ located in the vicinity of super enhancers (SEs)^30^ was confirmed in MCF-7 and T47D cells with MUC1-C silencing (Fig. 1i, j). These results in HR+/HER2− BC cells indicate that MUC1-C is of importance in regulating the (i) ER early and late response and (ii) proliferative E2F and G2M signaling pathways.

### MUC1-C regulates the ER coactivator SRC-3

**Fig. 2:**
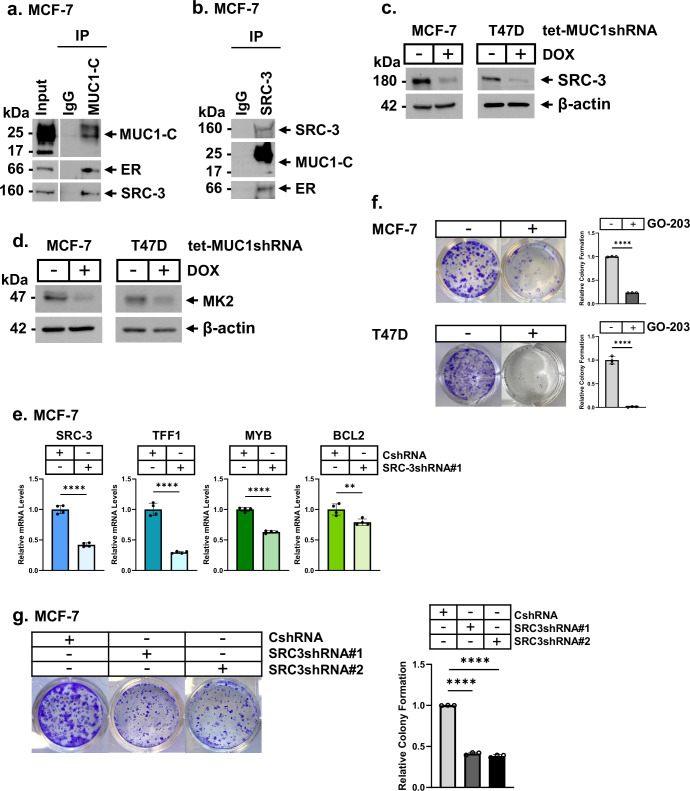
MUC1-C regulates ER coactivator SRC-3 by an MK2-mediated mechanism. **a** Nuclear lysates from MCF-7 cells treated with 100 nM 17β-estradiol (E2) for 3 h were precipitated with anti-MUC1-C or a control IgG. The precipitates and input lysate were immunoblotted with antibodies against the indicated proteins. **b** Nuclear lysates from MCF-7 cells treated with 100 nM E2 for 3 h were precipitated with anti-SRC-3 or a control IgG. The precipitates and input lysate were immunoblotted with antibodies against the indicated proteins. **c**, **d** Lysates of MCF-7/tet-MUC1shRNA and T47D/tet-MUC1shRNA cells treated with vehicle or DOX for 7 days were immunoblotted with antibodies against the indicated proteins. **e** MCF-7/CshRNA and MCF-7/SRC-3shRNA#1 cells were analyzed for the indicated transcripts by qRT-PCR. The results (mean±SD of 4 determinations) are expressed as relative levels compared to that obtained for CshRNA cells (assigned a value of 1). **f** MCF-7 and T47D cells treated with vehicle or 5 μM GO-203 were analyzed for colony formation. Shown are representative photomicrographs of stained colonies (left). The results (mean±SD of three determinations) are expressed as relative colony formation compared to that for vector cells (assigned a value of 1)(right). **g** MCF-7/CshRNA, MCF-7/SRC-3shRNA#1 and MCF-7/SRC-3shRNA#2 cells were analyzed for colony formation. Shown are representative photomicrographs of stained colonies (left). The results (mean±SD of three determinations) are expressed as relative colony formation compared to that for vector cells (assigned a value of 1)(right).

The SRC-3 ER coactivator is overexpressed in HR+ BCs and contributes to their progression^31–33^. To investigate if MUC1-C regulates ER target genes by an SRC-3-mediated mechanism, we first performed nuclear co-immunoprecipitation studies in MCF-7 cells, which demonstrated that MUC1-C forms complexes with ER and SRC-3 (Fig. 2a). These results were extended by analyzing anti-SRC-3 precipitates that detected nuclear complexes with ER and MUC1-C (Fig. 2b). Surprisingly, we found that silencing MUC1-C in MCF-7, T47D and ZR-75-1 cells downregulates SRC-3 expression (Fig. 2c; Supplemental Fig. S2a). Similar results demonstrating suppression of SRC-3 were obtained when targeting MUC1-C with (i) MUC1shRNA#2 (Supplemental Fig. S2b), and (ii) the GO-203 inhibitor (Supplemental Fig. S2c). Silencing MUC1-C in these cell models had modest effects on SRC-3 mRNA levels (Supplemental Fig. S2d), supporting potential regulation by a post-translational mechanism. SRC-3 is stabilized by mitogen-activated protein (MAP) kinase-activated protein kinase 2 (MAPKAPK2; MK2)-mediated phosphorylation at S857^34^. We found that (i) targeting MUC1-C with silencing and GO-203 treatment suppresses MK2 mRNA and protein levels (Fig. 2d; Supplemental Fig. S2e, f) and (ii) MUC1-C forms a complex with MK2 (Supplemental Fig. S2g). In addition, analysis of the TCGA dataset revealed that MUC1 significantly associates with MK2, but not SRC-3, expression in HR+/HER2- tumors (Supplemental Fig. S2h), consistent with MUC1-C-mediated regulation of SRC-3 by a post-transcriptional MK2-dependent mechanism. In further support for a MUC1-C → MK2 → SRC-3 pathway, targeting MK2 with the PF-3644022 inhibitor^35^ confirmed downregulation of SRC-3 expression (Supplemental Fig. S2i). By extension, silencing SRC-3 with two different SRC-3shRNAs decreased TFF1, MYB and BCL2 levels (Fig. 2e; Supplemental Fig. S2j). Furthermore, targeting MUC1-C (Fig. 2f), MK2 (Supplemental Fig. S2k) and SRC-3 (Fig. 2g) inhibited clonogenicity, in support of a MUC1-C → MK2 → SRC-3 pathway that regulates ER-driven function and survival.

### MUC1-C regulates CDK7 → MED1 signaling

**Fig. 3:**
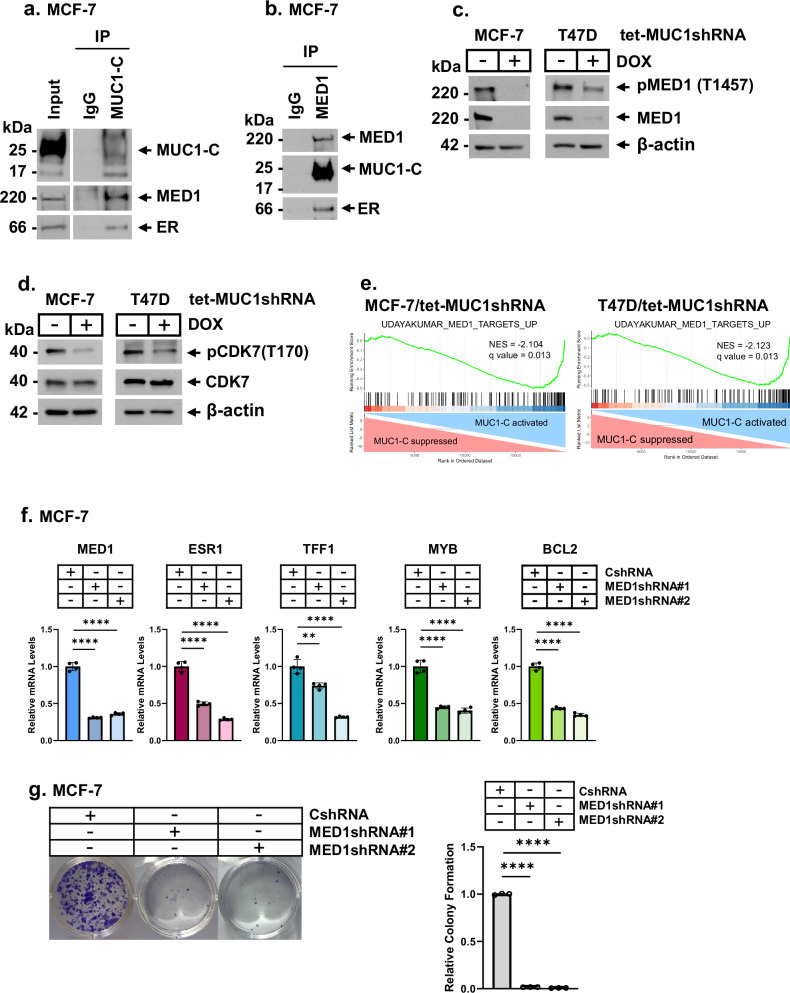
MUC1-C regulates ER coactivator MED1 by a CDK7-mediated mechanism. **a** Nuclear lysates from MCF-7 cells treated with 100 nM 17β-estradiol (E2) for 3 h were precipitated with anti-MUC1-C or a control IgG. The precipitates and input lysate were immunoblotted with antibodies against the indicated proteins. **b** Nuclear lysates from MCF-7 cells treated with 100 nM E2 for 3 h were precipitated with anti-MED1 or a control IgG. The precipitates and input lysate were immunoblotted with antibodies against the indicated proteins. **c**, **d** Lysates from MCF-7/tet-MUC1shRNA and T47D/tet-MUC1shRNA cells treated with vehicle or DOX for 5 days were immunoblotted with antibodies against the indicated proteins. **e** GSEA of RNA-seq data from MCF-7 and T47D cells with MUC1-C silencing using the UDAYAKUMAR MED1 TARGETS UP gene signature. **f** MCF-7/CshRNA, MCF-7/MED1shRNA#1 and MCF-7/MED1shRNA#2 cells were analyzed for MED1, ESR1, TFF1, MYB, and BCL2 transcripts by qRT-PCR. The results (mean ± SD of four determinations) are expressed as relative levels compared to that obtained for CshRNA cells (assigned a value of 1). **g** MCF-7/CshRNA, MCF-7/MED1shRNA#1 and MCF-7/MED1shRNA#2 cells were analyzed for colony formation. Shown are representative photomicrographs of stained colonies (left). The results (mean ± SD of three determinations) are expressed as relative colony formation compared to that for CshRNA cells (assigned a value of 1) (right).

ER has a LYDLL motif (aa 536–540) that functions as a binding site for SRC-3 and other coactivators^36^. Mediator subunit 1 (MED1) is an ER coactivator with an LXXLL motif that confers interactions between ER and Mediator^37,38^. As found for SRC-3, MUC1-C formed nuclear complexes with ER and MED1 (Fig. 3a, b). We also found that targeting MUC1-C in MCF-7, T47D and ZR-75-1 cells downregulates pMED1(T1457) and MED1 levels (Fig. 3c; Supplemental Fig. S3a, b) in the absence of a pronounced effect on MED1 transcripts (Supplemental Fig. S3c). MED1 is stabilized by CDK7-dependent phosphorylation at T1457^39,40^. Consistent with MED1 downregulation, targeting MUC1-C suppressed activation of CDK7, as evidenced by decreases in pCDK7(T170) and not CDK7 levels^41^ (Fig. 3d). We also found that MUC1-C associates with regulation of the UDAYAKUMAR MED1 UP and DN genes signatures in MCF-7 and T47D cells (Fig. 3e; Supplemental Fig. S3d). In concert with a MUC1-C → CDK7 → pMED1(T1457) pathway, targeting CDK7 with the samuraciclib inhibitor^40,42^ confirmed downregulation of pMED1(T1457) and MED1 expression and, as a control, pPol II(S7) levels (Supplemental Fig. S3e). In extending these studies, we silenced MED1 (Fig. 3f, left panel) and found (i) downregulation of ER, and ER target gene expression (Fig. 3f, right 4 panels) and (ii) inhibition of clonogenic survival (Fig. 3g), indicating that, in addition to SRC-3, MUC1-C regulates the CDK7 → MED1 pathway in HR+/HER2− BC cells.

### ER mutant cells are dependent on MUC1-C for ER signaling

**Fig. 4:**
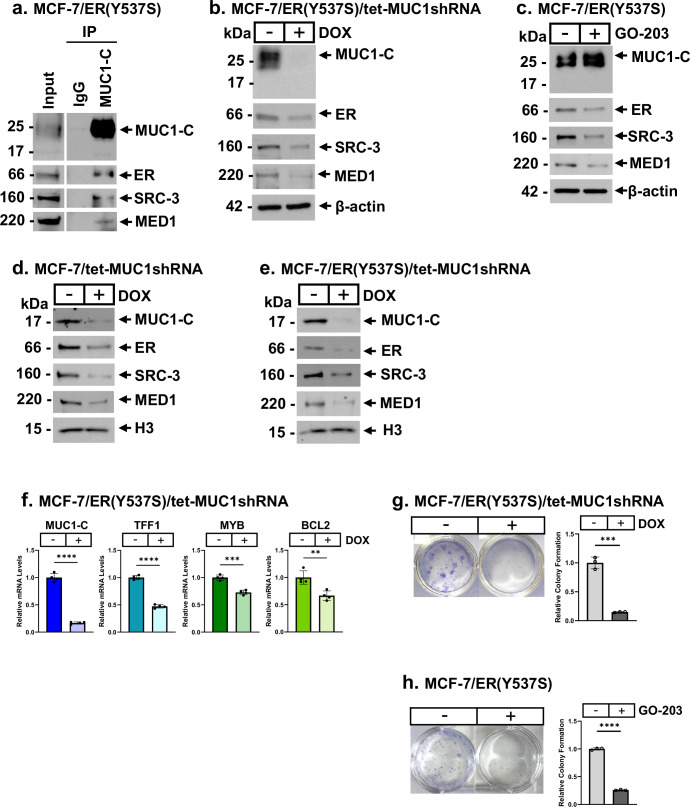
ER(Y537S) mutant cells are MUC1-C dependent. **a** Nuclear lysates from MCF-7(Y537S) cells grown in hormone deprived conditions for 2 days were precipitated with anti-MUC1-C or a control IgG. The precipitates and input lysate were immunoblotted with antibodies against the indicated proteins. **b** Lysates from MCF-7(Y537S)/tet-MUC1shRNA cells treated with vehicle or DOX for 7 days were immunoblotted with antibodies against the indicated proteins. **c** Lysates from MCF-7(Y537S) cells treated with vehicle or 5 μM GO-203 for 2 days were immunoblotted with antibodies against the indicated proteins. **d**, **e** Chromatin from MCF-7/tet-MUC1shRNA (**d**) and MCF-7/ER(Y537)/tet-MUC1shRNA (**e**) cells treated with vehicle or DOX for 11 days were immunoblotted with antibodies against the indicated proteins. **f** MCF-7(Y537S)/tet-MUC1shRNA cells treated with vehicle or DOX for 7 days were analyzed for MUC1-C, TFF1, MYB and BCL2 transcripts by qRT-PCR. The results (mean ± SD of four determinations) are expressed as relative levels compared to that obtained for vehicle-treated cells (assigned a value of 1). **g** MCF-7/ER(Y537S)/tet-MUC1shRNA cells treated with vehicle or DOX were analyzed for colony formation. Shown are representative photomicrographs of stained colonies (left). The results (mean±SD of three determinations) are expressed as relative colony formation compared to that for vehicle-treated cells (assigned a value of 1) (right). **h** MCF-7(Y537S) cells treated with vehicle or 5 μM GO-203 were analyzed for colony formation. Shown are representative photomicrographs of stained colonies (left). The results (mean ± SD of three determinations) are expressed as relative colony formation compared to that for vehicle-treated cells (assigned a value of 1)(right).

Binding of E2 to ER uncovers a surface for interactions with coactivators necessary for ER function^6,36^. Somatic ER mutations involving predominantly Y537S and D538G recruit coactivators in the absence of E2 and confer anti-estrogen resistance^6,36^. Having found that MUC1-C regulates SRC-3 and MED1, we asked if MUC1-C has an effect on ER(Y537S) signaling. Analysis of nuclear lysates from MCF-7/ER(Y537S) cells demonstrated that MUC1-C forms complexes with ER(Y537S), SRC-3 and MED1 in the absence and presence of E2 stimulation (Fig. 4a; Supplemental Fig. S4a). Targeting MUC1-C in MCF-7/ER(Y537S) cells genetically (Fig. 4b) and pharmacologically with GO-203 (Fig. 4c) decreased ER(Y537S), SRC-3 and MED1 expression. Silencing MUC1-C in MCF-7 cells also decreased MUC1-C expression in chromatin, as well as ER, SRC-3 and MED1 levels (Fig. 4d). Similar results were obtained in MCF-7/ER(Y537S) cells (Fig. 4e), indicating dependence on MUC1-C for localization of ER and these coactivators in chromatin. Consistent with these observations, MCF-7/ER(Y537S) cells grown under hormone deprived (HD) conditions exhibited dependence on MUC1-C for expression of ER target genes (Fig. 4f) and for clonogenic survival (Fig. 4g, h; Supplemental Fig. S4b). Mutation of the ER LYDLL motif to LYGLL (D538G) also confers constitutive ligand-independent activity and resistance to ET^6,36^. As found for the ER(Y537S) mutant, studies of MCF-7/ER(D538G) cells demonstrated that MUC1-C is necessary for regulation of ER target genes (Supplemental Fig. S4c) and survival (Supplemental Fig. S4d, e). These results indicate that ER(Y537S) and ER(D538G) mutant cells are dependent on MUC1-C for ER signaling.

### MUC1-C dependence in CDK4/6 inhibitor resistant HR+/HER2− BC cells

**Fig. 5:**
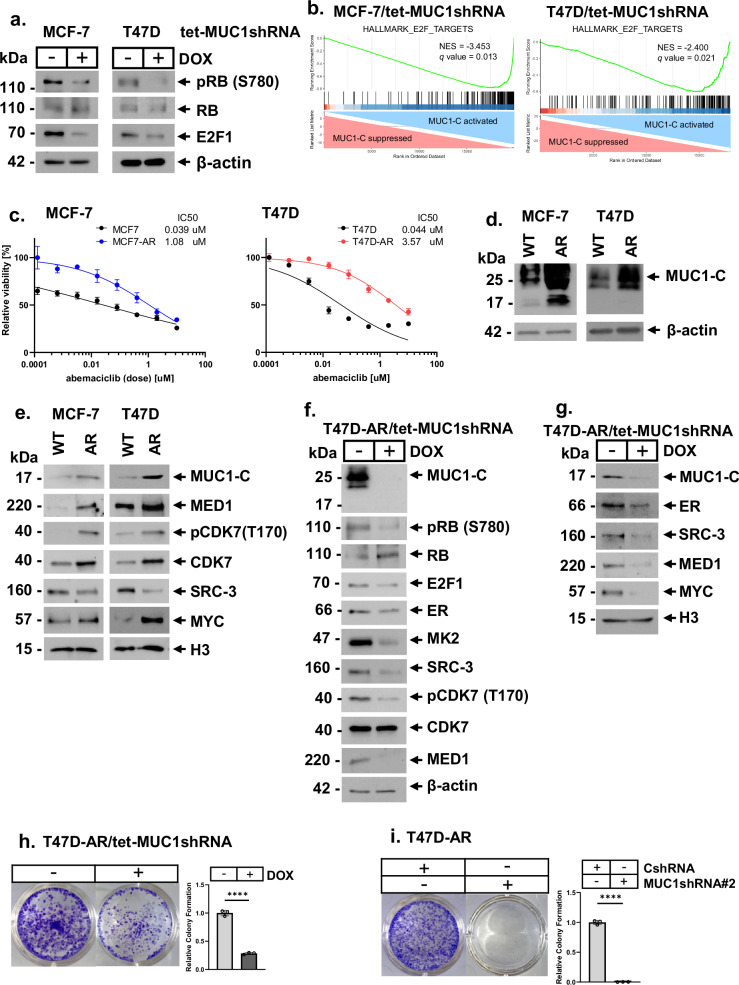
CDK4/6 inhibitor resistant HR+/HER2− BC cells are MUC1-C-dependent. **a** Lysates from MCF-7/tet-MUC1shRNA and T47D/tet-MUC1shRNA cells treated with vehicle of DOX for 7 days were immunoblotted with antibodies against the indicated proteins. **b** GSEA of RNA-seq data from MCF-7 cells and T47D cells with MUC1-C silencing using the HALLMARK E2F TARGETS gene signature. **c** Parental and abemaciclib-resistant MCF-7-AR and T47D-AR cells were treated with the indicated concentrations of abemaciclib for 6 days and analyzed for cell viability by Alamar Blue staining. The results (mean ± SD) are expressed as relative cell viability (% control) compared with that for untreated cells. **d** Lysates from MCF-7 and T47D parental wild-type (WT) and AR cells were immunoblotted with antibodies against the indicated proteins. **e** Chromatin from MCF-7 and T47D WT and AR cells was immunoblotted with antibodies against the indicated proteins. **f** Lysates from T47D-AR/tet-MUC1shRNA cells treated with vehicle of DOX for 7 days were immunoblotted with antibodies against the indicated proteins. **g** Chromatin from T47D-AR/tet-MUC1shRNA cells treated with vehicle or DOX for 14 days was immunoblotted with antibodies against the indicated proteins. **h** T47D-AR/tet-MUC1shRNA cells treated with vehicle or DOX were analyzed for colony formation. Shown are representative photomicrographs of stained colonies (left). The results (mean ± SD of three determinations) are expressed as relative colony formation compared to that for vehicle-treated cells (assigned a value of 1) (right). **i** T47D-AR/CshRNA and T47D-AR/MUC1shRNA#2 cells were analyzed for colony formation. Shown are representative photomicrographs of stained colonies (left). The results (mean ± SD of three determinations) are expressed as relative colony formation compared to that for CshRNA cells (assigned a value of 1) (right).

CDK4/6 inhibitors are integral to the treatment of HR+/HER2− breast cancers by targeting ER-mediated cyclin-dependent signaling. Combining ET with CDK4/6 inhibitors has improved clinical outcomes^2,4,13,14^; however, HR+/HER2− tumors invariably become resistant to these agents by unclear mechanisms. Therefore, to extend our studies on involvement of MUC1-C in resistance of HR+/HER2− BCs, we turned to the CDK4/6 pathway. ER-dependent activation of CDK4/6 complexes promotes RB inactivation by phosphorylation on S780 and in turn release of E2Fs to promote cell proliferation^43^. Acquired resistance to CDK4/6 inhibitors is associated with loss of functional RB^12^. In assessing regulation of RB signaling in parental MCF-7, T47D and ZR-75-1 cells, we found that targeting MUC1-C decreases pRB(S780) and E2F1 levels (Fig. 5a; Supplemental Fig. S5a), indicating that MUC1-C regulates the CDK4/6-RB/E2F axis. In accordance with these results, silencing MUC1-C in MCF-7 and T47D cells was associated with suppression of the HALLMARK E2F TARGETS (Fig. 5b), HALLMARK G2M CHECKPOINT and HALLMARK MITOTIC SPINDLE (Supplemental Fig. S5b, c) gene signatures. To determine if these effects of MUC1-C on the RB/E2F axis extend to settings of CDK4/6 treatment resistance, we established MCF-7 and T47D cells for growth in the presence of increasing abemaciclib concentrations of up to 500 nM (Fig. 5c). Abemacliclib-resistant MCF-7-AR and T47D-AR cells exhibited marked upregulation of MUC1-C expression in total lysates as compared to that in wild-type cells (Fig. 5d). We also found upregulation of MUC1-C in chromatin from MCF-7-AR and T47D-AR cells, which was associated with increases in pCDK7(T170), CDK7 and MED1, and by contrast suppression of SRC-3 levels (Fig. 5e). Additionally, MYC levels were increased in chromatin (Fig. 5e), which was of interest in that MYC contributes to CDK4/6 inhibitor resistance by regulating the RB/E2F axis^44^. Targeting MUC1-C genetically and with GO-203 treatment suppressed (i) pRB(S780) and E2F1, as well as (ii) ER, MK2, SRC-3, pCDK7(T170) and MED1 levels in total lysates (Fig. 5f; Supplemental Fig. S5d, e). MUC1-C was also necessary for expression of ER, SRC-3, MED1, and MYC in chromatin (Fig. 5g). Consistent with these results, silencing MUC1-C decreased TFF1, MYB, and BCL2 expression (Supplemental Fig. S5f). Furthermore, targeting MUC1-C in AR cells decreased survival (Fig. 5h, i; Supplemental Fig. S5g, h), indicating that HR+/HER2− AR cells are MUC1-C dependent.

### Targeting MUC1-C dependency in HR+/HER2− BC cells with an anti-MUC1-C ADC

**Fig. 6:**
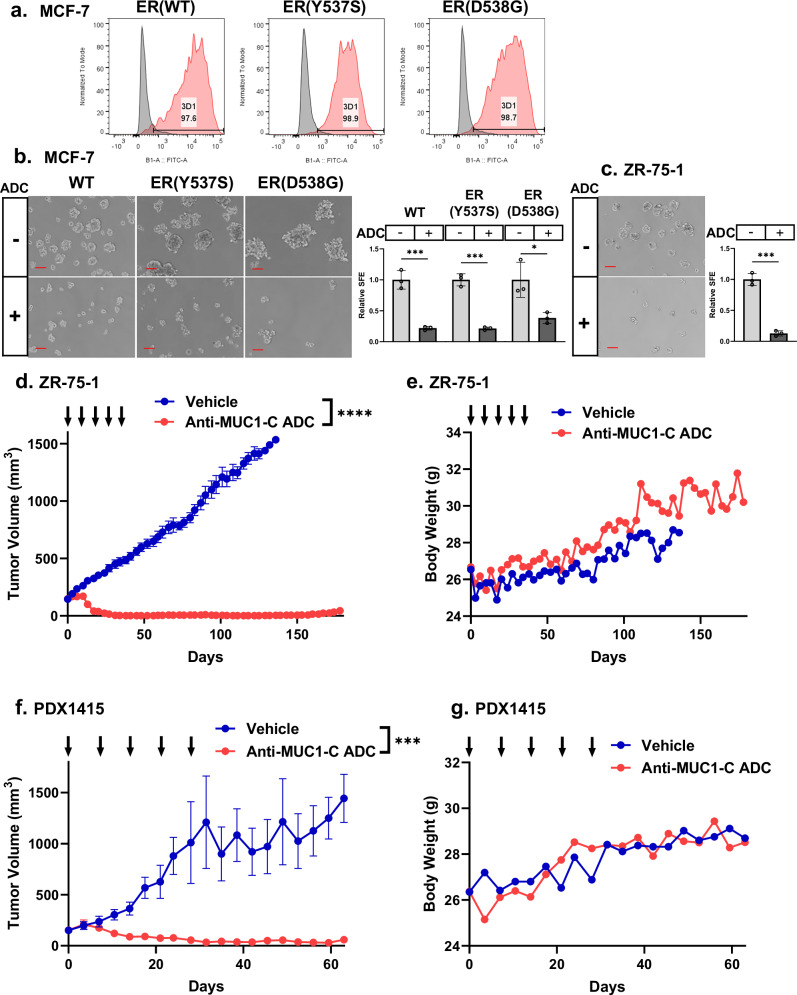
Anti-MUC1-C ADC is effective against treatment-resistant HR+/HER2− BC cells and tumors. **a** The designated MCF-7 cells were analyzed for cell surface MUC1-C expression by flow cytometry. **b**, **c** MCF7, MCF-7(Y537S) and MCF-7(D538G) (**b**) and ZR-75-1 (**c**) cells treated with 100 nM anti-MUC1-C ADC were analyzed for tumorsphere formation. Shown are representative photomicrographs of tumorspheres (left). Scale bar: 100 μm. The results (mean ± SD of three determinations) are expressed as relative sphere formation efficiency (SFE) compared to that for control cells (assigned a value of 1) (right). **d** Nude mice were injected with 1 × 10^7^ ZR-75-1 cells in the 4th mammary fat pad. Mice randomized into two groups were treated with vehicle or 5 mg/kg anti-MUC1-C ADC weekly for 5 weeks. Tumor volumes are expressed as the mean ± SEM. All 20 mice in the vehicle control group had progressive tumor growth with 10 sacrificed by day 78 and the remaining by day 144. The study was terminated at day 178 as tumor volumes remained static for multiple weeks. At that time, 6 of 10 mice in the ADC group were alive with only 2 bearing measurable tumors. Treatment with the ADC produced tumor regressions in all treated mice with all tumors being unmeasurable by day 31. *P* value was calculated on day 111. **e** Mean body weight changes of the ZR-75-1 tumor-bearing mice treated with vehicle or anti-MUC1-C ADC. The results are expressed as mean values for which the SEMs were <10% of the means. **f** PDX1415 tumor samples were implanted subcutaneously in ovariectomized NOD-SCID-IL2Rgc^−/−^ mice with E2 supplementation. Mice randomized into two groups when tumors reached 100 mm^3^ were treated with vehicle or 7.5 mg/kg anti-MUC1-C ADC weekly for 5 weeks. Tumor volumes are expressed as the mean ± SEM. *P* value was calculated on day 63. **g** Mean body weight changes of the PDX1415 tumor-bearing mice treated with vehicle or anti-MUC1-C ADC. The results are expressed as mean values for which the SEMs were <10% of the means.

Patients with HR+/HER− BCs (i) harboring ER mutations resistant to ET, and (ii) unresponsiveness to CDK4/6 inhibitors have few treatment options^2,4,45^. Having demonstrated that drug-resistant HR+/HER2− BC cells are MUC1-C dependent, we asked if MUC1-C is a potential target for their treatment. MUC1-C is expressed at lower levels on the apical borders of normal epithelia as compared to increased expression over the entire surface of depolarized breast and other types of cancer cells^15,16,46^. Given that there are no clinically effective agents against MUC1-C, we generated a monoclonal antibody (MAb), designated 3D1, against the MUC1-C ED alpha-3 helix (Supplemental Fig. S1c)^46^. Humanized huMAb 3D1 was conjugated using a maleimidocaproyl-valine-citrulline-*p*-aminobenzyloxycarbonyl (vc) cleavable linker to monomethyl auristatin E (MMAE) at a drug-antibody ratio (DAR) of ~4^46^. The anti-MUC1-C ADC was active against MUC1-positive, but not MUC1-negative, TNBC breast cancer cells and nontoxic in human MUC1-transgenic (MUC1.Tg) mice^46^. Here, flow cytometry studies demonstrated that MCF-7 cell surface expression of the anti-MUC1-C epitope is similar in wild-type and mutant ER(Y537S) and ER(D538G) cells (Fig. 6a)^46^. Sensitivity to the anti-MUC1-C ADC was also comparable in wild-type MCF-7 (IC_50_ = 6.3 nM), MCF-7/ER(Y537S) (IC_50_ = 27 nM), and MCF-7/ER(D538G) (IC_50_ = 1.8 nM) cells (Supplemental Fig. S6a). Analysis of MCF-7 vs. MCF-7-AR cells further demonstrated similar expression of the anti-MUC1-C epitope (Supplemental Fig. S6b) and sensitivity to the anti-MUC1-C ADC (Supplemental Fig. S6c). Moreover, T47D and T47D-AR cells exhibited comparable expression levels of the anti-MUC1-C epitope (Supplemental Fig. S6d) and responsiveness to the anti-MUC1-C ADC (Supplemental Fig. S6e). These results collectively indicated that BC cells resistant to ET and CDK4/6 inhibitors are targets for the anti-MUC1-C ADC. Eliminating the CSC population is arguably of importance for achieving long-term responses and potentially cures. We therefore evaluated effectiveness of the anti-MUC1-C ADC on self-renewal capacity as evidenced by tumorsphere formation^47^. In this way, we found that (i) MCF-7, MCF-7/ER(Y537S) and MCF-7/ER(D538G) (Fig. 6b), (ii) MCF-7-AR (Supplemental Fig. S6f), and (iii) T47D and T47D-AR (Supplemental Fig. S6g) CSCs are all sensitive to targeting with the anti-MUC1-C ADC, indicating that drug-resistant HR+/HER2− BC cells are potential candidates for treatment with this agent. To extend these results, we studied ZR-75-1 cells, which unlike MCF-7 and T47D cells, are null for PTEN, which confers resistance to CDK4/6 inhibitors in HR + BC cells^48^. As shown for MCF-7/AR and T47D/AR cells, we found that ZR-75-1 cells are sensitive to the anti-MUC1-C ADC as evidenced by loss of self-renewal capacity (Fig. 6c). We therefore treated established ZR-75-1 tumors in the 4th mammary fat pad with the anti-MUC1-C ADC and found complete and durable responses, which were significantly different as compared to control tumors (Fig. 6d). Moreover, as shown in MUC1.Tg mice and other mouse tumor xenograft models^46^, treatment with the anti-MUC1-C ADC was not associated with significant weight loss or other overt toxicities (Fig. 6e). To extend these results, we studied effects of the anti-MUC1-C ADC against the patient-derived xenograft ER-WT PDX1415 model derived from a patient with a HR+/HER2− BC refractory to treatment with aromatase inhibitor, fulvestrant, capecitabine, taxol, eribulin, and carboplatin-gemcitabine^49^. PDX1415 tumor samples were implanted subcutaneously in ovariectomized NOD-SCID-IL2Rgc^−/−^ mice with E2 supplementation. Treatment of established PDX1415 tumors with the anti-MUC1-C ADC resulted in suppression of growth that was significantly different vs. that in the control mice (Fig. 6f). As found for the ZR-75-1 tumor-bearing nude mice, the anti-MUC1-C ADC had no significant effect on body weight of NOD-SCID-IL2Rgc^−/−^ mice (Fig. 6g). These results demonstrate that treatment of HR+/HER2− BC cells with the anti-MUC1-C ADC inhibits self-renewal capacity and tumorigenicity in concert with targeting of the CSC population.

### Expression of MUC1-C in treatment-resistant HR+/HER2− BCs

**Fig. 7:**
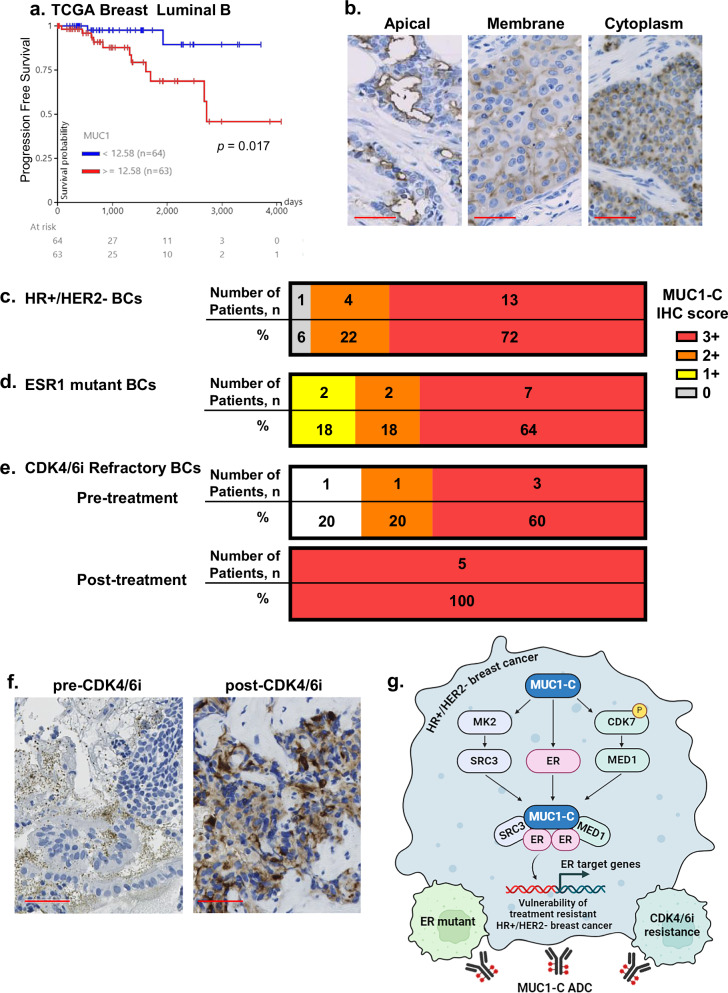
Expression of MUC1-C in HR+/HER2− BCs with ESR1 mutations and refractory to CDK4/6 inhibitor treatment. **a** Kaplan–Meier analysis of the TCGA BRCA Luminal B dataset for progression-free survival as a function of MUC1-high vs. MUC1-low tumors. **b** Representative examples of apical membranous, diffuse membranous and cytoplasmic staining for MUC1-C by IHC. Scale bar: 50 μm. **c–e** IHC score of MUC1-C levels on membranous, cytoplasmic or apical membranous regions were scored as 0, 1+, 2+ or 3+ based on the highest intensity occupying ≥10% of the evaluated area. The representative IHC score for HR+/HER2− (*n* = 18) (**c**), *ESR1* mutant (*n* = 11) (**d**) and CDK4/6 inhibitor resistant (*n* = 5) (**e**) BCs was determined by adopting the maximum score of each region. **f** IHC staining for MUC1-C expression of a HR+/HER2− BC metastatic to pleura pre-treatment (left) and post-treatment with ET in combination with CDK4/6 inhibition in a setting of progressive disease (right). Scale bar: 100 μm. **g** Schema depicting MUC1-C-mediated regulation of ER signaling in ER wild-type, ER mutant and CDK4/6 inhibitor resistant cells. Our results demonstrate that MUC1-C forms nuclear complexes with SRC-3 and MED1 and that targeting MUC1-C genetically and pharmacologically decreases SRC-3 and MED1 expression. Importantly, we further show that targeting MUC1-C suppresses ER, SRC-3 and MED1 levels in chromatin. We also demonstrate mechanistically that MUC1-C is necessary for expression of (i) MK2, which stabilizes SRC-3, and (ii) CDK7, which stabilizes MED1. These findings uncover the previously unrecognized role of MUC1-C in regulating SRC-3 and MED1 as effectors of drug resistance. Identification of this common pathway in drug-resistant HR+/HER2− BC cells formed the basis for the findings that an anti-MUC1-C ADC is effective for their treatment.

Given the findings that MUC1-C is a target for treating drug-resistant HR+/HER2− BCs, we asked if MUC1 expression is associated with clinical outcome in this patient population. Approximately half of the luminal B patients in the TCGA database lack treatment information. Among the remaining half with treatment data, ~90% (or ~45% of all patients with Luminal B BCs) received ER targeted therapy. Analysis of MUC1-high vs. MUC1-low expression in this treatment-resistant cohort demonstrated that MUC1 associates with a significant decrease in progression-free survival (PFS) (Fig. 7a). We therefore next analyzed 29 HR+/HER2− BCs for which the associated clinical characteristics and treatment information are detailed in Supplemental Tables S3a, b. MUC1-C was detectable in different patterns of apical, diffuse membrane and cytoplasmic expression (Fig. 7b). Assessment of HR+/HER2− BCs based on MUC1-C staining found that over 90% of tumors express 2+ and 3+ levels (Fig. 7c). Further analysis of HR+/HER2− BCs with pathogenic *ESR1* mutations identified expression of MUC1-C at 1+ to 3+ levels (Fig. 7d, Supplemental Table S3b). We also evaluated a cohort of patients with HR+/HER2− BCs refractory to ET in combination with palbociclib or abemaciclib (Supplemental Table S3c). Among these, 3 patients had MUC1-C 3+ positive tumors before and after treatment (Fig. 7e). In an additional two patients, MUC1-C levels were undetectable or 2+ in metastatic tumor tissue pre-treatment and were increased to 3+ post-treatment in the setting of progressive disease (Fig. 7e, f). These findings indicate that MUC1-C is expressed in a high proportion of treatment-resistant HR+/HER2− tumors.

## Discussion

HR+/HER2- BC is conventionally treated with ET and CDK4/6 inhibitors^2–4,11^. Combining ET with CDK4/6 inhibition has improved clinical outcomes^2,13,14^. Nonetheless, HR+/HER2- BCs invariably develop resistance to these therapies by pleotropic mechanisms that limit further treatment^12^. The present work uncovers MUC1-C as a common dependency of treatment-resistant HR+/HER2− BC cells. *MUC1* is overexpressed in HR+ BCs^17,18^; however, little is known about function of the oncogenic MUC1-C protein in the resistant disease setting. In addressing this limitation, we found in parental MCF-7 and T47D cells that MUC1-C is necessary for expression of ER early and late response genes. These results suggested that MUC1-C, which forms complexes with ER^19^, plays a role in the ER transactivation function (Fig. 7g). Our results demonstrate that MUC1-C forms complexes with ER that include the SRC-3 and MED1 coactivators. These coactivators are overexpressed in HR+ BCs and contribute to their progression^32,33,37,50^. SRC-3 and MED1 contain LXXLL motifs that interact with ER at LYDLL (aa 536–540) which is mutated in association with ET resistance^5–10^. We found that MUC1-C is necessary for SRC-3 and MED1 expression (Fig. 7g). Regarding SRC-3, silencing MUC1-C decreased expression of MK2, which stabilizes SRC-3 by phosphorylation at S857^34^. We also found that MUC1-C regulates activation of CDK7 that stabilizes MED1 by phosphorylation at T1457^39,40^. These results shed new light on previously unrecognized MUC1-C-dependent regulation of ER target gene expression by SRC-3- and MED1-mediated mechanisms (Fig. 7g).

Having identified these MUC1-C dependencies in drug-sensitive HR+/HER2− BC cells, we focused on MCF-7/ER(Y537S) mutant cells that exhibit constitutive ligand-independent activity and resistance to ET^6,36^. The ER(Y537S) mutant recruits SRC-3 in the absence of E2 stimulation by altering conformational dynamics of the ER LBD and stabilizing an agonist state^36^. In MCF-7/ER(Y537S) mutant cells, we found that MUC1-C (i) forms complexes with ER(Y537S) and SRC-3, and (ii) is necessary for SRC-3 expression. Similar results were obtained for MED1, indicating that, as found in parental cells, MUC1-C regulates SRC-3 and MED1 in the setting of the ER(Y537S) mutation (Fig. 7g). In support of this dependency, targeting MUC1-C in MCF-7/ER(Y537S) cells downregulated ER target gene expression and inhibited cell survival. To extend these studies, we generated abemaciclib-resistant MCF-7-AR and T47D-AR cells, which compared to their parental counterparts, exhibited upregulation of MUC1-C, SRC-3 and MED1 expression. Targeting MUC1-C in MCF-7-AR and T47D-AR cells downregulated (i) MK2 and SRC-3 and (ii) CDK7 and MED1, in support of dependency on MUC1-C expression. Resistance to CDK4/6 inhibitors has been attributed to increased CDK6 levels^12^. Unlike the SRC-3 and MED1 axes, MUC1-C had no apparent effect on CDK4 or CDK6. Nonetheless, targeting MUC1-C in the MCF-7-AR and T47D-AR cells suppressed ER target gene expression and clonogenic survival. These findings indicated that in the setting of CDK4/6 resistance, MUC1-C regulates the SRC-3 and MED1 coactivators and thereby the ER transactivation function necessary for survival (Fig. 7g). The SRC-3 and MED1 coactivators can contribute to ER-mediated gene transcription by additional mechanisms including liquid-liquid phase separation and the formation of condensates on enhancers and super enhancers (SEs)^51–53^. Among these*, ESR1* and ER target genes are driven by SEs in MCF-7 cells^30,54^. Noteworthy in this regard is that MUC1-C (i) has an intrinsically disordered cytoplasmic domain and other characteristics that contribute to condensates, and (ii) is necessary for the formation of paraspeckles and other membrane-less condensates^55^.

Patients with HR+/HER2− BCs with acquired resistance to ET and CDK4/6 inhibitors have limited therapeutic options. The present studies demonstrate that HR+/HER2− BC cells resistant to these agents are dependent on MUC1-C signaling as a common mechanism for survival (Fig. 7g). MUC1-C drives the CSC state in pan-cancers, including TNBC; whereas, less is known about such a role in HR /HER2− BCs^16,56^. Treatment of MCF-7 and MCF-7/ER(Y537S) cells with the anti-MUC1-C ADC was effective in inhibiting self-renewal capacity, indicating that these HR+/HER2− BC CSCs are MUC1-C-dependent. The ER(D538G) mutation is, like ER(Y537S), prevalent in HR+/HER2− BCs that are resistant to ET^5–10^. We found that treatment of MCF-7/ER(D538G) cells with the anti-MUC1-C ADC also suppresses self-renewal capacity, indicating that this agent is a potential candidate for patients with HR+/HER2− BC harboring the highly prevalent ER(Y537S) and ER(D538G) mutations. Consistent with MUC1-C dependency of MCF-7-AR and T47D-AR cells for ER signaling, treatment of these models with the anti-MUC1-C ADC demonstrated loss of self-renewal capacity, indicating that MUC1-C is necessary for the CSC state across HR+/HER2− BCs with pleotropic mechanisms of acquired treatment resistance (Fig. 7g). Furthermore, the anti-MUC1-C ADC was effective in suppressing growth of HR+/HER2− BC cell line and PDX tumor xenografts. Of importance clinically, we found that MUC1-C is widely expressed in HR+/HER2− BCs that harbor ESR1 mutations, as well as those refractory to CDK4/6 inhibitors. We also found that MUC1-C expression in HR+/HER2− BCs is heterogenous, which could be a factor for effectiveness of the anti-MUC1-C ADC. Subsequent studies will therefore be needed to assess the potential for bystander effects of anti-MUC1-C ADC treatment. Based on these findings, the anti-MUC1-C MIC ADC is under development by the NCI NExT Program in support of IND-enabling studies for the treatment of patients with HR+/HER2− BCs refractory to ET and CDK4/6 inhibitors.

## Methods

### Cell culture

MCF-7 and T47D cells (ATCC) were cultured in MEM (#10-010-CV, Corning, Corning, NY, USA) and RPMI medium (#11875-119, Thermo Fisher Scientific, Waltham, MA, USA), respectively, supplemented with 10% fetal bovine serum (FBS; #100-106, GEMINI Bio-Products, West Sacramento, CA, USA) and 10 μg/ml human recombinant insulin (#12585-014, Thermo Fisher Scientific). ZR-75-1 cells (ATCC) were cultured in RPMI medium with 10% FBS. MCF-7 cells with TALEN knock-in Y537S and D538G ER mutations were cultured as described^49^. For hormone deprived conditions, cells were cultured in phenol-red-free DMEM (#17-205-CV, Corning) supplemented with 10% charcoal stripped FBS (#F6765, Sigma-Aldrich, Burlington, MA, USA). Cells harboring knock-in ER mutations were cultured under HD conditions for a minimum of two days prior to sample collection, with the media refreshed daily. Cells were treated with abemaciclib (cat# S7158; Selleck Chemicals, Houston, TX, USA), samuraciclib (ICEC09402, cat# S8722; Selleck Chemicals), PF-3644022 (cat# PZ0188, Sigma), β-Estradiol (cat# E2758-250MG, Sigma) and GO-203 (cat# S8674, Selleck Chemicals). Authentication of the cells was performed every 3–4 months by short tandem repeat (STR) analysis. Cells were monitored for mycoplasma contamination every 3-4 months using the MycoAlert Mycoplasma Detection Kit (#LT07-218, Lonza, Rockland, MA, USA). Cells were maintained for 3-4 months when performing experiments.

### Gene silencing

MUC1shRNA (MISSION shRNA TRCN0000122938; Sigma, St. Louis, MO, USA) or a control scrambled shRNA (CshRNA; Sigma) was inserted into the pLKO-tet-puro vector (Plasmid #21915; Addgene, Cambridge, MA, USA) as described^25^. The MUC1shRNA#2 (MISSION shRNA TRCN0000430218) SRC-3shRNA#1 (TRCNTRCN0000365196), SRC-3shRNA#2 (TRCN0000370321), MED1shRNA#1 (TRCN0000019800), and MED1shRNA#2 (TRCN0000019800) were produced in HEK293T cells as described^25^. Vector-transduced cells were selected for growth in 1–2 μg/ml puromycin. Cells were treated with 0.1% DMSO as the vehicle control or 500 ng/ml doxycycline (DOX; Millipore Sigma).

### Quantitative reverse-transcription PCR (qRT-PCR)

Total cellular RNA was isolated using Trizol reagent (#15596018, Thermo Fisher Scientific). cDNAs were synthesized using the High Capacity cDNA Reverse Transcription Kit (#4387406, Thermo Fisher Scientific) as described^25^. The cDNA samples were amplified using the Power SYBR Green PCR Master Mix (#4367659, Thermo Fisher Scientific) and the CFX96 Real-Time PCR System (BIO-RAD, Hercules, CA, USA) as described^25^. Primers used for qRT-PCR are listed in Supplementary Table S2.

### Immunoblot analysis

Total lysates and chromatin-bound protein prepared from non-confluent cells were subjected to immunoblot analysis using anti-MUC1-C (#MA5–11202, 1:1000 dilution; Thermo Fisher Scientific, Waltham, MA, USA), anti-NaKATPase1 (3010S, 1:1000, Cell Signaling Technology, Danvers, MA, USA), anti-LaminB1 (66095-1-Ig, 1:2000, Proteintech, Rosemont, IL, USA), anti-α-tubulin (2144S, 1:1000, CST), anti-H3 (9715S, 1:1000, CST), anti-ER (8644S, 1:1000, Cell Signaling Technology), anti-β-actin (A5441, 1:10,000 dilution, Sigma-Aldrich), anti-SRC-3 (2126S, 1:1000, CST), anti-pSRC3(S857) (PA5-106189, Thermo Fischer Scientific), anti-MK2 (12155S, 1:1000, CST), anti-MED1 (51613S, 1:1000, CST), anti-pMED1(T1457) (ab60950, 1:500, abcam), anti-CDK7 (2916S, 1:1000, CST), anti—pCDK7(T170) (ab155976, 1:1000, abcam), anti-pPol II(pRpb1)(S7) (13780S, 1:500, CST), anti-Pol II(Rpb1) (14958T, 1:1000, CST), anti-pRB(S780) (9307S, 1:1000, CST), anti-RB (9309S, 1:1000, CST) and anti-E2F1 (3742, 1:1000, CST) as described^25^.

### Co-immunoprecipitation studies

Nuclear lysates were isolated as described^57^. Nuclear proteins were incubated with anti-MUC1-C (#MA5–11202; Thermo Fisher Scientific), anti-SRC-3 (2126S, CST), anti-MED1 (51613S, CST), Armenian Hamster IgG (ab18479, abcam) or Rabbit IgG (#NI01-100UG, Sigma), precipitated with Dynabeads Protein G (#10004D; Thermo Fisher Scientific) and analyzed as described^57^.

### Colony formation assays

Cells were seeded in 24-well plates for 24 h and then treated with (i) 0.1% DMSO or 500 ng/ml DOX, and (ii) PBS or GO-203. For cells harboring knock-in ER mutations were continuously maintained under HD conditions. After 7–14 days, cells were fixed with methanol and stained with 1% crystal violet (#LC135417, LabChem, Zelienople, PA, USA). Growth was quantified at 590 nm using a spectrophotometer and normalized to vehicle treatment.

### Cell proliferation and drug sensitivity assays

Cells were seeded at a density of 1500–3500 cells per well in 96-well plates. The next day, the cells were treated with different concentrations of the drug. Cell viability and proliferation were assessed using the Alamar Blue Reagent (cat# DAL1100, Thermo Fisher Scientific) following the company protocol. The IC50 values were determined by nonlinear regression of the dose–response data using Prism 10.0 (SCR_002798, GraphPad Software). Fluorescence intensity (560 nm excitation/590 nm emission) was measured in at least triplicate wells.

### Subcellular protein extraction

Subcellular Protein Fractionation Kit (cat# 78840, Thermo Fisher Scientific) was used to isolate subcellular proteins.

### Flow cytometry

Cells (1 × 10^6^) were blocked by incubation with 1% BSA/PBS for 20 min on ice. Cells were then incubated with 40 μg/ml mAb 3D1 or 40 μg/ml IgG1 kappa isotype control antibody (cat# 60070.1; STEMCELL Technologies, Vancouver, BC, Canada) for 1 h on ice. Goat Anti-Mouse IgG (Alexa Fluor 488) (ab150113, 1:100 dilution, Abcam) was used as the secondary reagent. Antibody binding to the cell surface was measured by MACSQuant Analyzer 10 Flow Cytometer (Miltenyi Biotec, Bergisch Gladbach, NRW, Germany) and analyzed by FlowJo v10.6.2 (BD Biosciences, Franklin Lakes, NJ, USA) software.

### Tumorsphere formation assays

Cells (5 × 10^3^) were seeded per well in 6-well ultra-low attachment culture plates (cat# 3471, Corning Life Sciences) in DMEM/F12 50/50 medium (cat# 10565-018, Corning Life Sciences) with 20 ng/ml EGF (cat# E9644, Millipore Sigma), 20 ng/ml bFGF (#100-18B, Thermo Fisher Scientific), and 2% B27 supplement (#17504044, Thermo Fisher Scientific). Cells were treated with PBS or anti-MUC1-C ADC. After 7 days, tumorspheres over 100 μm were counted under an inverted microscope in triplicate wells.

### RNA sequence analysis

Total RNA from cells cultured in triplicates was isolated using RNeasy Plus Mini Kit (#74134, QIAGEN, Hilden, Germany) as described^26^. TruSeq Stranded mRNA (Illumina, San Diego, CA, USA) was used for library preparation. Raw sequencing reads were aligned to the human genome (GRCh38) using STAR. Raw feature counts were normalized and differential expression analysis using DESeq2 as described^58^. Differential expression rank order for subsequent GSEA was performed using the packages tidyverse (v2.0.0), dbplyr (v2.4.0), and enrichplot (v1.22.0) in R (v4.3.2). Hallmark Gene Sets were queried through the Molecular Signatures Database.

### HR+/HER2− ZR-75-1 and PDX1415 tumor xenograft studies

ZR-75-1 cells were suspended in 50% RPMI-1640:50% Matrigel® at 1 × 10^8^/ml. Athymic nude (nu/nu NCr bred in-house) 7-week old female mice were implanted subcutaneously (SC) with an 0.18 mg estradiol pellet (Innovative Research, FL, USA). After 24 h, the mice were implanted with 100 μl of ZR-75-1 cells in the 4th mammary fat pad. Tumors were staged to 150 mm^3^ (range 80–195 mm^3^) and mice were randomized into groups using StudyLog®. Treatment was performed using once weekly × 5 intravenous (IV) dosing of (1) 10% DMSO in 90% D5W vehicle control (*n* = 20) and (2) 5 mg/kg huMAb 3D1-MMAE ADC (*n* = 10). Tumor volumes were collected as bidirectional caliper measurements using StudyDirector® (StudyLog, San Franscisco, CA). Individual body weights were recorded at each tumor volume assessment. The estradiol pellet was replaced in all surviving mice at day 103 to ensure adequate maintenance of estradiol. Mice were not dosed if they had body weight loss of 15% or greater. Mice were humanely sacrificed when their tumor reached approximately 1500 mm^3^ in volume or if clinical signs suggested estrogen toxicity. For patient-derived xenograft (PDX) studies, patient consent for tumor tissue implantation in nude mice was obtained under an Institutional Review Board (IRB)-approved protocol (Dana-Farber/Harvard Cancer Center IRB protocol 93-085). Tumor samples from PDX1415 were dipped in 50% matrigel and implanted subcutaneously in ovariectomized NOD-SCID-IL2Rgc^–/–^ mice (Jackson Laboratory, ME, USA), supplemented with 0.18 mg E2 pellets. When tumors reached 150–200 mm^3^, mice were randomized into two arms of once weekly x 5 intravenous (IV) dosing of (1) vehicle control (*n* = 6) and (2) 7.5 mg/kg anti-MUC1-C ADC (*n* = 6).

### Immunohistochemistry (IHC)

HR+/HER2− breast cancer specimens from patients who underwent surgery or biopsy at the Department of Surgery, Keio University School of Medicine were subjected to IHC with an anti-MUC1-C rabbit monoclonal antibody (16564, 1:1000 dilution; CST, heat-induced epitope retrieval, pH 6.0) as previously described^59^. The determinations were performed independently by two investigators (AN and SK). IHC score of MUC1-C on membranous, cytoplasmic or apical membranous region were scored as 0, 1+, 2+ or 3+ based on the highest intensity occupying ≥10% of the evaluated area. The representative IHC score for each specimen was determined by adopting the maximum score of each region. The apical membrane of mammary duct epithelium was used as internal controls for MUC1-C staining. The ethics committee of Keio University School of Medicine approved this study under the approval number 20190057. Informed consent or a suitable substitute in accordance with the Declaration of Helsinki was obtained from the patients in the study.

### Detection of *ESR1* mutations in HR+/HER2− BCs

DNA extraction from patient tumor samples, target amplification and sequencing were performed as described^60^ under the protocol 20190057 approved by the ethics committee of Keio University School of Medicine.

### Statistical analysis

Each experiment was performed at least three times. Unpaired two-tailed Student’s *t*-tests were used to assess differences between the mean±SD of two groups. *P*-values were considered significant at *p* < 0.05. GraphPad Prism9 was used for all statistical analyses. Asterisks represent *P ≤ 0.05, **P ≤ 0.01, ***P ≤ 0.001, ****P ≤ 0.0001 with CI = 95%.

## Supplementary information

Supplementary Figures 1–8, Supplementary Tables 1–3

## Data Availability

The accession number for the RNA-seq data is GEO Submission GSE272989.
