# Supplementary material for: MUC1-C dependency in drug resistant HR+/HER2− breast cancer identifies a new target for antibody-drug conjugate treatment
Source: NPJ Breast Cancer. 2025 Apr 26;11:39. doi: 10.1038/s41523-025-00751-w (PMC12033257; doi:10.1038/s41523-025-00751-w)

Supplemental Figures

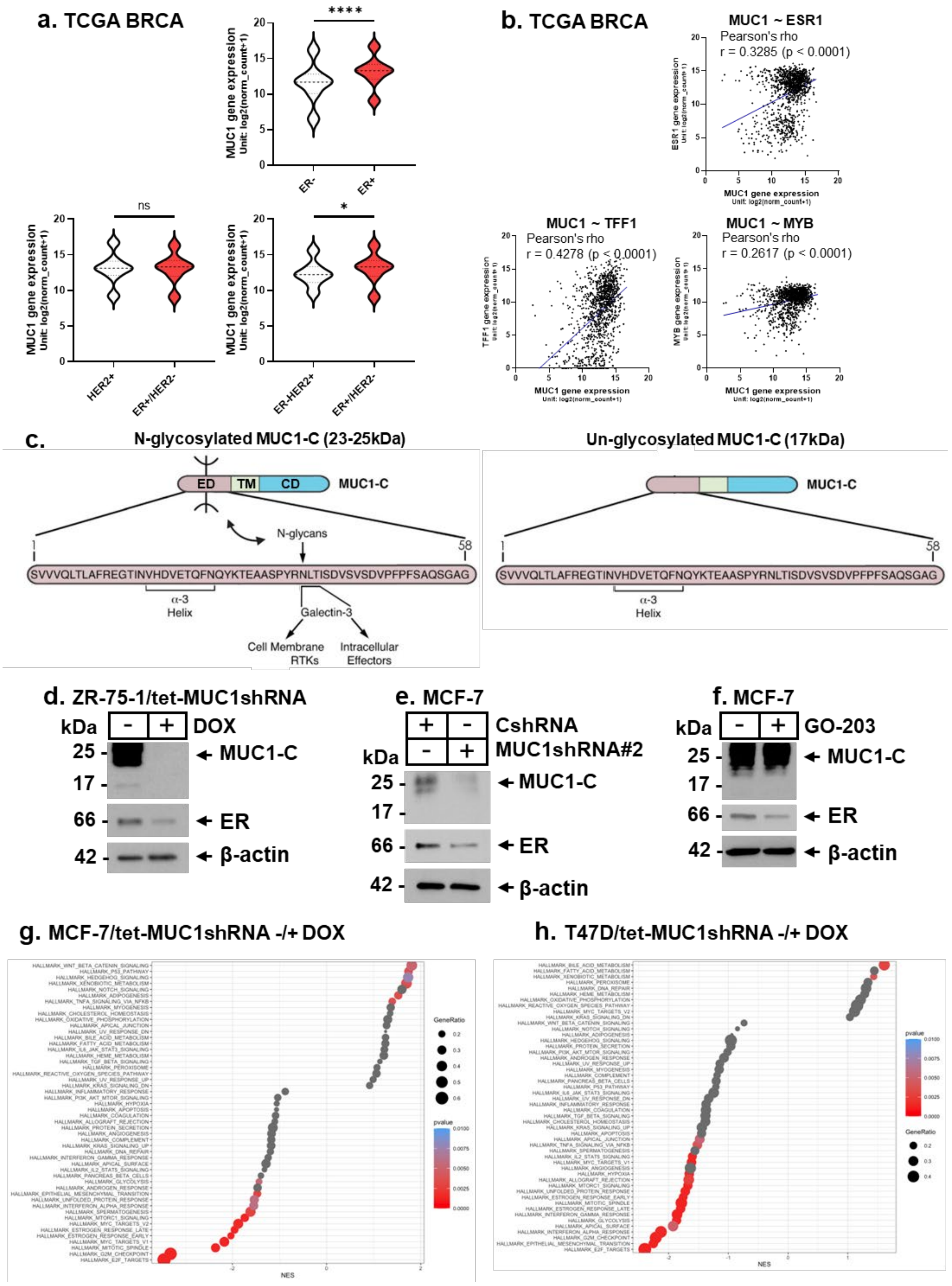

Supplemental Figure S1. Association of MUC1 with ESR1 expression and HALLMARK gene signatures. a. Analysis of the TCGA BRCA dataset for MUC1 expression in (i) ER+ vs ER-, and (ii) HER2+ vs HER2- BCs. **b.** Association of *MUC1* with expression of *ESR1* and ER target genes *TFF1* and *MYB* in the TCGA BRCA dataset. **c.** Schema of MUC1-C structure with the 58 aa extracellular domain (ED), 28 aa transmembrane domain (TD) and 72 aa cytoplasmic domain (CD). Modification of the ED by N-glycosylation at the NLT motif functions as galectin-3 binding site and thereby interactions with RTKs at the cell membrane, as well as intracellular effectors. N-glycosylated MUC1-C is detectable as a ~23-25 kDa glycoprotein (left). Unglycosylated MUC1-C is detectable as a 17 kDa protein (right). The MUC1-C ED alpha-3 helix is the ADC target. **d.** Lysates from ZR-75-1/tet-MUC1shRNA cells treated with vehicle or DOX for 7 days were immunoblotted with antibodies against the indicated proteins. **e.** Lysates from MCF-7/CshRNA and MCF-7/MUC1shRNA#2 cells were immunoblotted with antibodies against the indicated proteins. **f.** Lysates from MCF-7 cells treated with 2.5 μM GO-203 for 3 days were immunoblotted with antibodies against the indicated proteins. **g and h.** GSEA of RNA-seq data from MCF-7 (**g**) and T47D (**h**) cells with MUC1-C silencing using the indicated HALLMARK gene signatures.

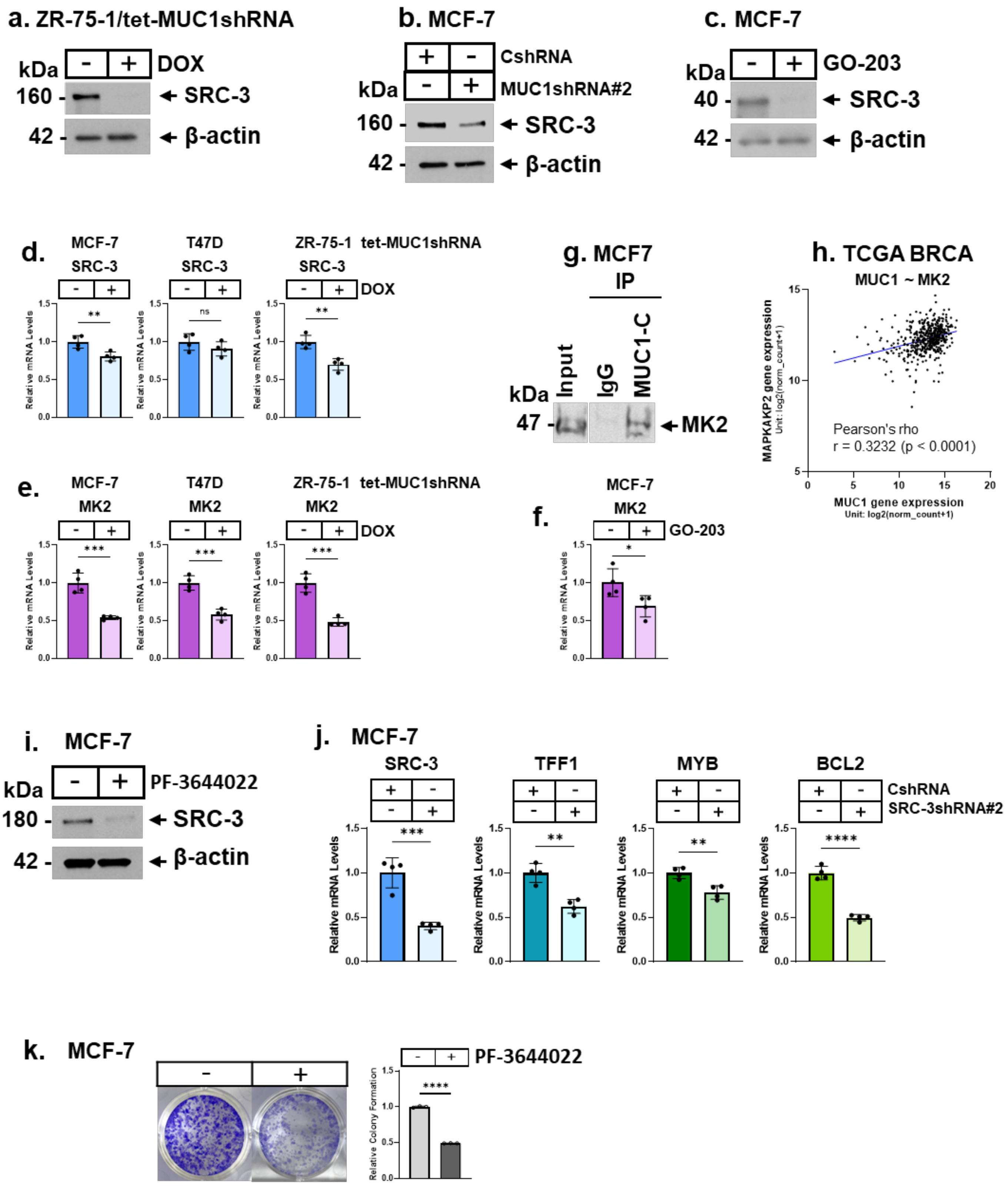

Supplemental Fig. S2. MUC1-C regulates SRC-3 expression. a. Lysates from ZR-75-1/tet-MUC1shRNA cells treated with vehicle or DOX for 7 days were immunoblotted with antibodies against the indicated

proteins. **b.** Lysates from MCF-7/CshRNA and MCF-7/MUC1shRNA#2 cells were immunoblotted with antibodies against the indicated proteins. **c.** Lysates from MCF-7 cells treated with 5 μM GO-203 for 4 days were immunoblotted with antibodies against the indicated proteins. **d.** MCF-7/tet-MUC1shRNA, T47D/tet-MUC1shRNA and ZR-75-1/tet-MUC1shRNA cells treated with vehicle or DOX for 7 days were analyzed for SRC-3 transcripts by qRT-PCR. The results (mean±SD of 4 determinations) are expressed as relative levels compared to that obtained for vehicle-treated cells (assigned a value of 1). **e.** The indicated cells expressing tet-MUC1shRNA treated with vehicle or DOX for 7 days were analyzed for MK2 transcripts. The results (mean±SD of 4 determinations) are expressed as relative levels compared to that obtained for vehicle-treated cells (assigned a value of 1). **f.** MCF-7 cells treated with vehicle or 2.5 μM GO-203 for 4 days were analyzed for MK2 transcripts. The results (mean±SD of 4 determinations) are expressed as relative levels compared to that obtained for vehicle-treated cells (assigned a value of 1). **g.** Lysates from E2-stimulated MCF-7 cells were immunoprecipitated with anti-MUC1-C and a control IgG. The input (10% of total lysate) and precipitates were immunoblotted with anti-MK2. **h.** Association of MUC1 and MK2 in the TCGA BRCA dataset. **i.** Lysates from MCF-7 treated with vehicle or 5 μM PF-3644022 for 24 hours were immunoblotted against the indicated proteins. **j.** MCF-7/CshRNA and MCF-7/SRC-3shRNA#2 cells were analyzed for the indicated transcripts by qRT-PCR. The results (mean±SD of 4 determinations) are expressed as relative levels compared to that obtained for CshRNA cells (assigned a value of 1). **k.** MCF-7 cells treated with vehicle or 5 μM PF-3644022 were analyzed for colony formation. Shown are representative photomicrographs of stained colonies (left). The results (mean±SD of three determinations) are expressed as relative colony formation compared to that for vehicle-treated cells (assigned a value of 1)(right).

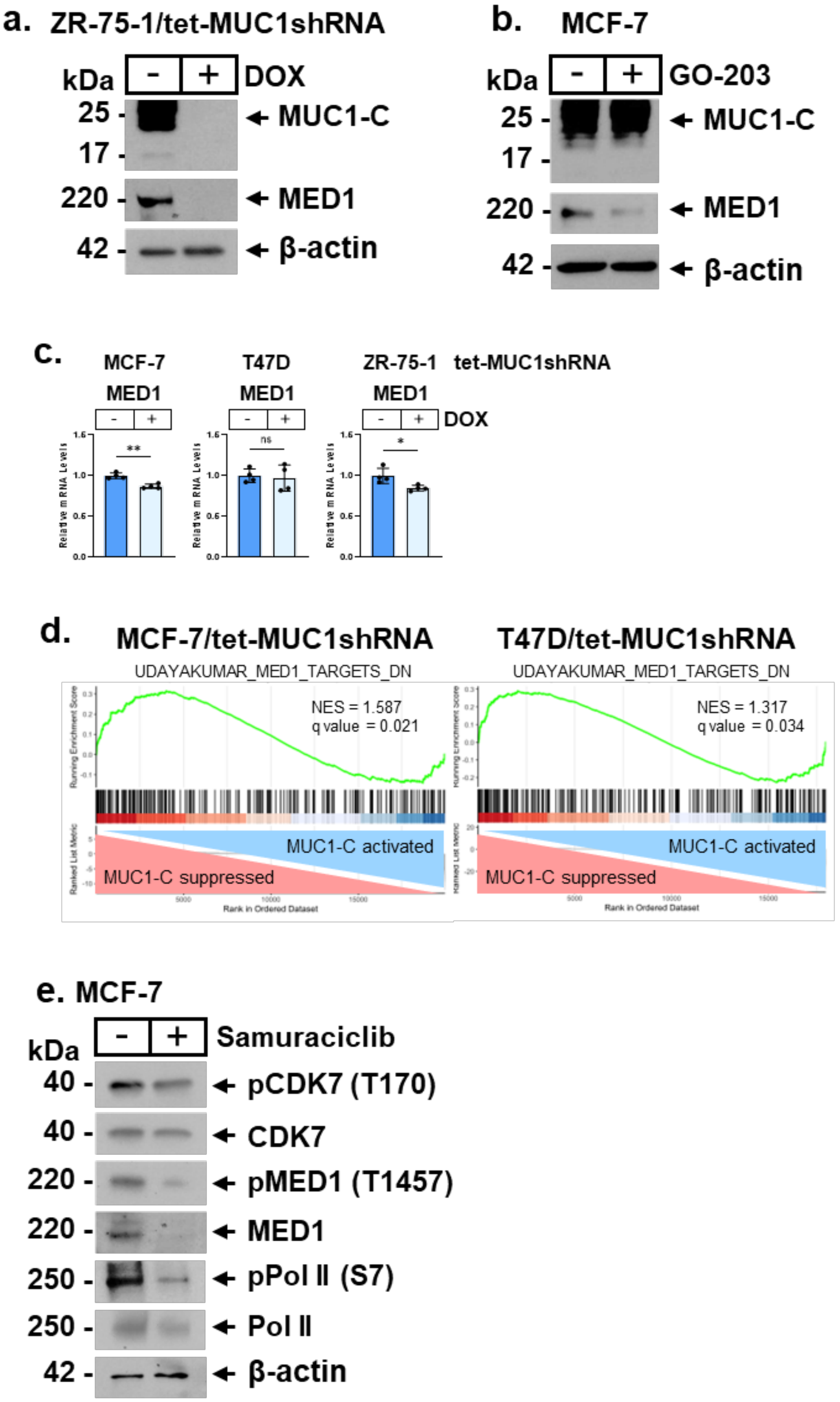

Supplemental Figure S3. MUC1-C regulates MED1 expression. a. Lysates from ZR-75-1/tet-MUC1shRNA cells treated with vehicle or DOX for 7 days were immunoblotted with antibodies against the indicated proteins. **b.** Lysates from MCF-7 cells treated with 5 μM GO-203 for 4 days were immunoblotted with antibodies against the indicated proteins. **c.** MCF-7/tet-MUC1shRNA, T47D/tet-MUC1shRNA and ZR-75-1/tet-

MUC1shRNA cells treated with vehicle or DOX for 7 days were analyzed for MED1 transcripts by qRT-PCR. The results (mean±SD of 4 determinations) are expressed as relative levels compared to that obtained for vehicle-treated cells (assigned a value of 1). **d.** GSEA of RNA-seq data from MCF-7 and T47D cells with MUC1-C silencing using the UDAYAKUMAR MED1 TARGETS DN gene signature. **e.** Lysates from MCF-7 cells treated with vehicle or 1 μM samuraciclib for 48 hours were immunoblotted with antibodies against the indicated proteins.

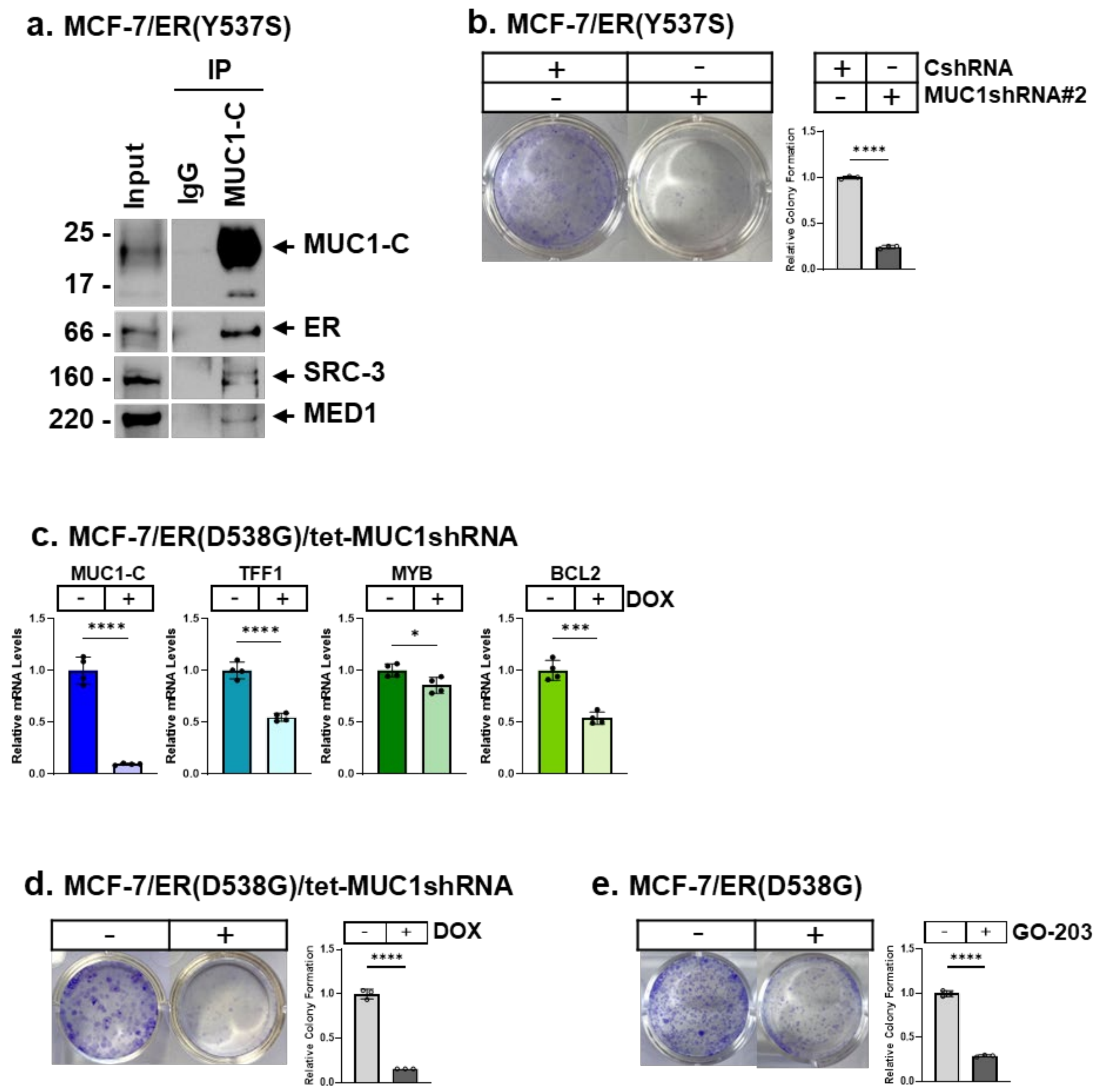

Supplemental Figure S4. MCF-7(D538G) cells are dependent on MUC1-C for survival. a. Nuclear lysates from MCF-7(Y537S) cells grown in hormone deprived conditions for 2 days and then stimulated with 100 nM E2 for 3 hours were precipitated with anti-MUC1-C or a control IgG. The precipitates and input lysate were immunoblotted with antibodies against the indicated proteins. **b.** MCF-7/ER(Y537S)/CshRNA and MCF-7/ER(Y537S)/MUC1shRNA#2 cells were analyzed for colony formation. Shown are representative photomicrographs of stained colonies (left). The results (mean±SD of three determinations) are expressed as relative colony formation compared to that for CshRNA cells (assigned a value of 1)(right). **c.** MCF-7/ER(D538G)/tet-MUC1shRNA cells treated with

vehicle or DOX for 7 days were analyzed for the indicated transcripts by qRT-PCR. The results (mean±SD of 4 determinations) are expressed as relative levels compared to that obtained for vehicle-treated cells (assigned a value of 1). **d.** MCF-7/ER(D538G)/tet-MUC1shRNA cells treated with vehicle or DOX for 7 days were analyzed for colony formation. Shown are representative photomicrographs of stained colonies (left). The results (mean±SD of three determinations) are expressed as relative colony formation compared to that for vehicle-treated cells (assigned a value of 1)(right). **e.** MCF-7/ER(D538G) cells treated with vehicle or 5 μM GO-203 were analyzed for colony formation. Shown are representative photomicrographs of stained colonies (left). The results (mean±SD of three determinations) are expressed as relative colony formation compared to that for vehicle-treated cells (assigned a value of 1)(right).

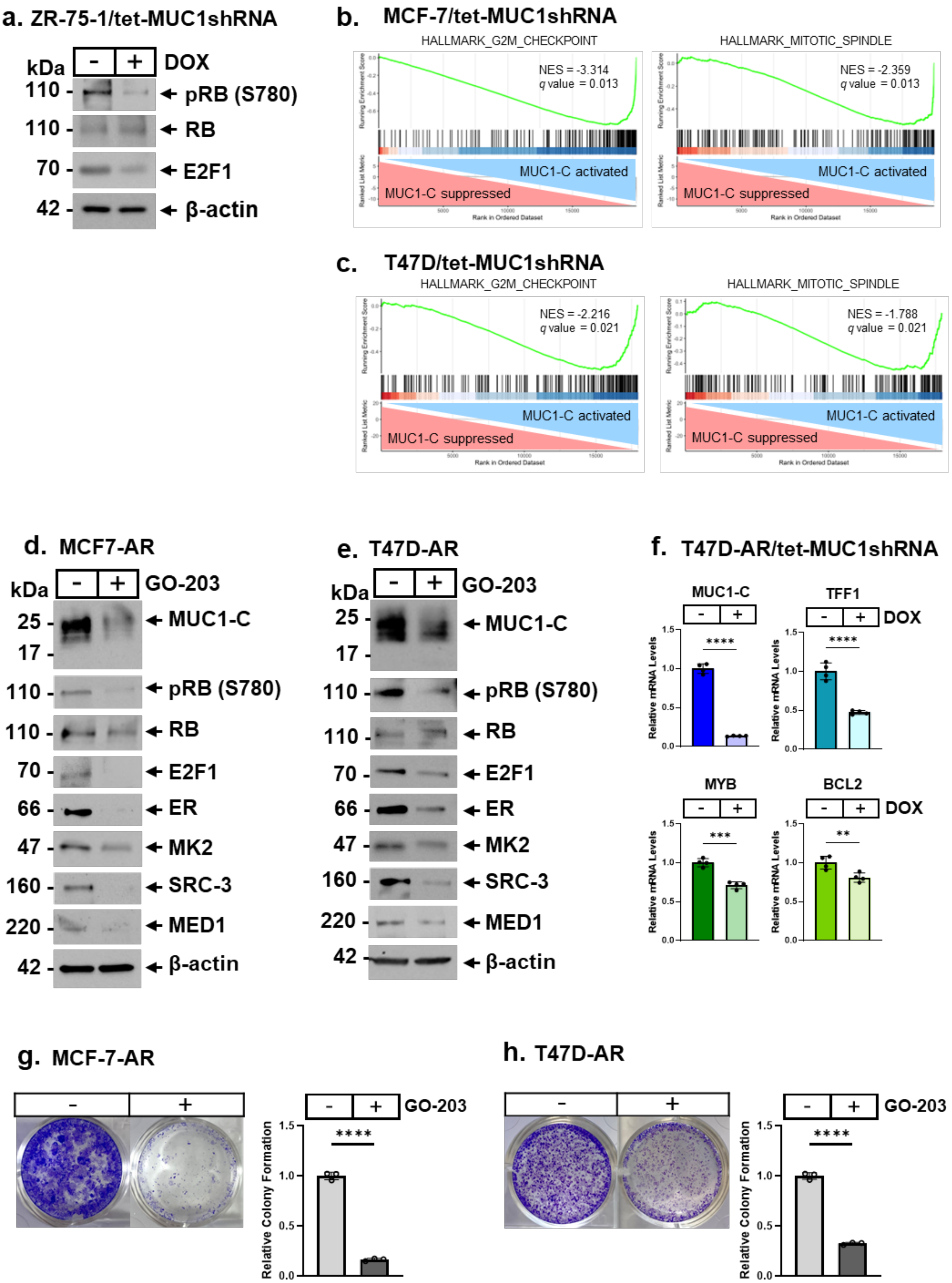
a. ZR-75-1/tet-MUC1shRNA
kDa
- + DOX
110 - pRB (S780)
110 - RB
70 - E2F1
42 - β-actin
b. MCF-7/tet-MUC1shRNA
HALLMARK_G2M_CHECKPOINT
NES = -3.314
q value = 0.013
MUC1-C activated
MUC1-C suppressed
Rank in Ordered Dataset
HALLMARK_MITOTIC_SPINDLE
NES = -2.359
q value = 0.013
c. T47D/tet-MUC1shRNA
HALLMARK_G2M_CHECKPOINT
NES = -2.216
q value = 0.021
HALLMARK_MITOTIC_SPINDLE
NES = -1.788
q value = 0.021
d. MCF7-AR
e. T47D-AR
- + GO-203
25 - MUC1-C
17 -
110 - pRB (S780)
110 - RB
70 - E2F1
66 - ER
47 - MK2
160 - SRC-3
220 - MED1
42 - β-actin
f. T47D-AR/tet-MUC1shRNA
MUC1-C
TFF1
MYB
BCL2
- + DOX
Relative mRNA Levels
****
***
**
g. MCF-7-AR
h. T47D-AR
- + GO-203
Relative Colony Formation
****

Supplemental Figure S5. MCF-7-AR and T47D-AR cells are MUC1-C dependent. a. Lysates from ZR-75-1/tet-MUC1shRNA cells treated with vehicle or DOX for 7 days were immunoblotted with antibodies against the indicated proteins. **b and c.** GSEA of RNA-seq data from MCF-7 cells (**b**) and T47D cells (**c**) with MUC1-C silencing using the indicated HALLMARK gene signatures. **d and e.** MCF-7-AR (**d**) and T47D-AR (**e**) cells treated with vehicle or 5 μM GO-203 for 3 days were immunoblotted with antibodies against the indicated proteins. **f.** T47D-AR/tet-MUC1shRNA cells treated with vehicle or DOX for 7 days were analyzed for the indicated transcripts by qRT-PCR. The results (mean±SD of 4 determinations) are expressed as relative levels compared to that obtained for vehicle-treated cells (assigned a value of 1). **g and h.** MCF-7-AR (**g**) and T47D-AR (**h**) cells treated with vehicle or 5 μM GO-203 were analyzed for colony formation. Shown are representative photomicrographs of stained colonies (left). The results (mean±SD of three determinations) are expressed as relative colony formation compared to that for vehicle-treated cells (assigned a value of 1)(right).

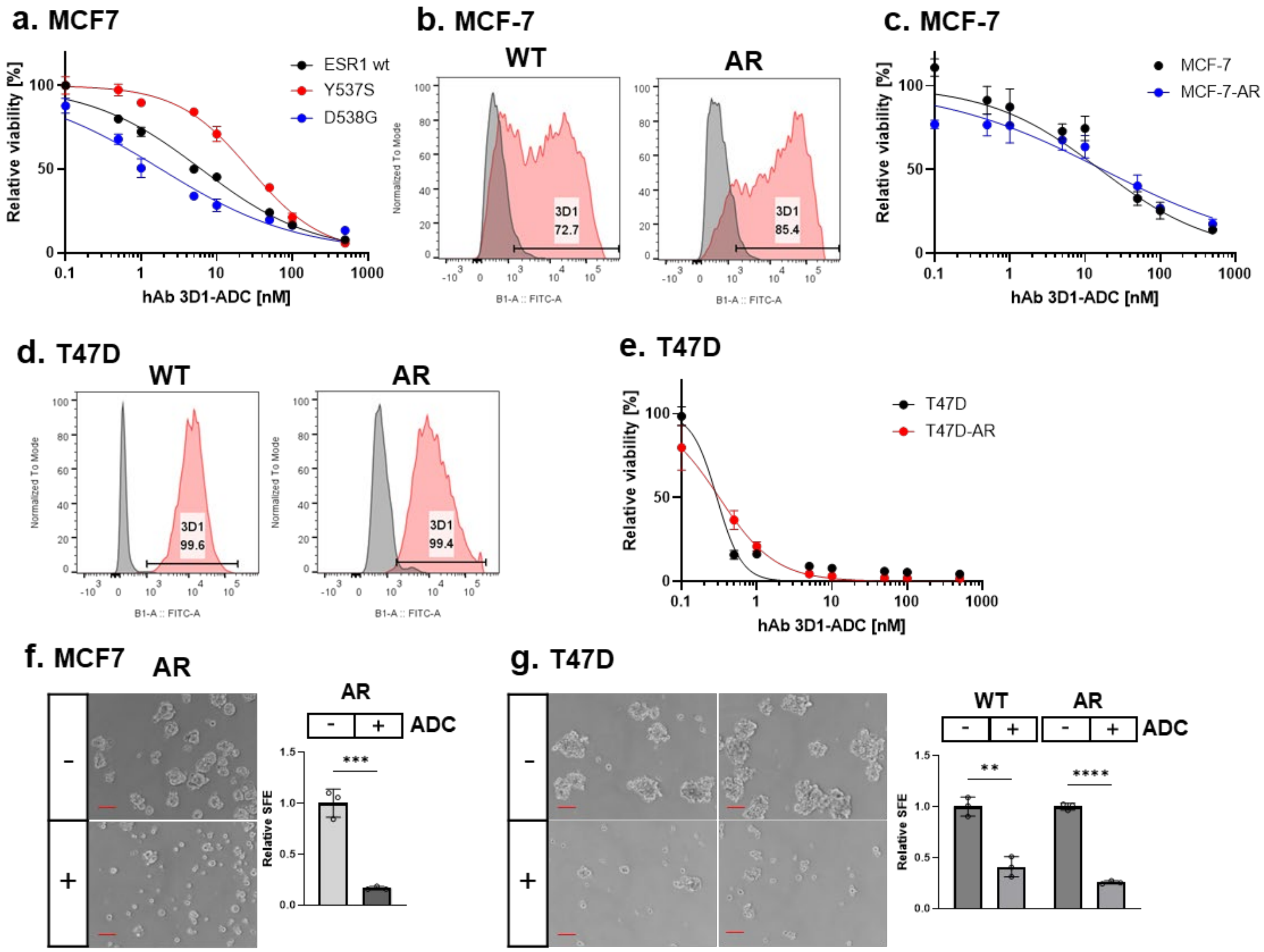

Supplemental Figure S6. Cell surface MUC1-C expression and anti-MUC1-C ADC dose-response curves. a. The designated MCF-7 cells were treated with the indicated concentrations of anti-MUC1-C ADC for 7 days and analyzed for cell viability by Alamar Blue staining. The results (mean±SD of three determinations) are expressed as relative cell viability (% control) compared with that for untreated cells. **b.** The designated MCF-7 cells were analyzed for cell surface MUC1-C expression by flow cytometry. **c.** The designated MCF-7 cells were treated with the indicated concentrations of anti-MUC1-C ADC for 7 days and analyzed for cell viability by Alamar Blue staining. The results (mean ± SD of three determinations) are expressed as relative cell viability (% control) compared with that for untreated cells. **d.** The designated T47D cells were analyzed for cell surface MUC1-C

expression by flow cytometry. **e.** The designated T47D cells were treated with the indicated concentrations of anti-MUC1-C ADC for 7 days and analyzed for cell viability by Alamar Blue staining. The results (mean ± SD of three determinations) are expressed as relative cell viability (% control) compared with that for untreated cells. **f and g**. The designated MCF-7 (**f**) and T47D (**g**) cells treated with 100 nM anti-MUC1-C ADC were analyzed for tumorsphere formation. Shown are representative photomicrographs of tumorspheres (left). The results (mean±SD of three determinations) are expressed as relative sphere formation efficiency (SFE) compared to that for control cells (assigned a value of 1)(right).

Supplemental Tables

Supplemental Table S1. Common down-regulated ERGs and LRGs in MCF-7 and T47D cells with MUC1-C silencing.

**ERGs**	**LRGs**
CA12	CA12
CD44	CD44
ELOVL2	KIF20A
GFRA1	MYB
GREB1	RAB31
MUC1	SLC7A5
MYB	TOP2A
MYBL1	
RAB31	
SLC7A5	
UGCG	

Supplemental Table S2. Primers used for qRT-PCR.

**MUC1-C**	**FWD**	TACCGATCGTAGCCCCTATG
	**REV**	CTCACCAGCCCAAACAGG
**TFF1**	**FWD**	CCCTCCCAGTGTGCAAATAAG
	**REV**	GAACGGTGTCGTCGAAACAG
**MYB**	**FWD**	GAAAGCGTCACTTGGGGAAAA
	**REV**	TGTTCGATTCGGGAGATAATTGG
**BCL2**	**FWD**	GGTGGGGTCATGTGTGTGG
	**REV**	CGGTTCAGGTACTCAGTCATCC
**SRC-3**	**FWD**	AGACGGGAGCAGGAAAGTAAA
	**REV**	GTAAAAGCGGTCCTAAGGAGTC
**MK2**	**FWD**	CGCAGTTCCACGTCAAGTC
	**REV**	GGGCGAATTTCTCCTGGGTC
**MED1**	**FWD**	CTGGAACGGCTCCATGCAA
	**REV**	CTTCTCCATGACTTGACGCAC
**β-actin**	**FWD**	GATGAGATTGGCATGGCTTT
	**REV**	CACCTTCACCGTTCCAGTTT

Supplemental Table S3. Clinical characteristics of patients with HR+/HER2- BC tumors stained for MUC1-C expression by IHC.

Data are expressed as number (n) and percentage (%) of tumors unless otherwise specified.

a. HR+/HER2- BC surgical specimens (n=18)

Age at surgery (mean ± SD)	53.4 ± 14.0
male/female	0(0) / 18(100)
T is/1/2/3<br>N 0/1<br>M 0/1	0(0) / 13(72) / 4(22) / 1(5.6)<br>18(100) / 0(0)<br>18(100) / 0(0)
Histology<br>Invasive Ductal Carcinoma/others	20(100) / 0(0)
Neoadjuvant treatment<br>yes/no	0(0) / 18(100)
MUC1-C staining score<br>Apical 0/1+/2+/3+<br>Membrane 0/1+/2+/3+<br>Cytoplasm 0/1+/2+/3+	2(11) / 0(0) / 3(17) / 13(72)<br>4(22) / 7(39) / 7(39) / 0(0)<br>1(5.6) / 8(44) / 7(39) / 3(17)

b. ER mutant recurrent/metastatic tumor biopsy specimens (n=11)

Age at biopsy (mean ± SD)	51.2 ± 10.7
male/female	0(0) / 11(100)
ESR1 mutation<br>Y537S / L536R	10(91) / 1(9.1)
Biopsy site<br>skin/lymph node/liver	4(36) / 3(27) / 4(36)
Previous ET<br>yes/no	11(100) / 0(0)
MUC1-C staining score<br>Apical 0/1+/2+/3+<br>Membrane 0/1+/2+/3+<br>Cytoplasm 0/1+/2+/3+	2(18) / 1(9.1) / 2(18) / 6(55)<br>2(18) / 2(18) / 5(45) / 2(18)<br>0(0) / 4(36) / 4(36) / 3(27)

c. CDK4/6i refractory tumor biopsy specimens (n=5)

CDK4/6i starting age (mean ± SD)	51.2 ± 10.14
male/female	0(0) / 5(100)
CDK4/6i used<br>Palbociclib/Abemaciclib/sequencial(P →A)	2(40) / 0(0) / 3(60)
ET used in combination with CDK4/6i<br>Fulvestrant/Letrozole	3(60) / 2(40)
CDK4/6i administration duration (median, days)	224
Previous ET<br>yes/no	4(80) / 1(20)
Previous chemotherapy<br>yes/no	3(60) / 2(40)
MUC1-C staining score pre-CDK4/6i<br>Apical 0/1+/2+/3+<br>Membrane 0/1+/2+/3+<br>Cytoplasm 0/1+/2+/3+	 1(20) / 0(0) / 1(20) / 3(60)<br>3(60)/ 0(0) / 1(20) / 1(20)<br>2(40) / 1(20) / 2(40) /0(0)
MUC1-C staining score post-CDK4/6i<br>Apical 0/1+/2+/3+<br>Membrane 0/1+/2+/3+<br>Cytoplasm 0/1+/2+/3+	 0(0) / 2(40) / 2(40) / 1(20)<br>0(0) / 1(20) / 3(60) / 1(20)<br>0(0) / 0(0) / 0(0) / 5(100)

Supplemental Figure S7

Uncropped images of western blots

Figure 1c

MUC1-C MCF-7

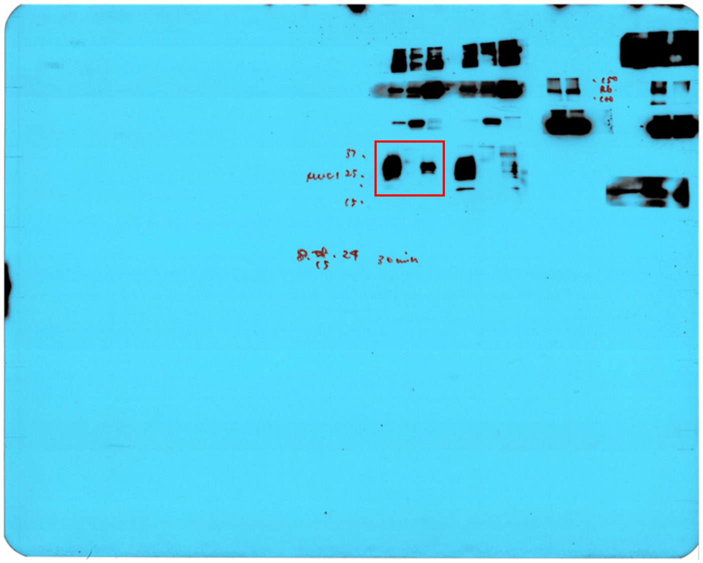

MUC1-C T47D

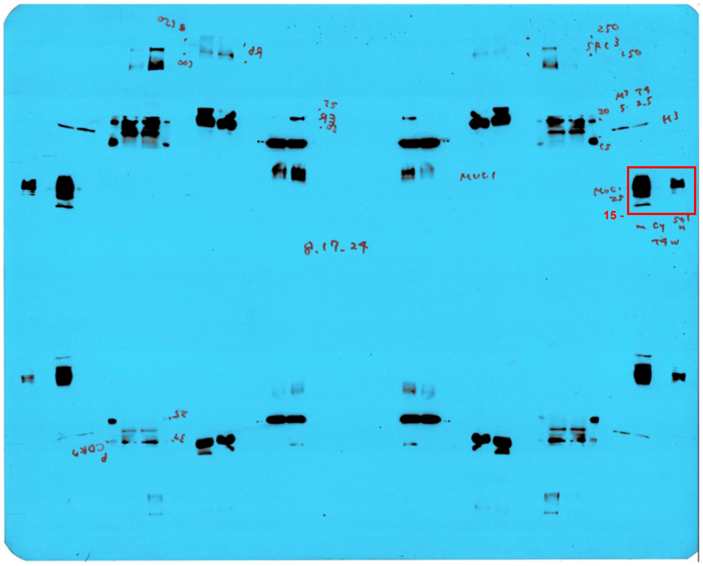

Na,K-ATPase MCF-7

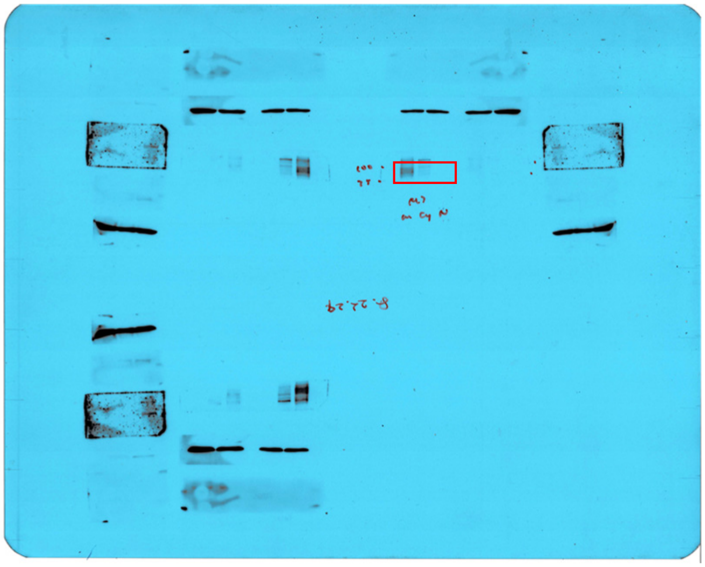

Na,K-ATPase T47D

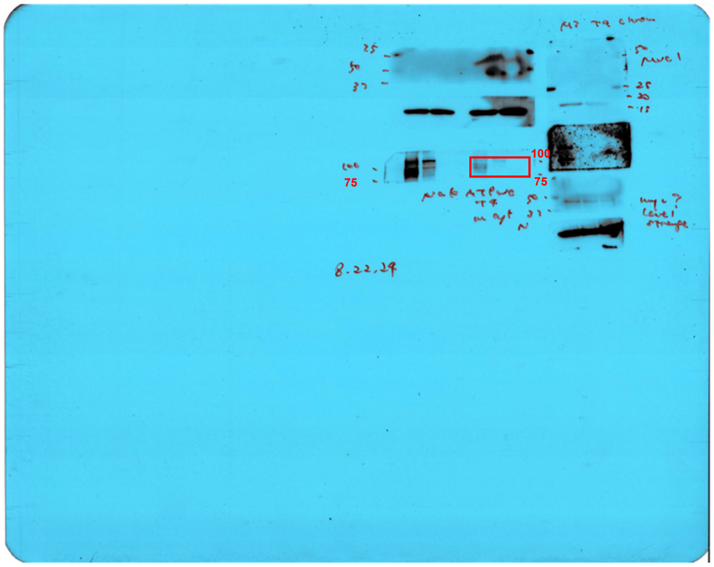

α-tubulin MCF-7 T47D

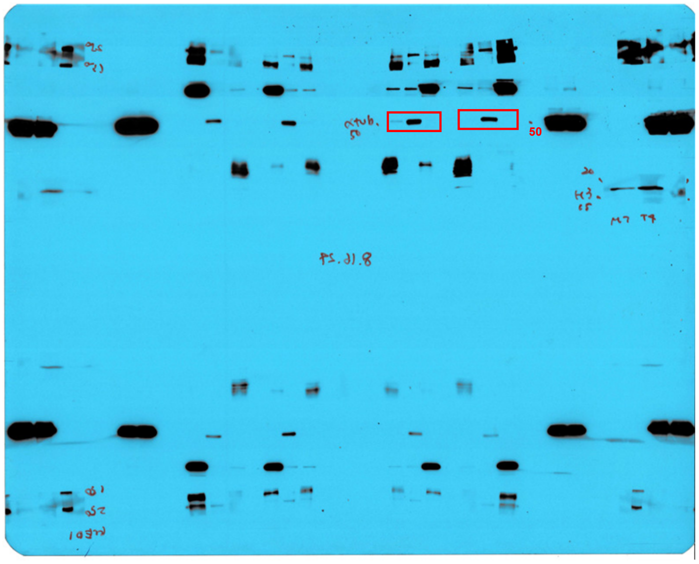

Figure 1c (continued)

LaminB1 **MCF-7**

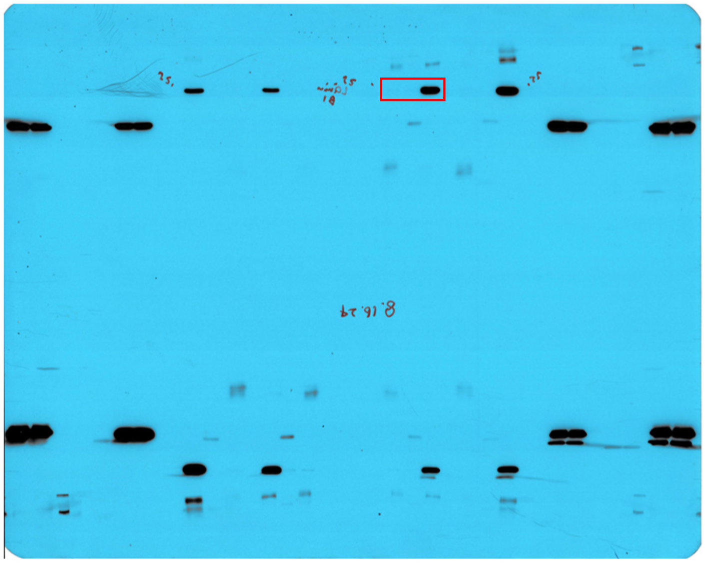

LaminB1 **T47D**

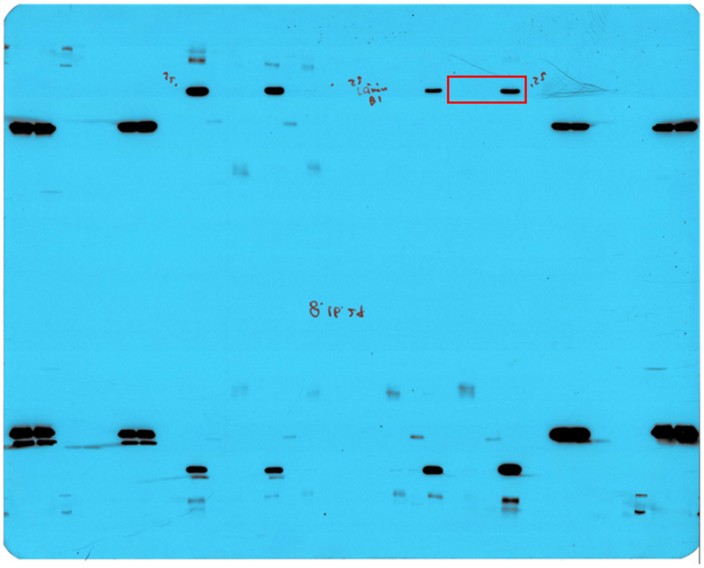

Figure 1d

MUC1-C

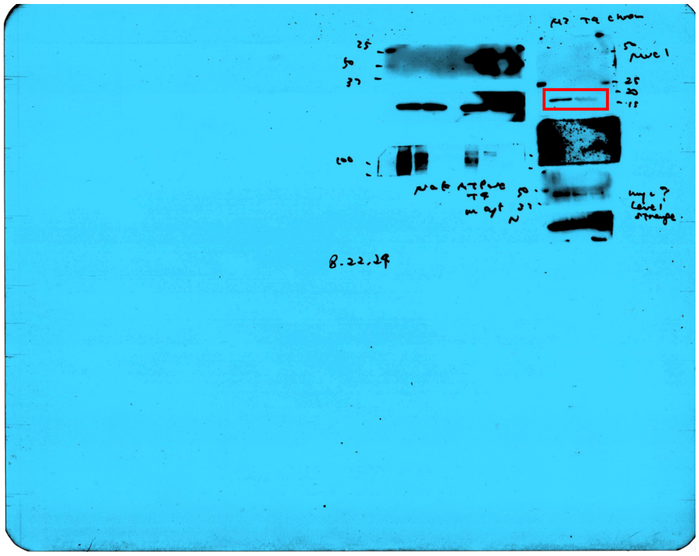

H3

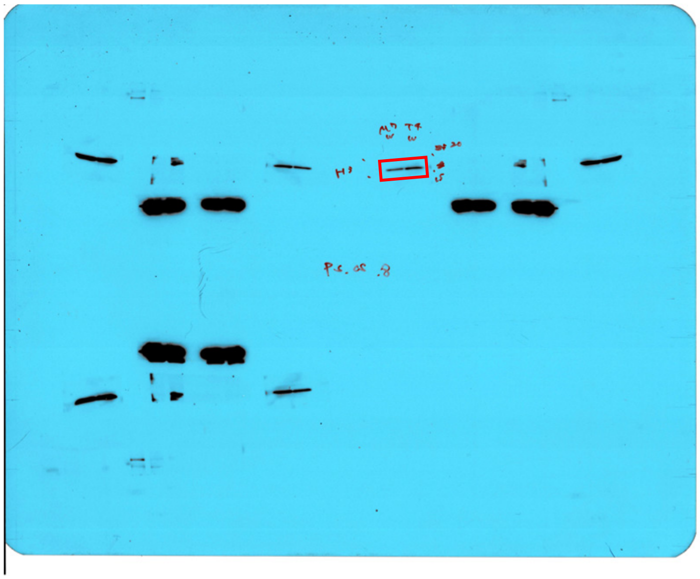

Figure 1e

MUC1-C **MCF-7**

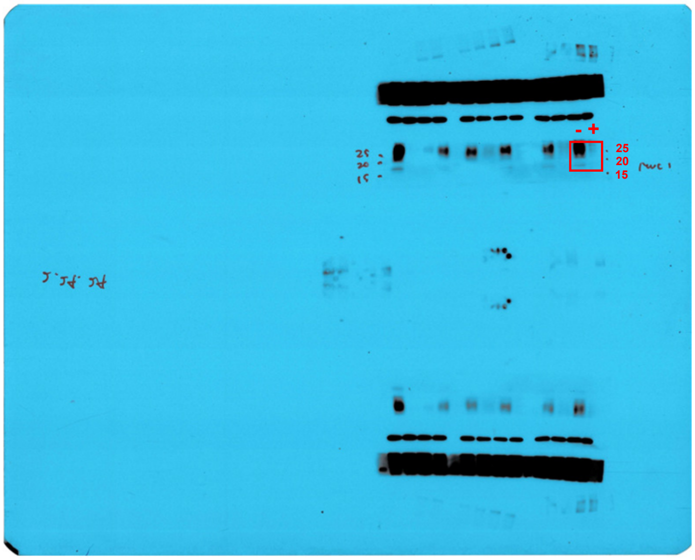

MUC1-C **T47D**

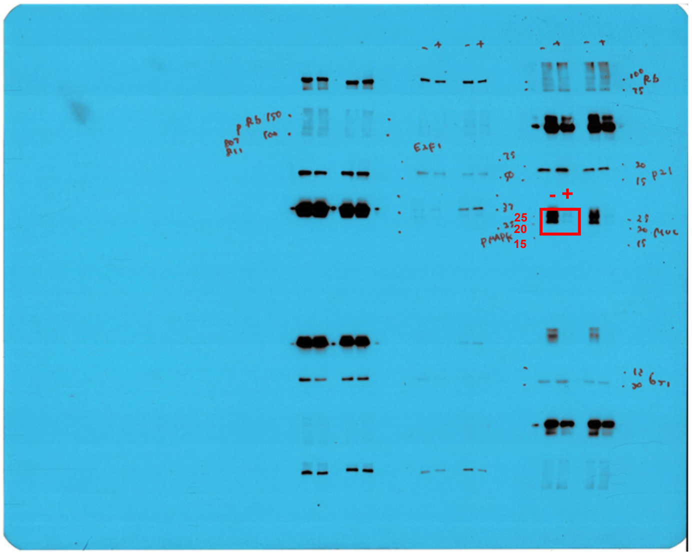

ER **MCF-7**

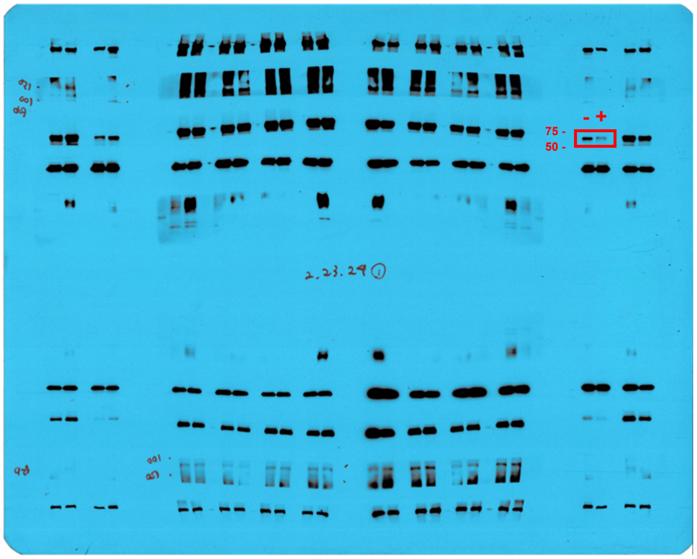

ER **T47D**

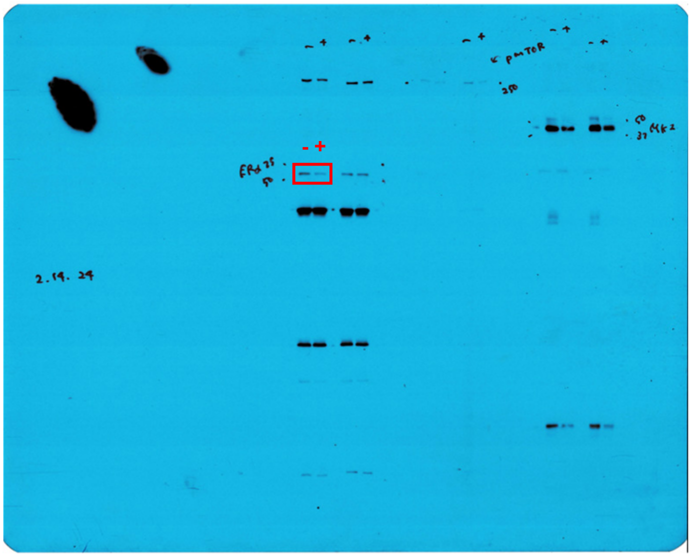

β-actin **MCF-7**

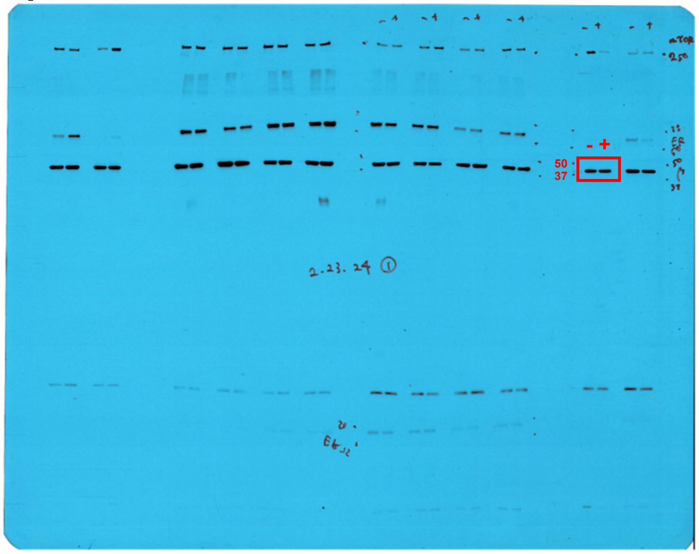

β-actin **T47D**

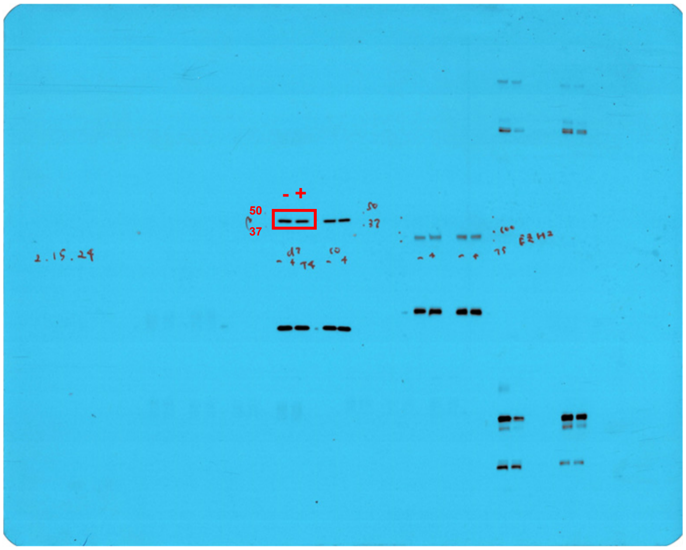

Figure 2a

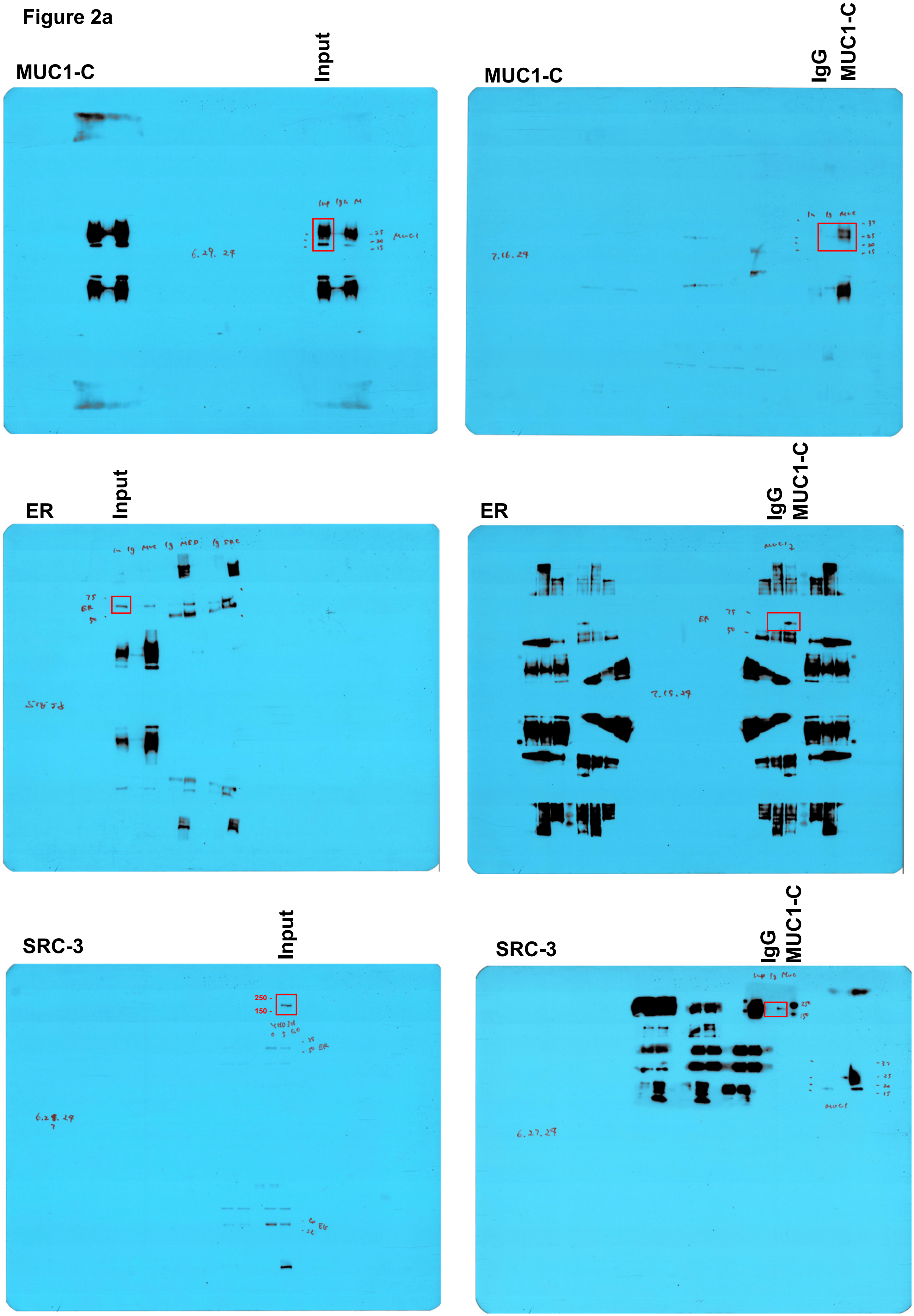
MUC1-C
Input
MUC1-C
IgG
MUC1-C
ER
Input
ER
IgG
MUC1-C
SRC-3
Input
250 -
150 -
SRC-3
IgG
MUC1-C

Figure 2b

SRC-3

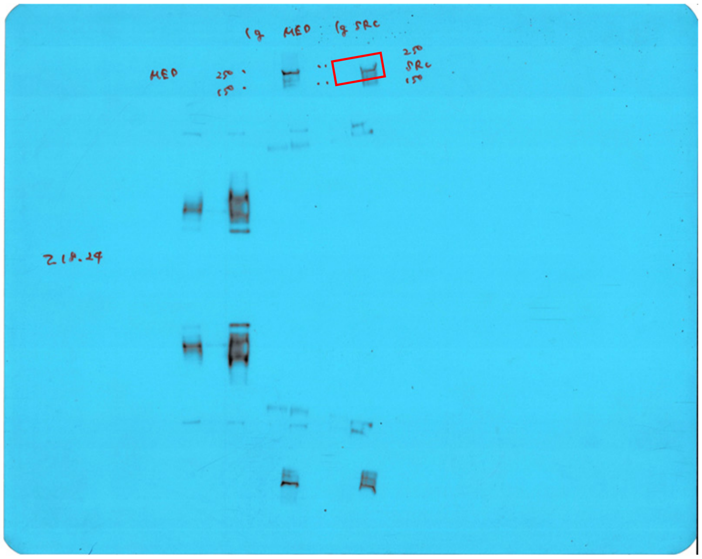

MUC1-C

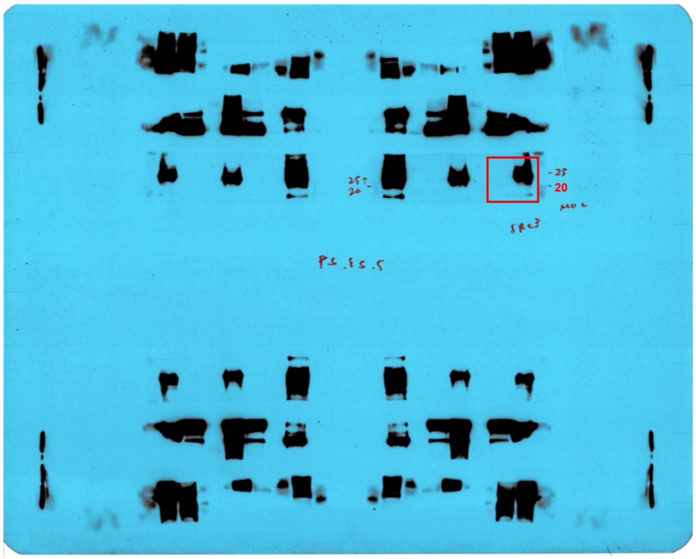

ER

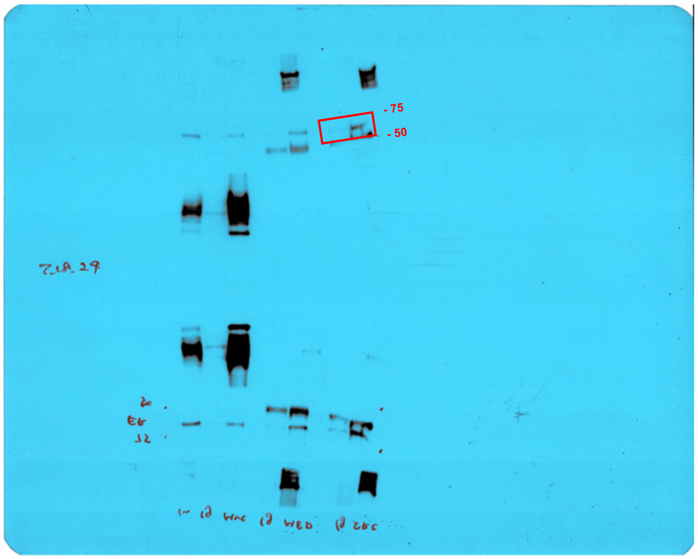

Figure 2c

SRC-3 MCF-7

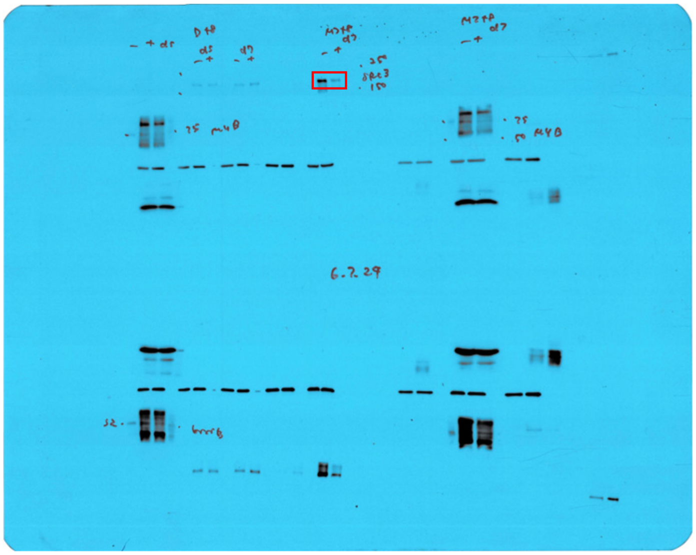

SRC-3 T47D

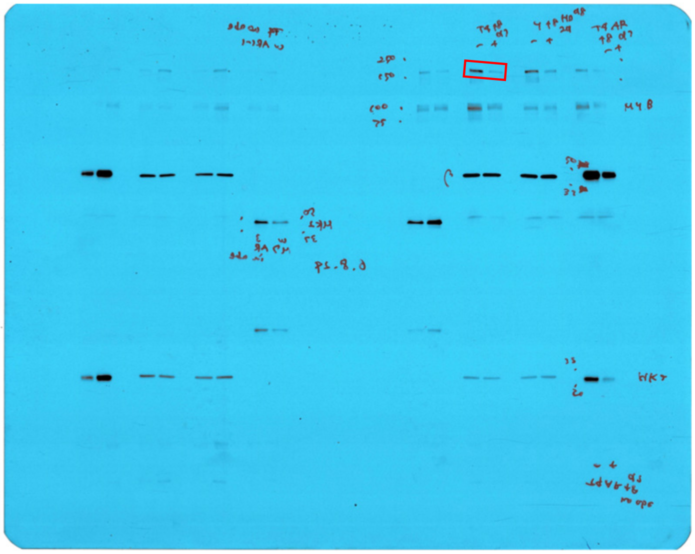

β-actin MCF-7

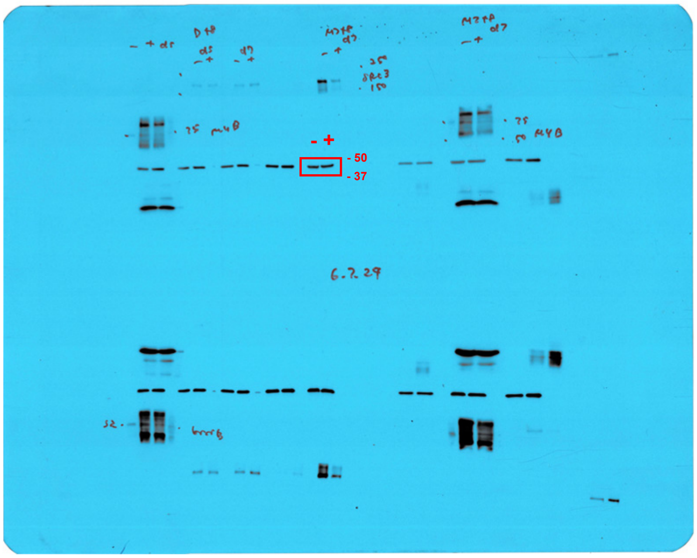

β-actin T47D

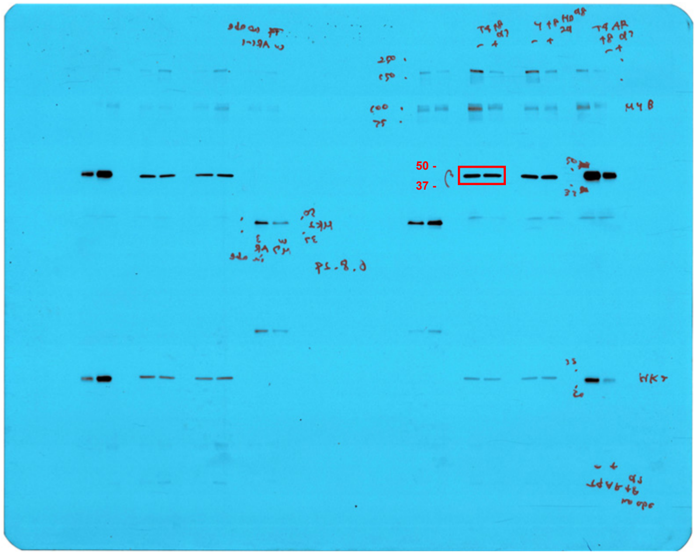

Figure 2d

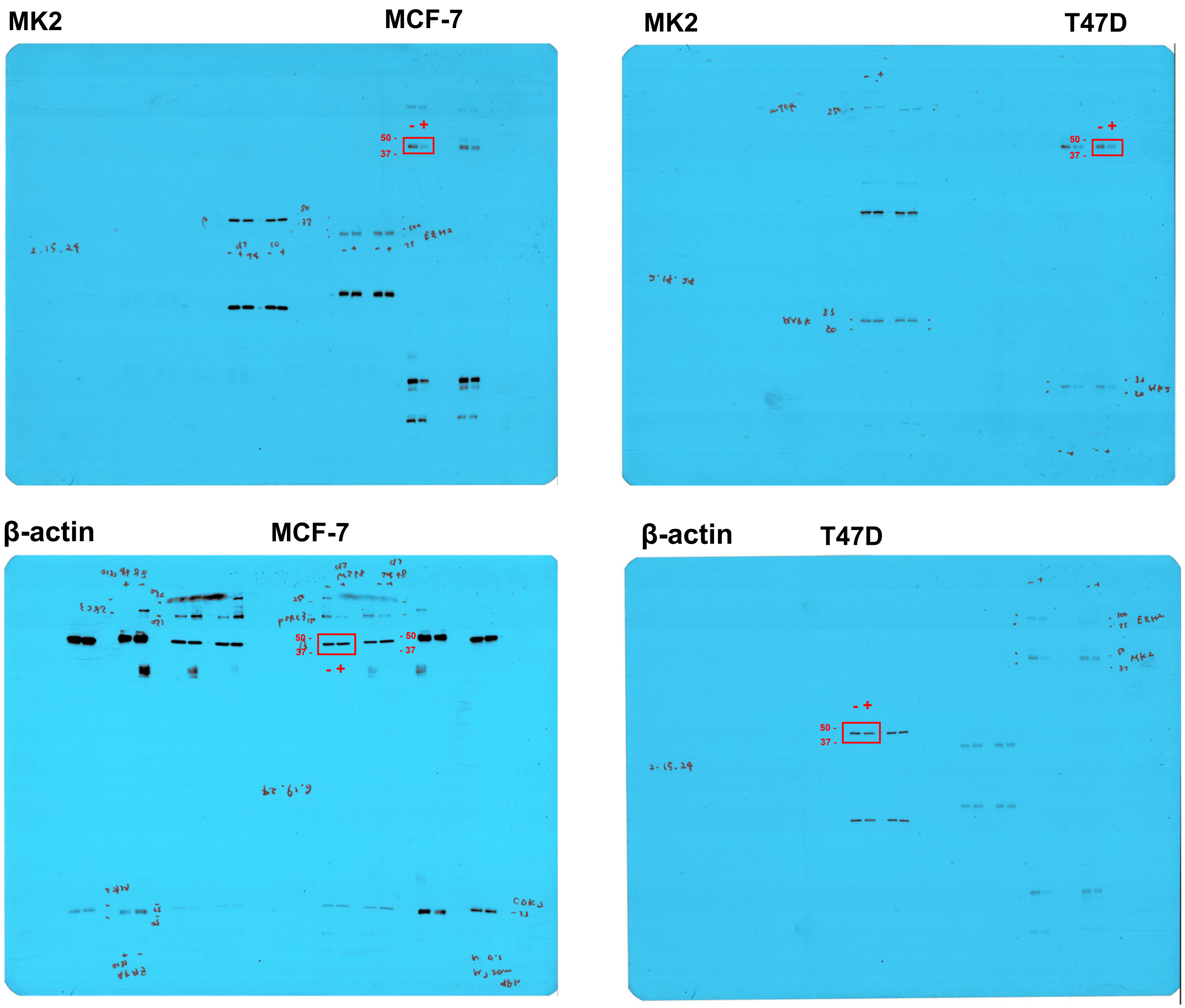
MK2
MCF-7
50 -
37 -
- +
MK2
T47D
50 -
37 -
- +
β-actin
MCF-7
50 -
37 -
- +
- 50
- 37
β-actin
T47D
50 -
37 -
- +

Figure 3a

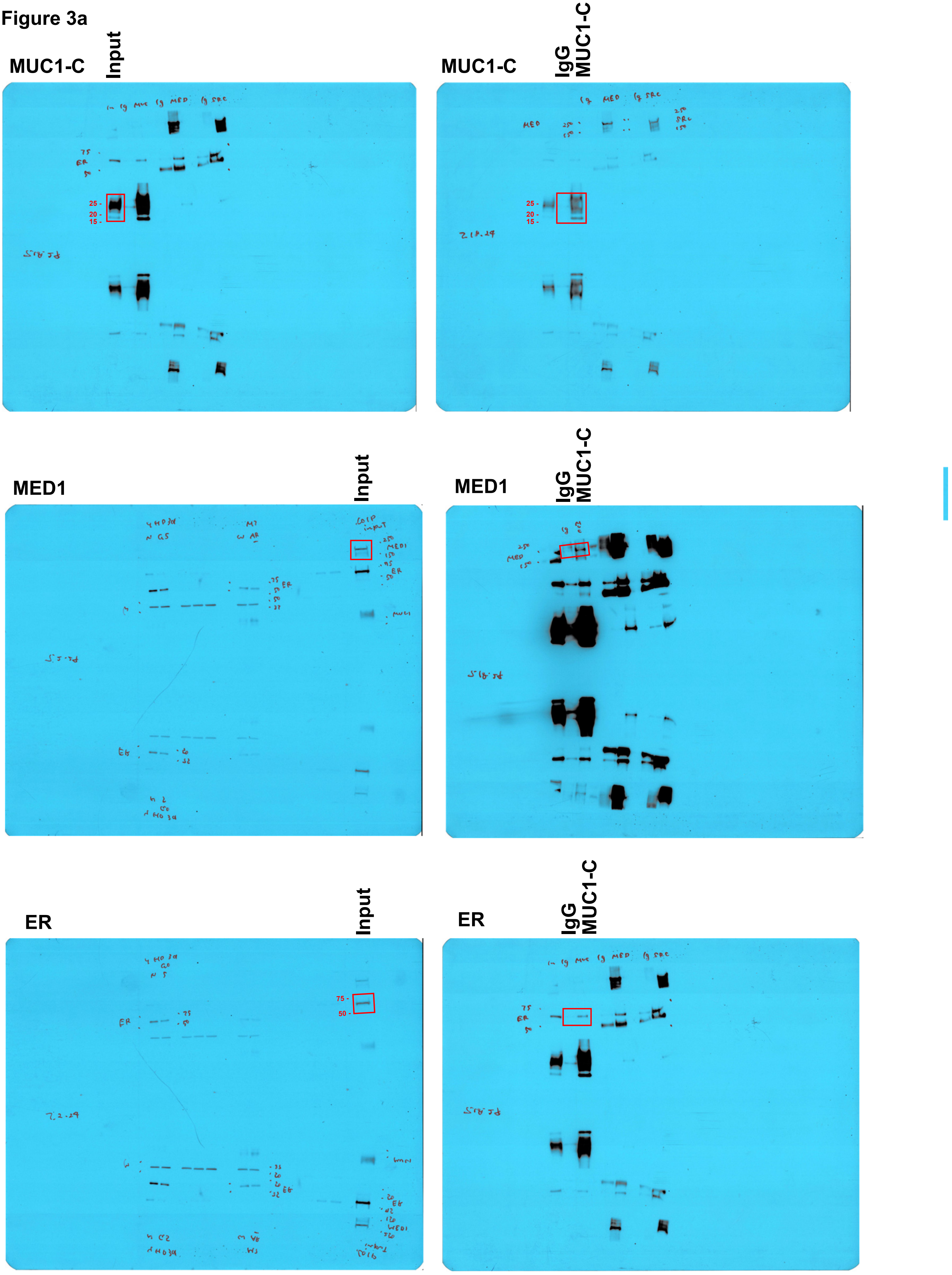

MUC1-C
Input
MUC1-C
IgG
MUC1-C
25 -
20 -
15 -
25 -
20 -
15 -
MED1
Input
MED1
IgG
MUC1-C
ER
Input
75 -
50 -
ER
IgG
MUC1-C

Figure 3b

MED1

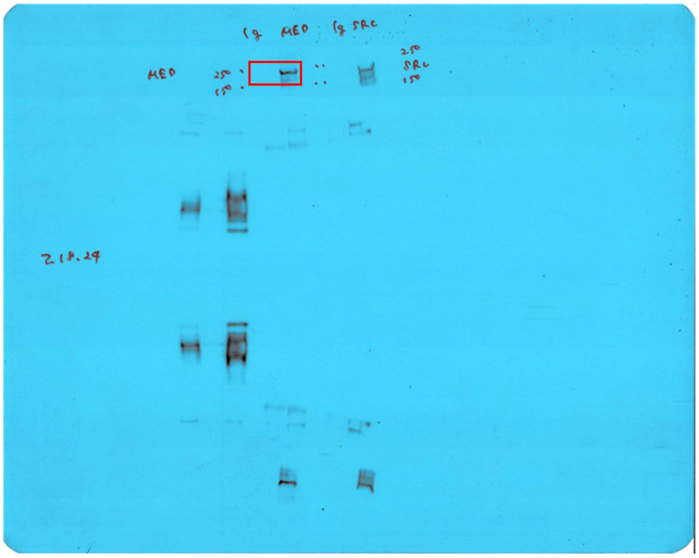

MUC1-C

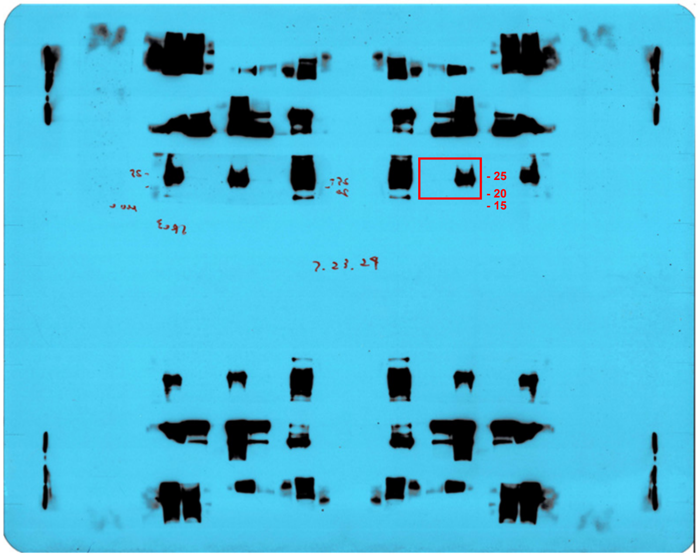

ER

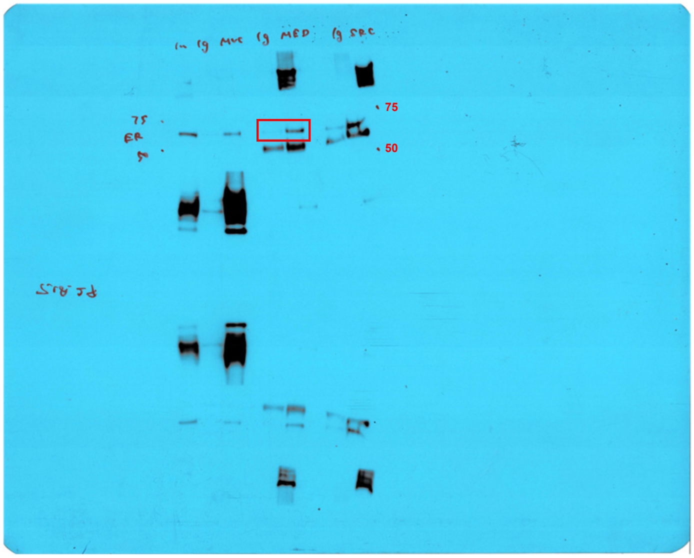

Figure 3c

pMED1 (T1457) MCF-7

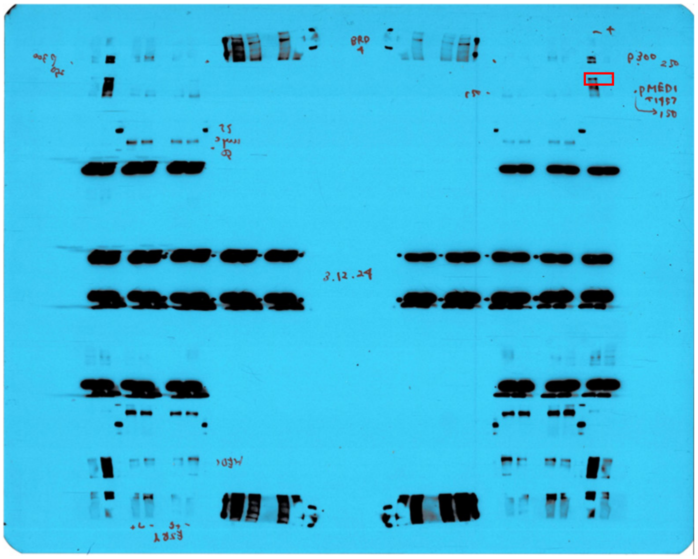

pMED1 (T1457) T47D

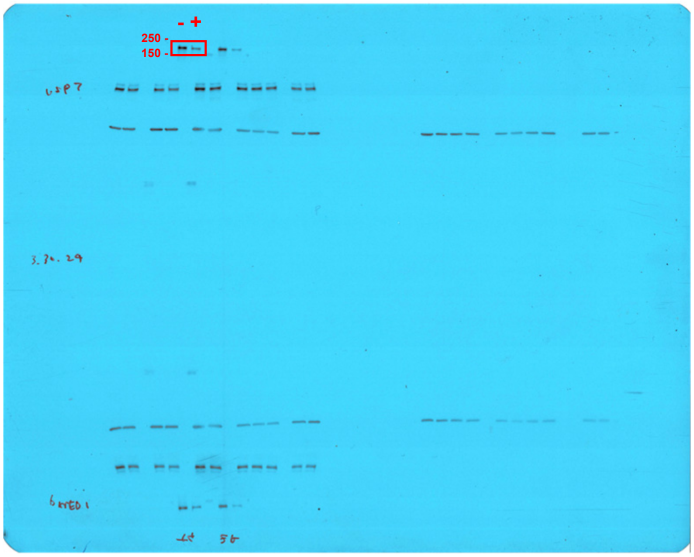

MED1 MCF-7

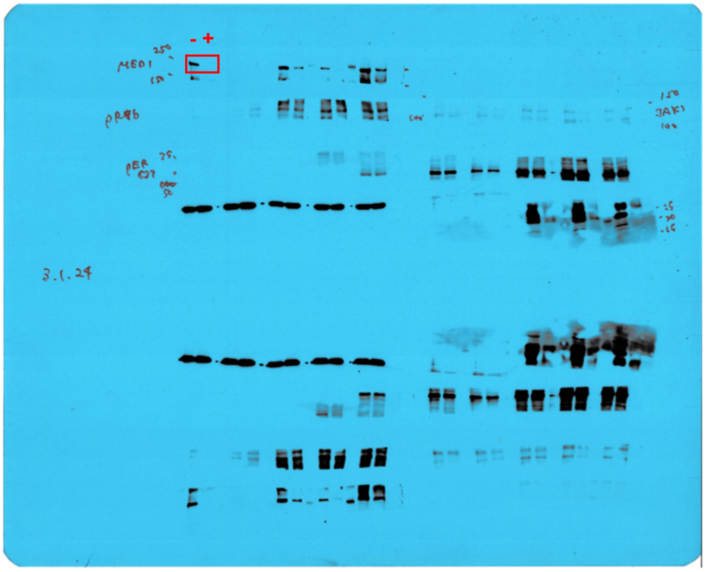

MED1 T47D

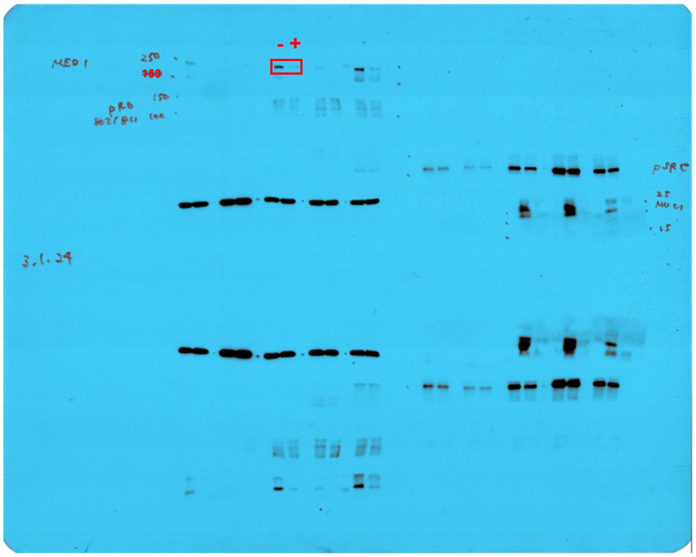

β-actin MCF-7 T47D

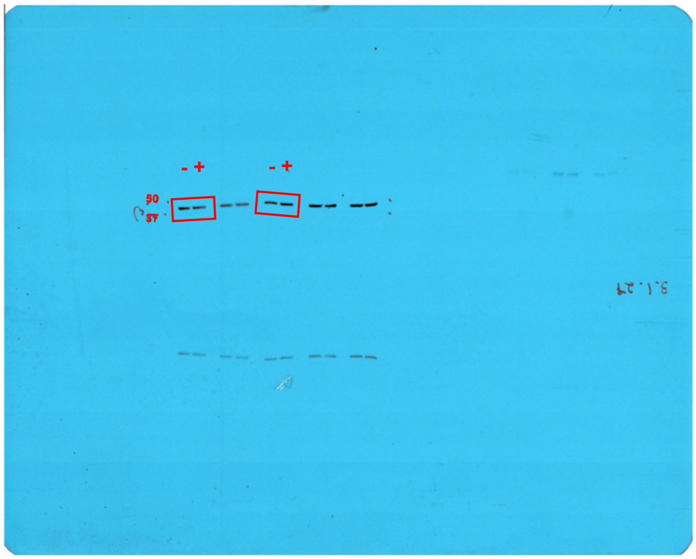

Figure 3d

pCDK7(T170) MCF-7

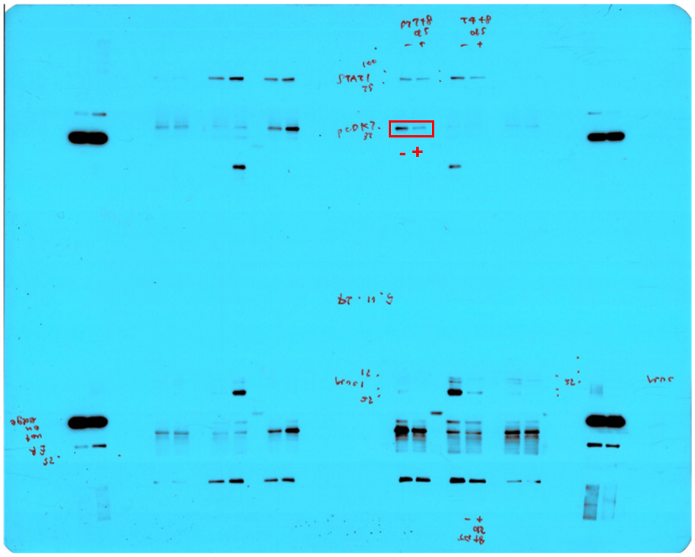

pCDK7(T170) T47D

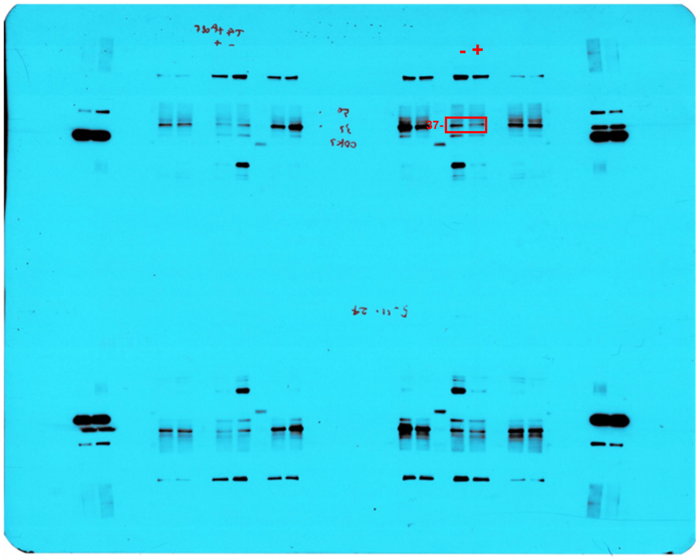

CDK7 MCF-7

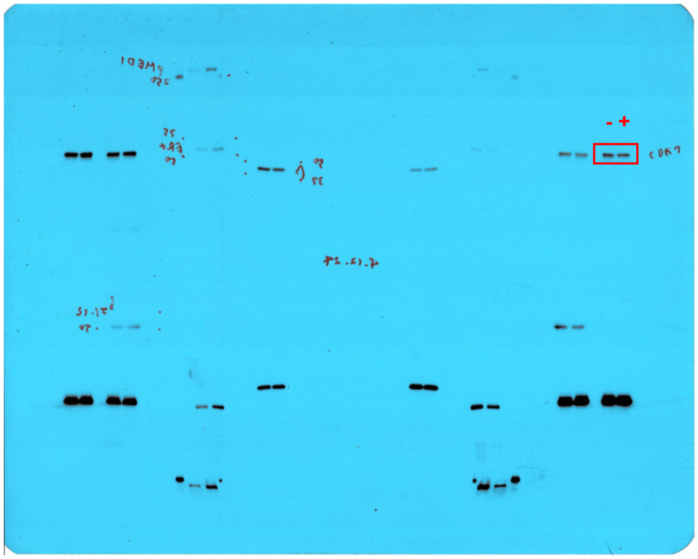

CDK7 T47D

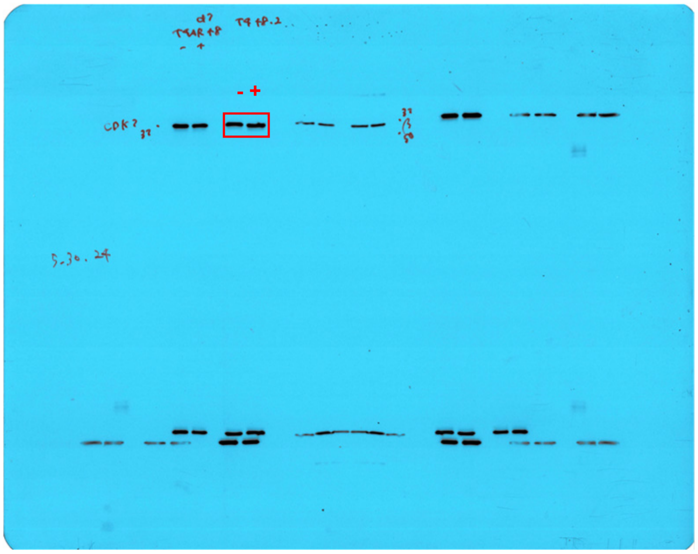

β-actin MCF-7 T47D

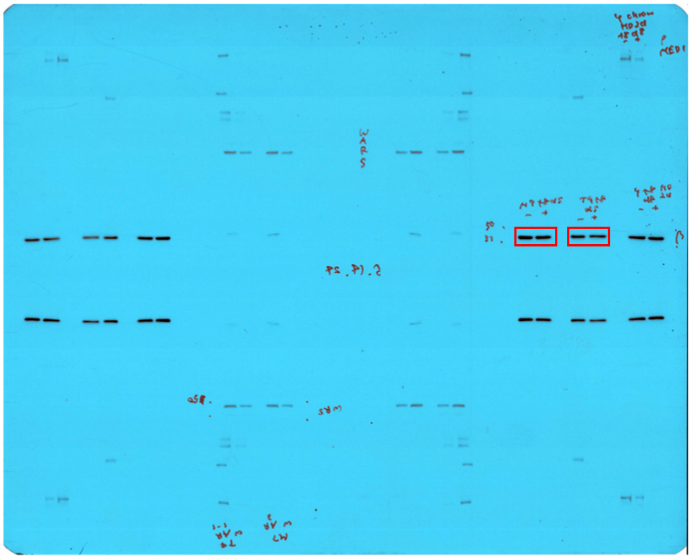

Figure 4a

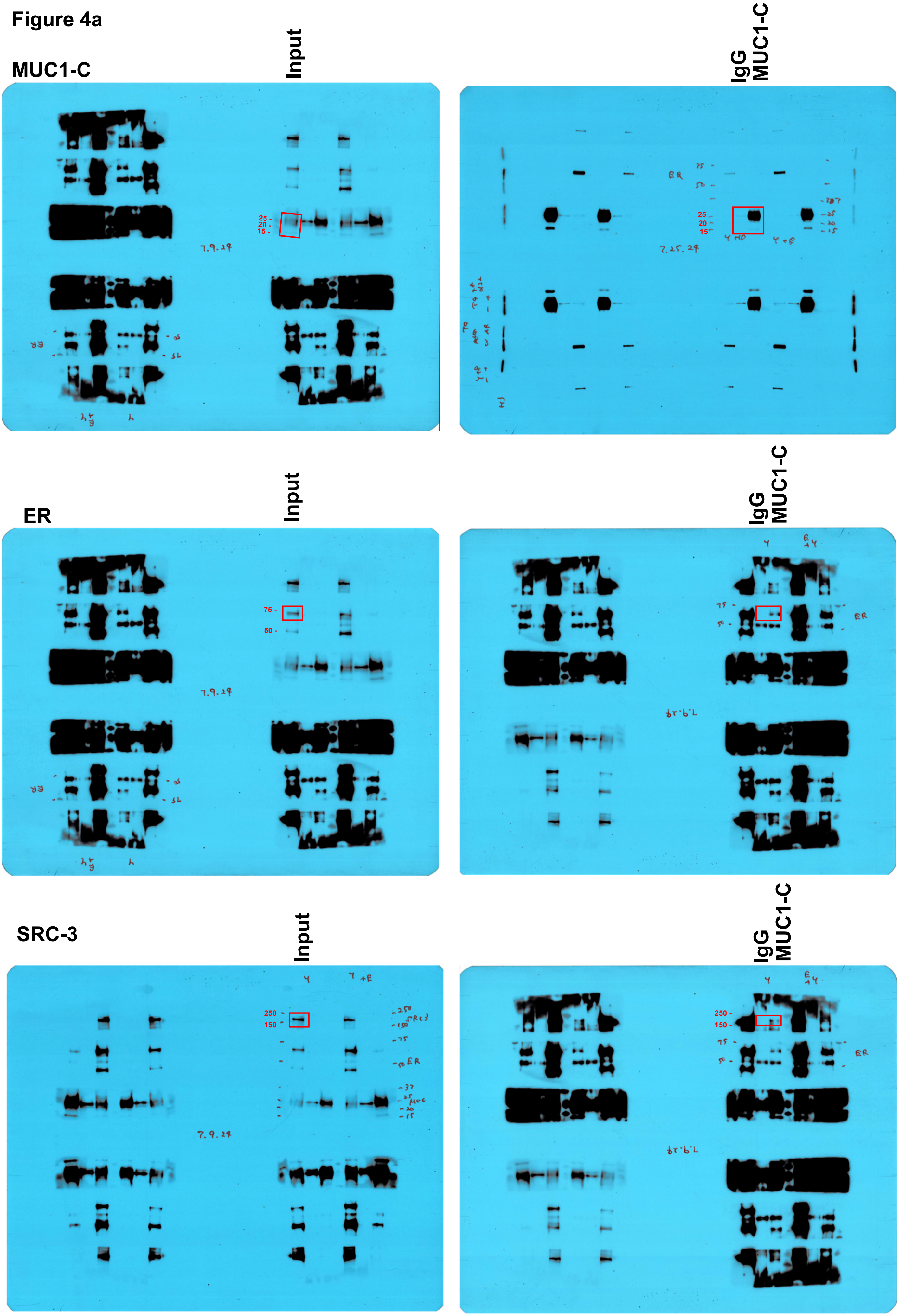
MUC1-C
Input
IgG
MUC1-C
25
20
15
ER
Input
IgG
MUC1-C
75
50
SRC-3
Input
IgG
MUC1-C
250
150

Figure 4a (continued)

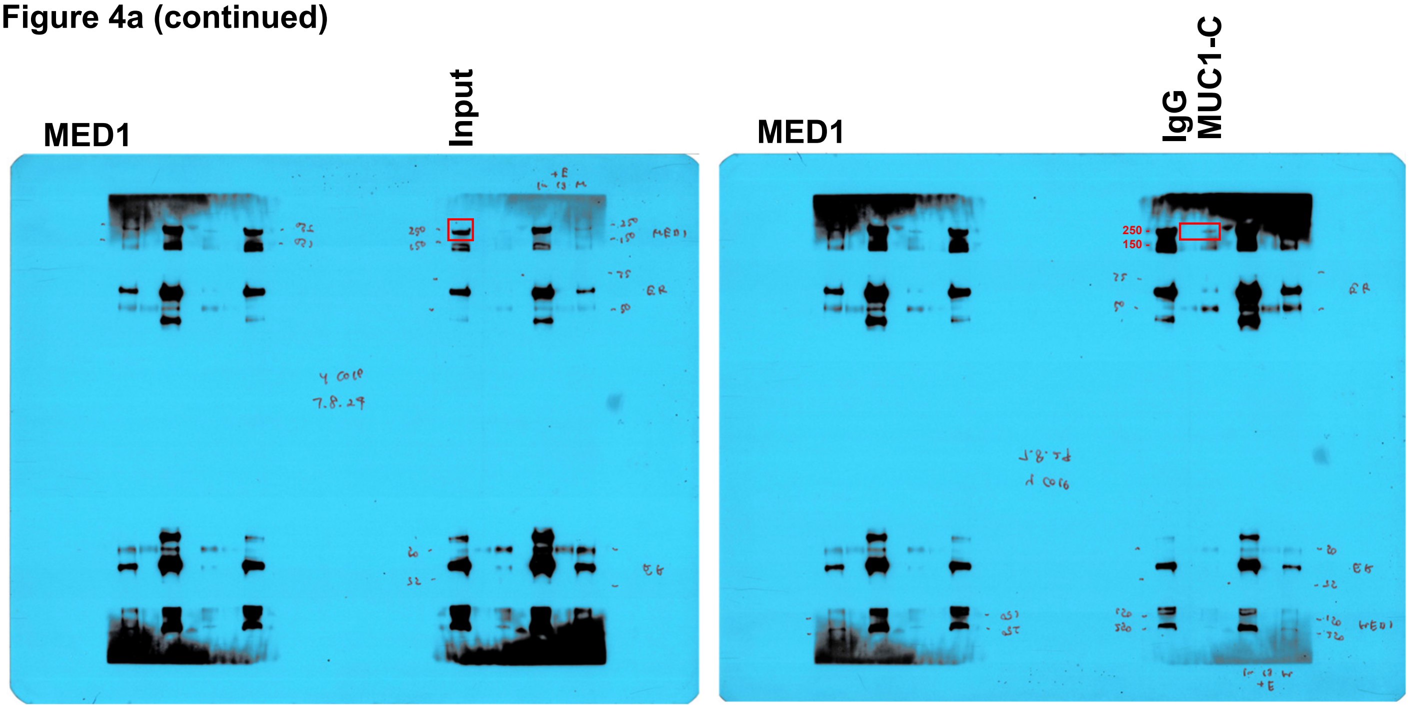

Figure 4b

MUC1-C

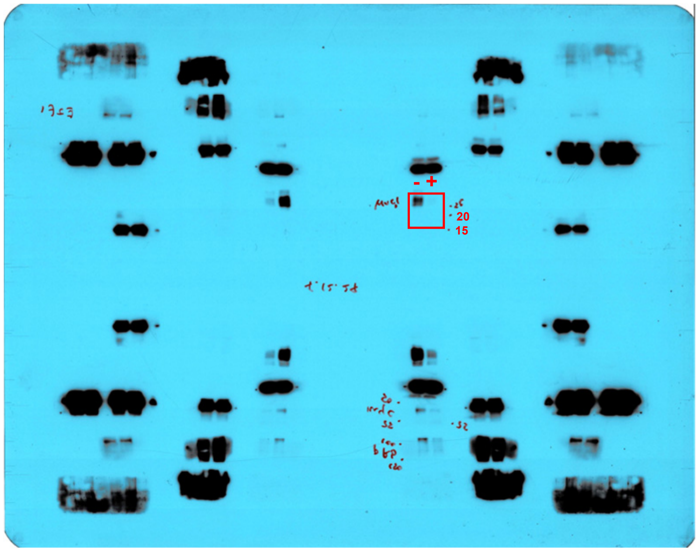

MED1

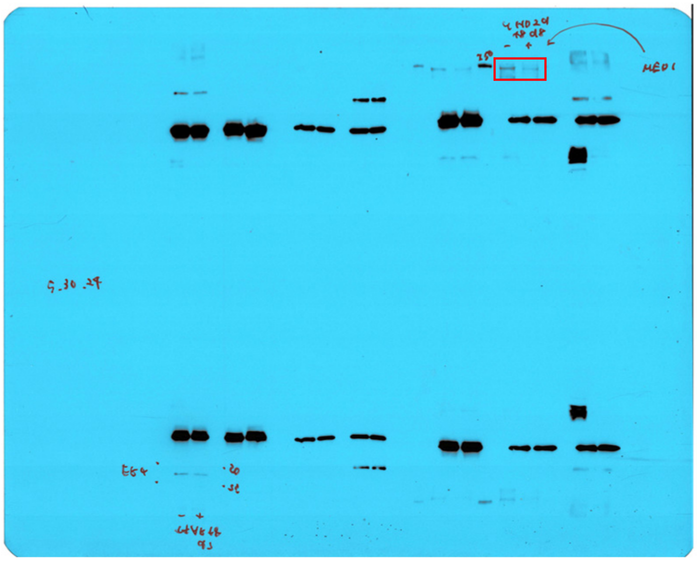

ER

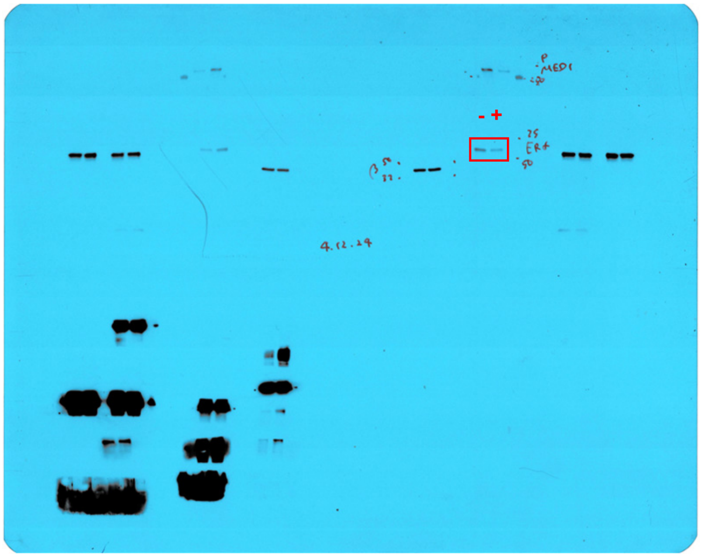

β-actin

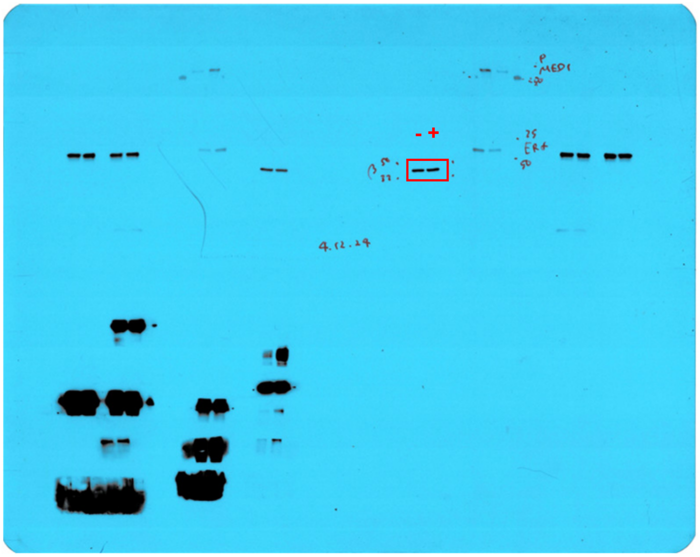

SRC-3

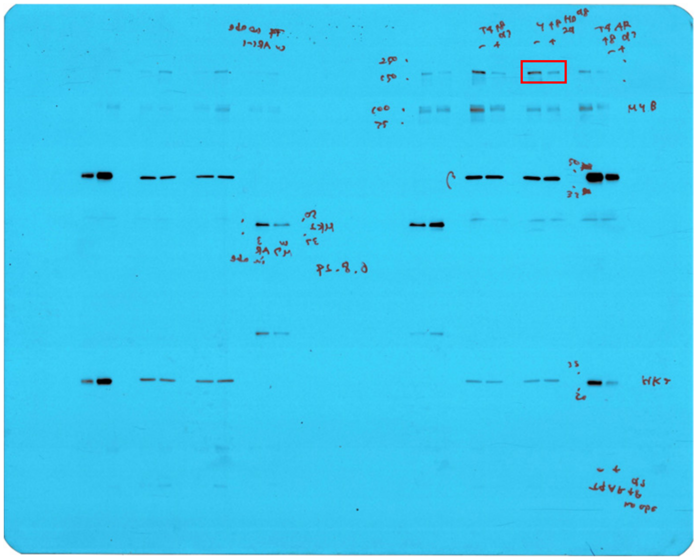

Figure 4c

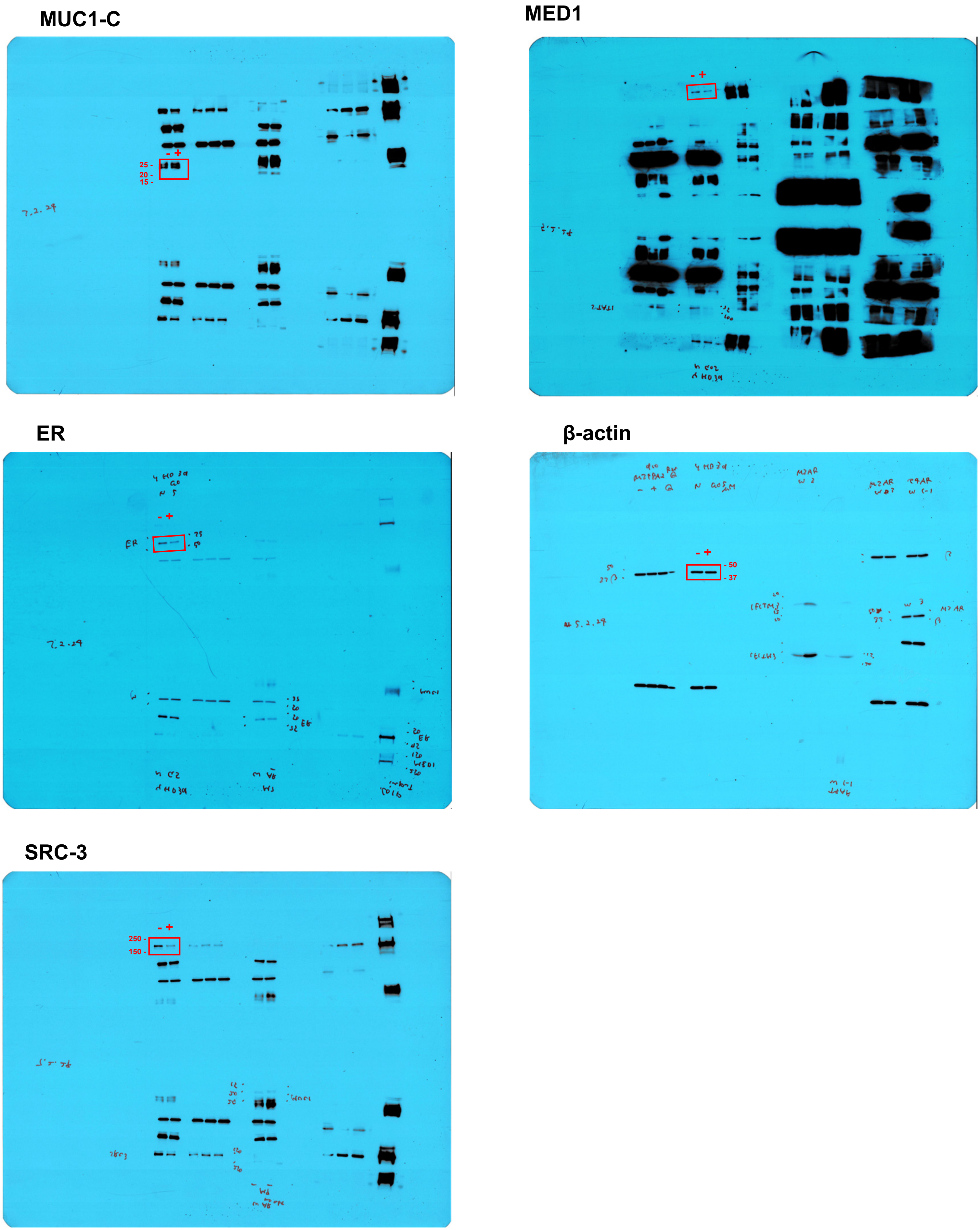

Figure 4d

MUC1-C

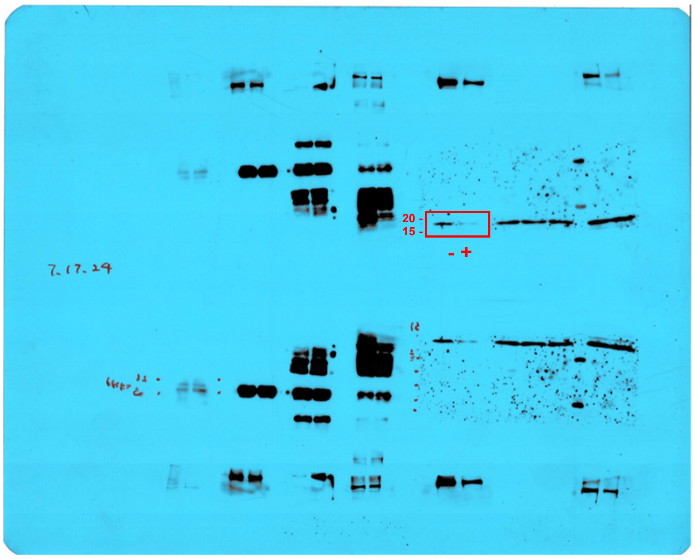

MED1

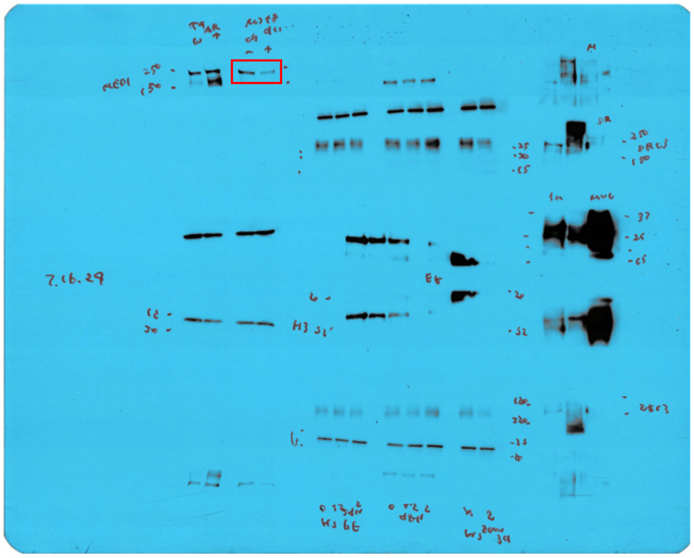

ER

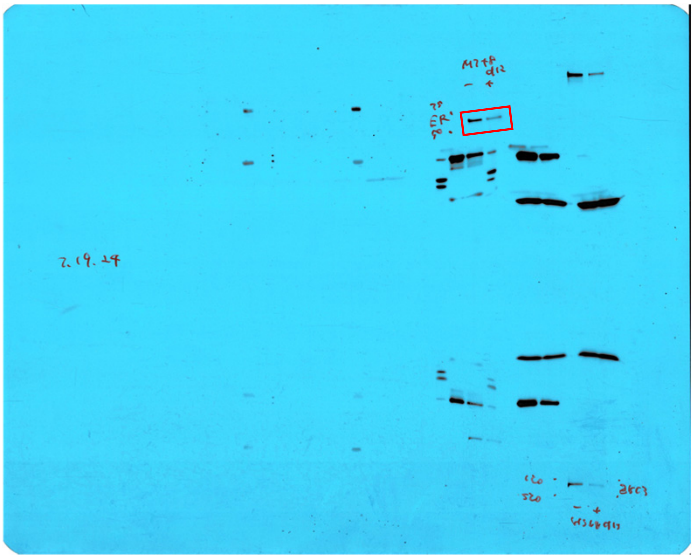

H3

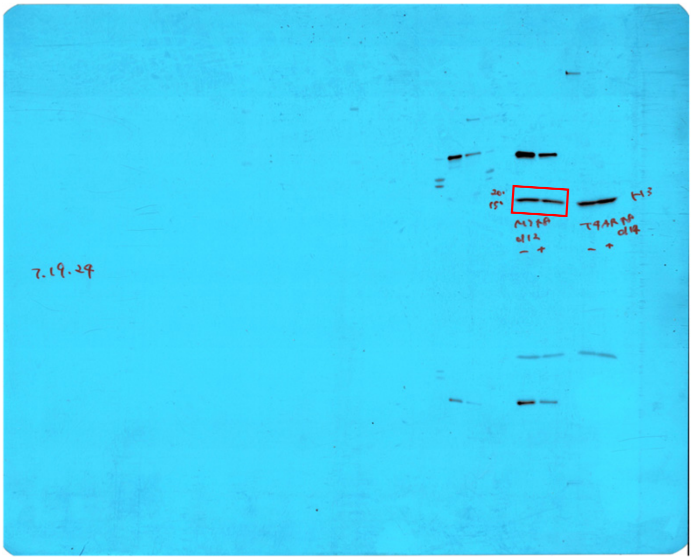

SRC-3

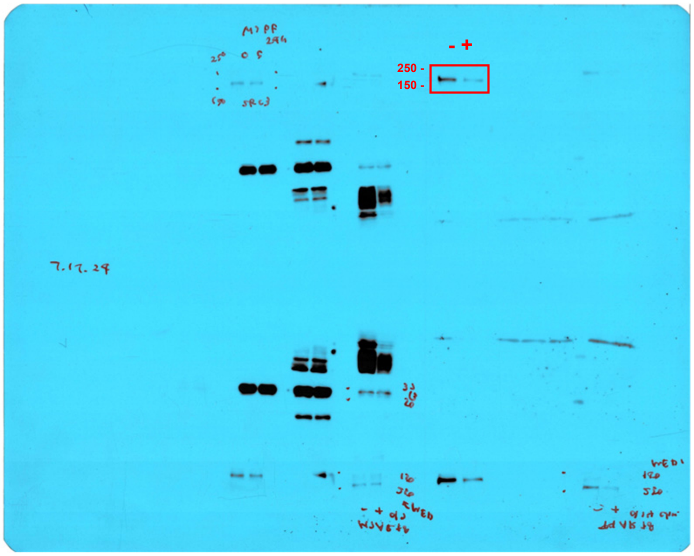

Figure 4e

MUC1-C

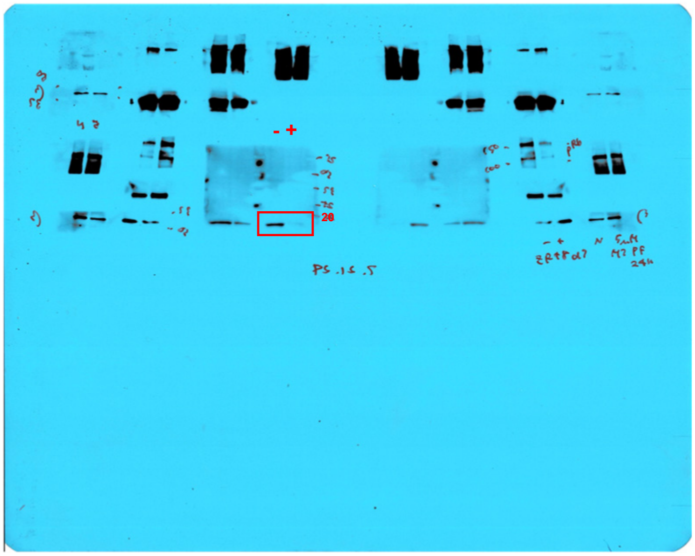

MED1

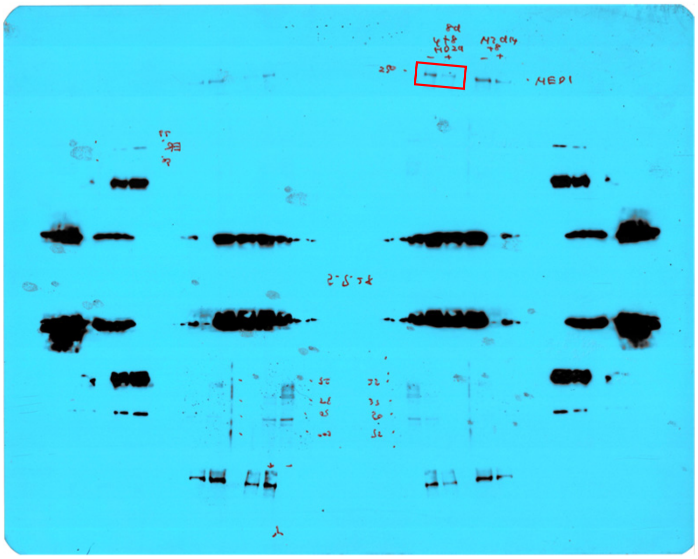

ER

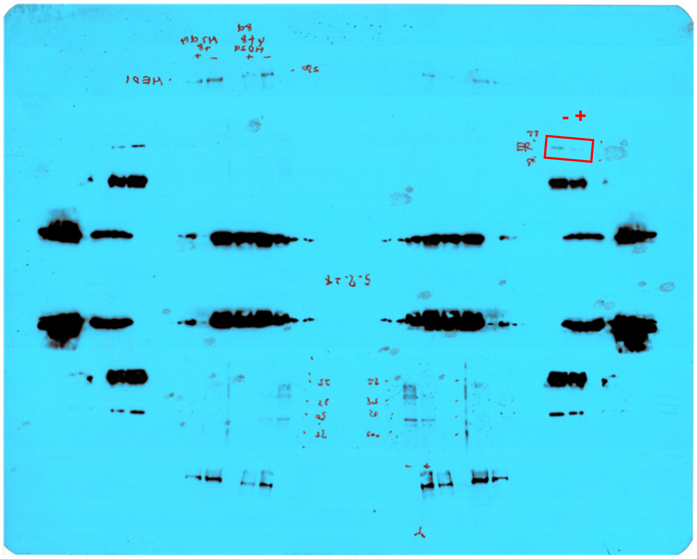

H3

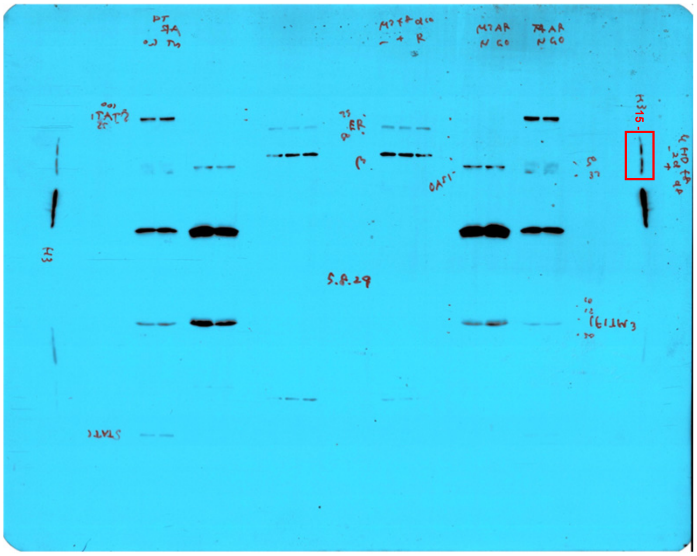

SRC-3

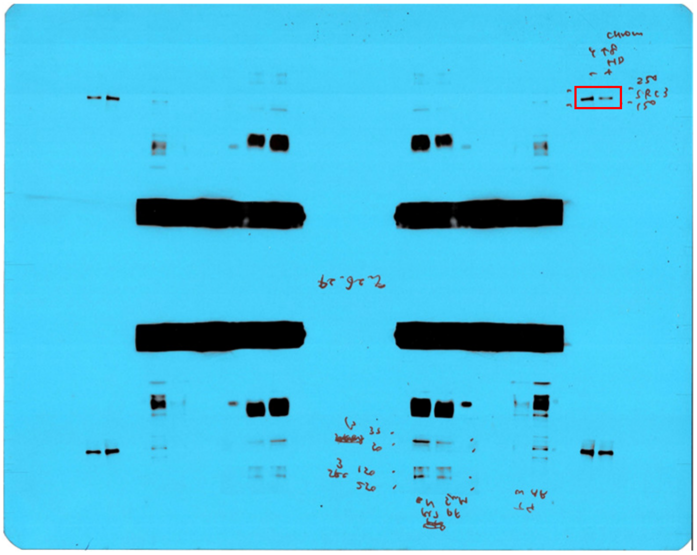

Figure 5a

pRB (S780) **MCF-7**

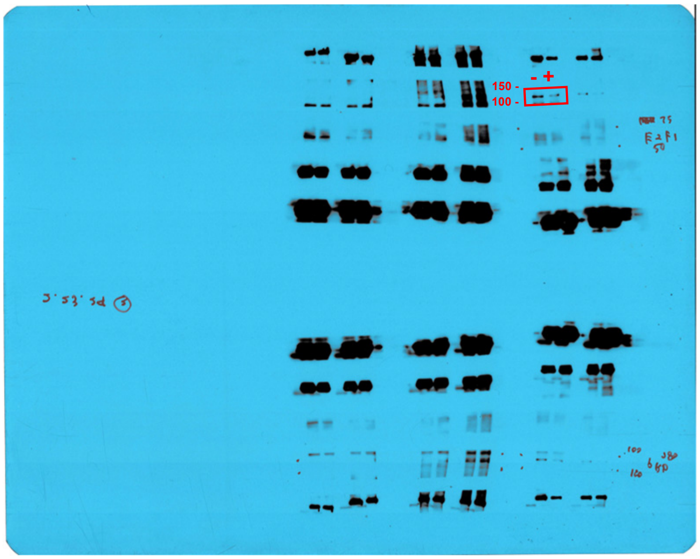

pRB (S780) **T47D**

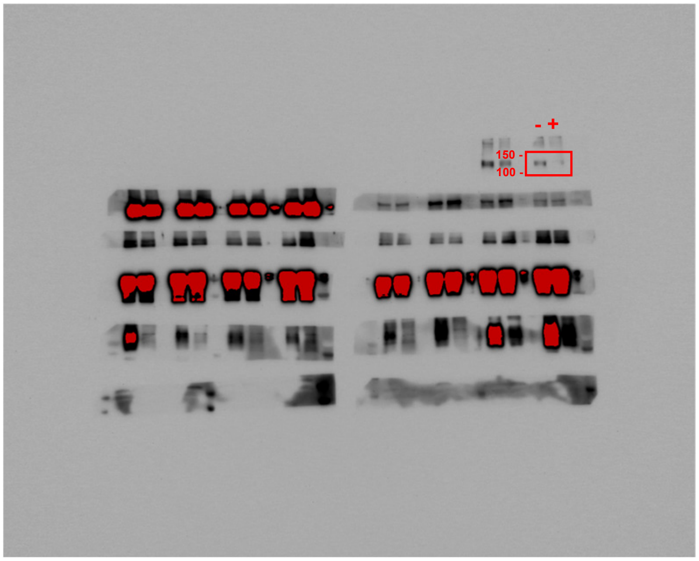

BIORAD ChemiDoc MP developer was used for this blot.
A classical film developer was used for all other blots.

RB **MCF-7**

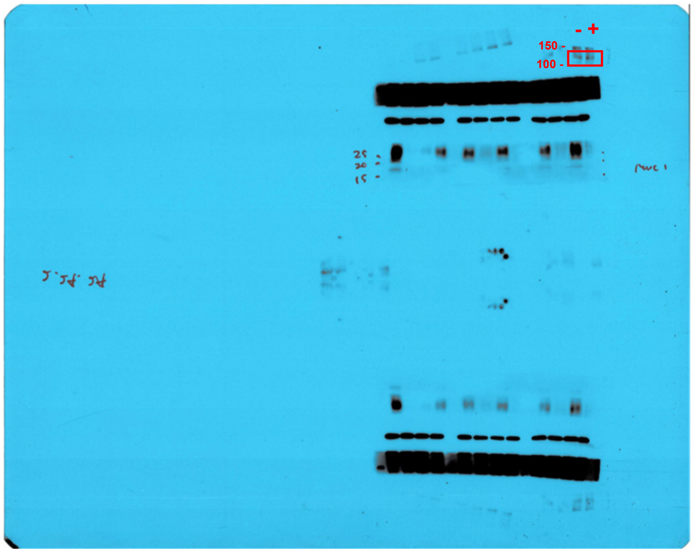

RB **T47D**

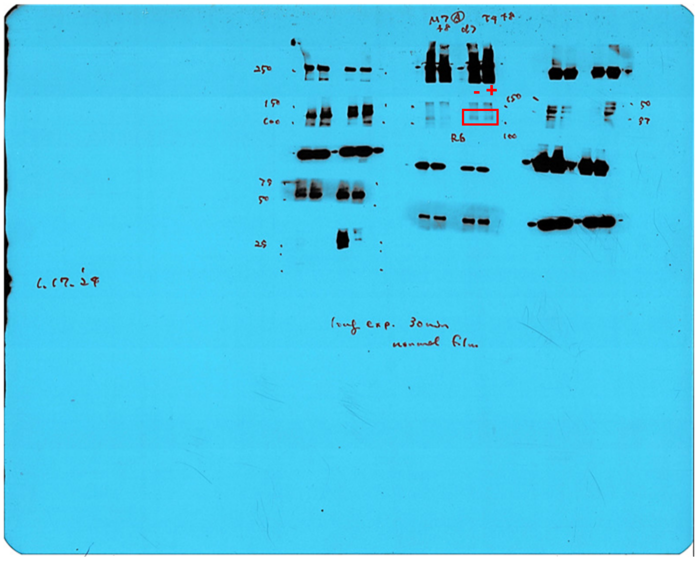

E2F1 **MCF-7**

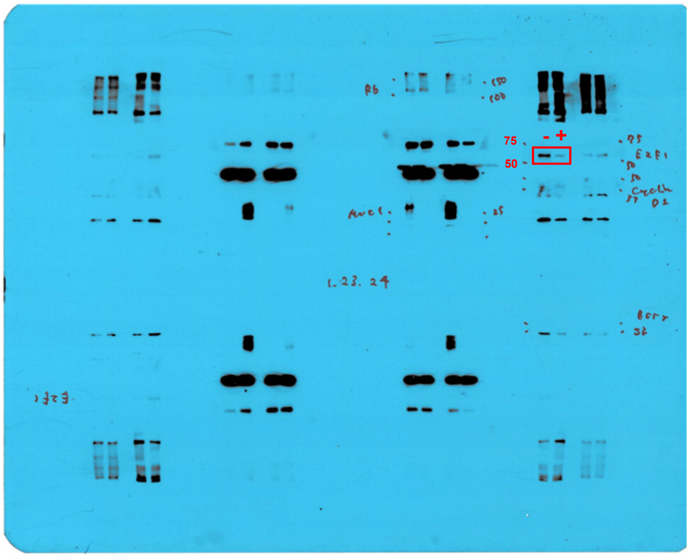

E2F1 **T47D**

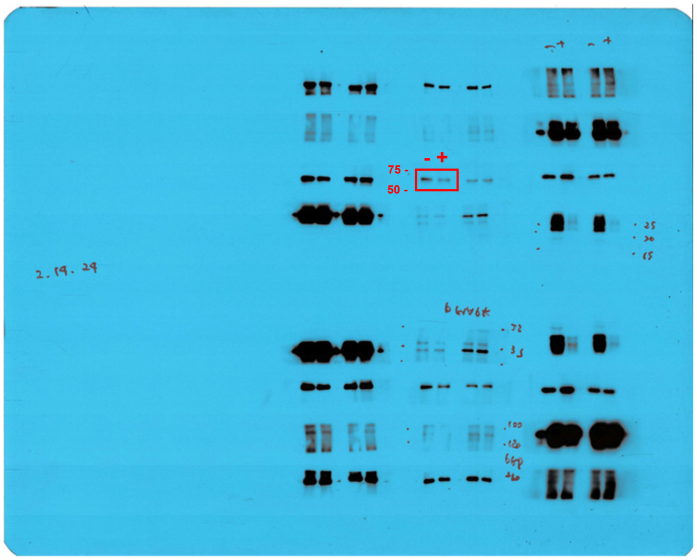

Figure 5a (continued)

β-actin **MCF-7**

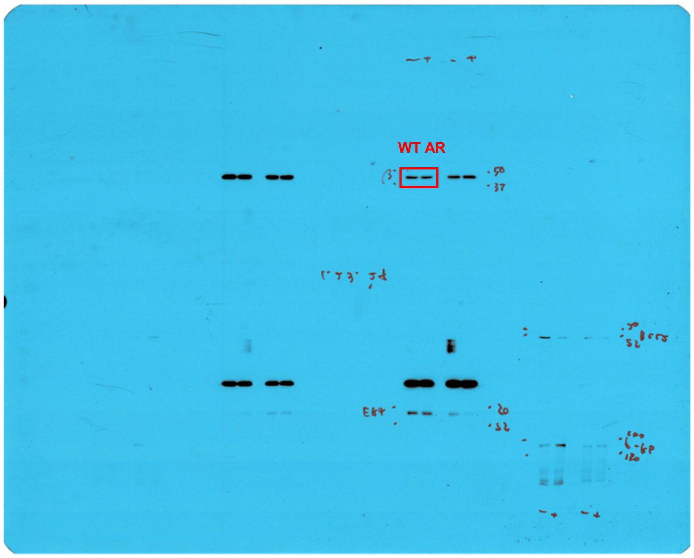

β-actin **T47D**

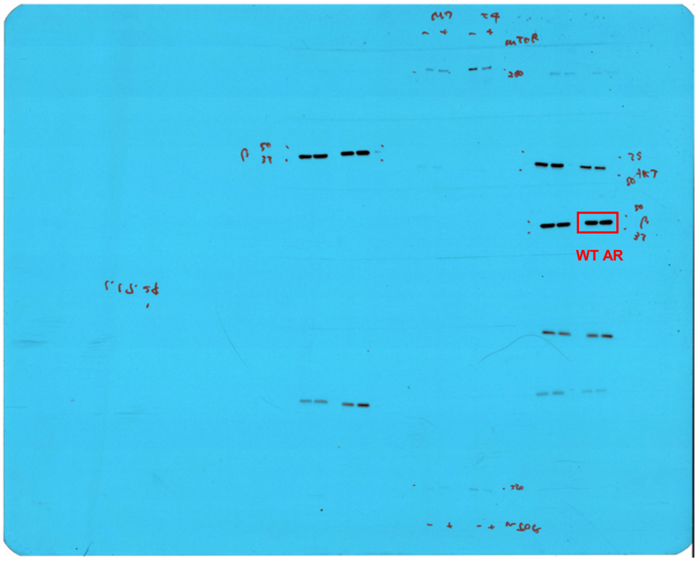

Figure 5d

MUC1-C **MCF-7**

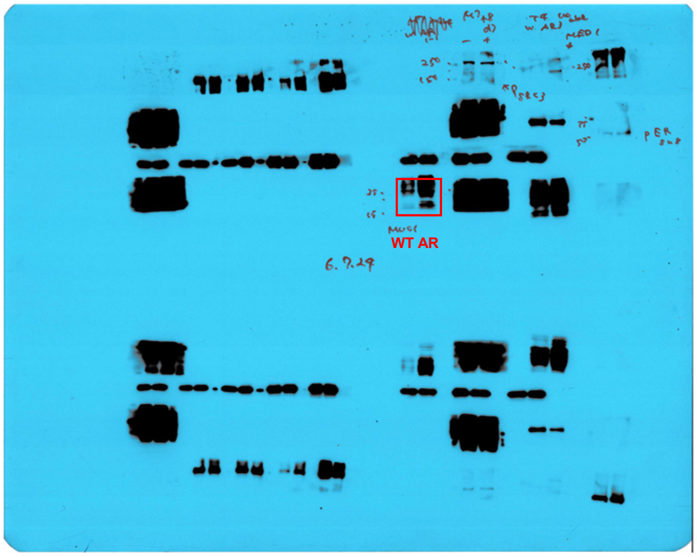

MUC1-C **T47D**

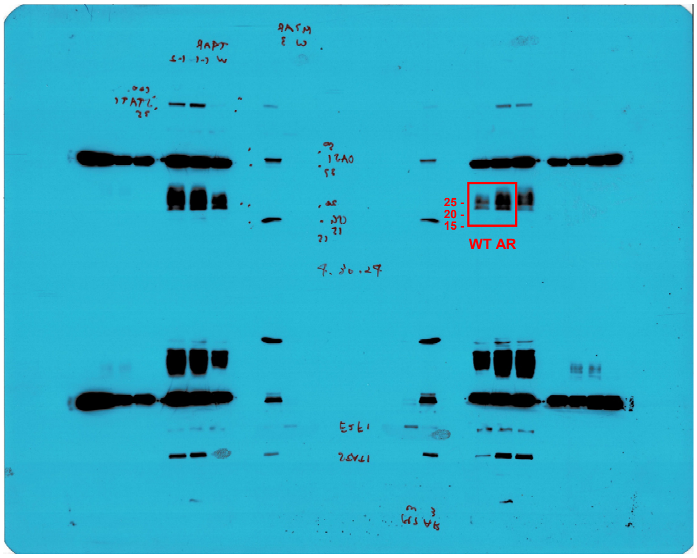

β-actin **MCF-7**

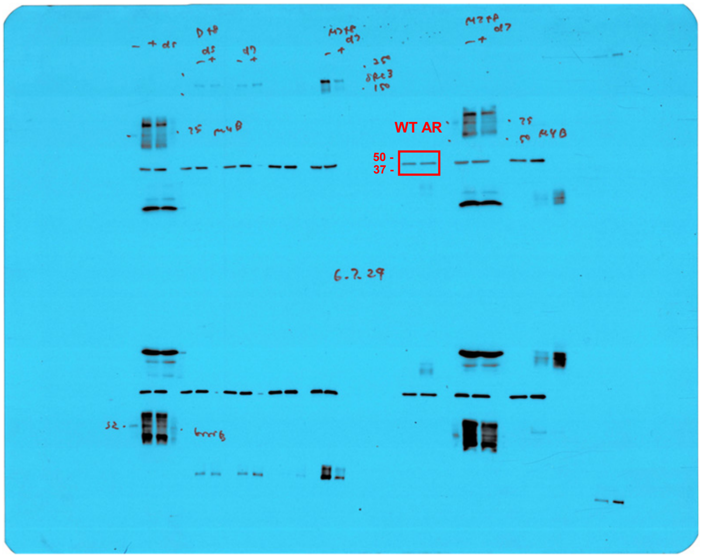

β-actin **T47D**

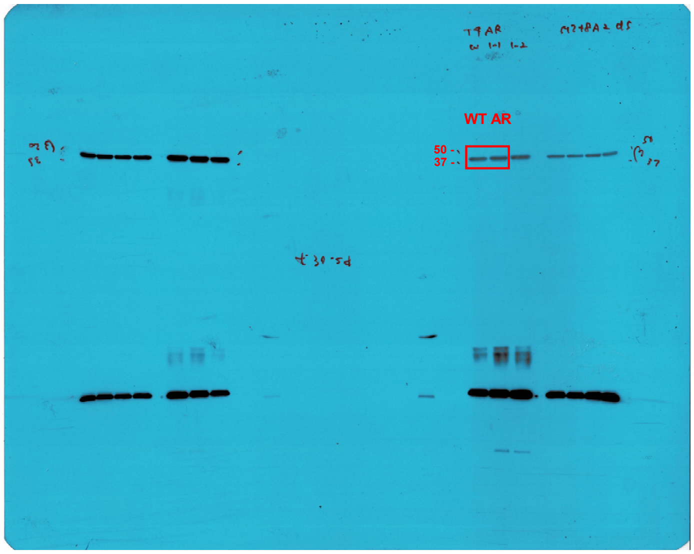

Figure 5e

MUC1-C MCF-7

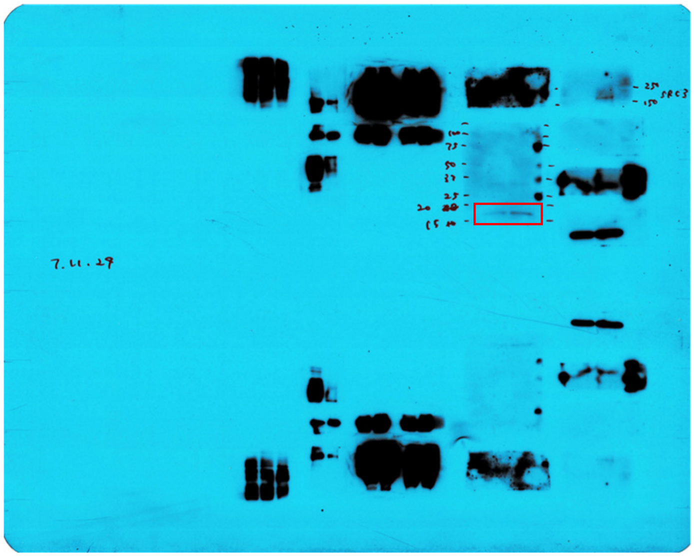

MUC1-C T47D

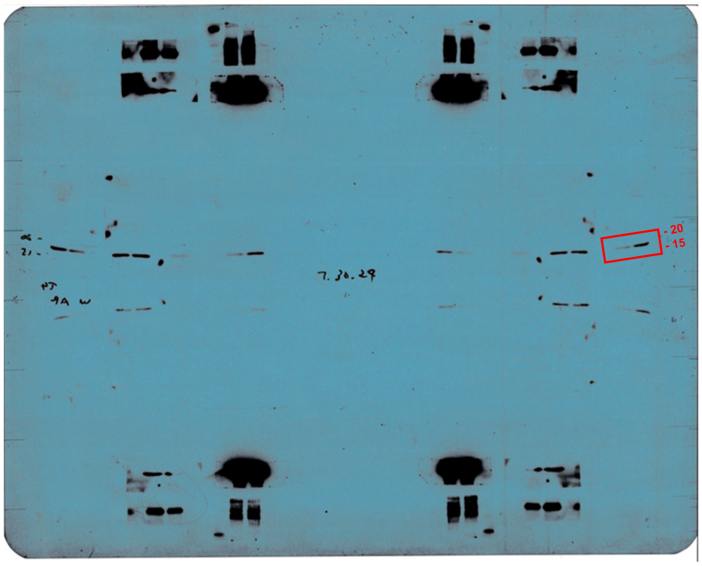

MED1 MCF-7

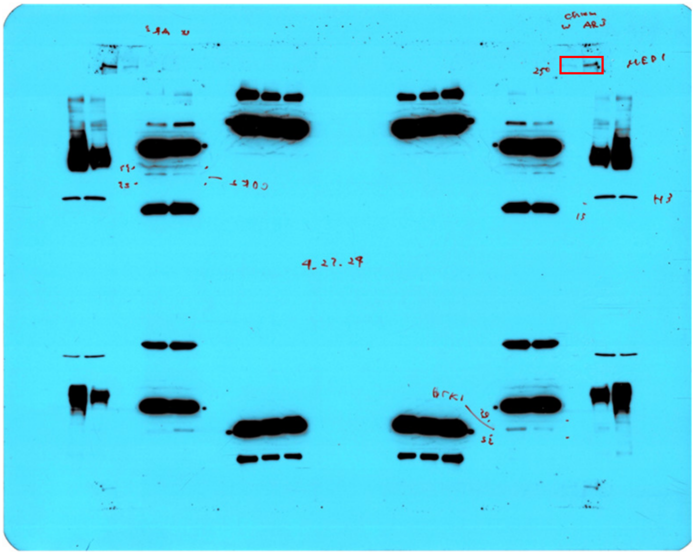

MED1 T47D

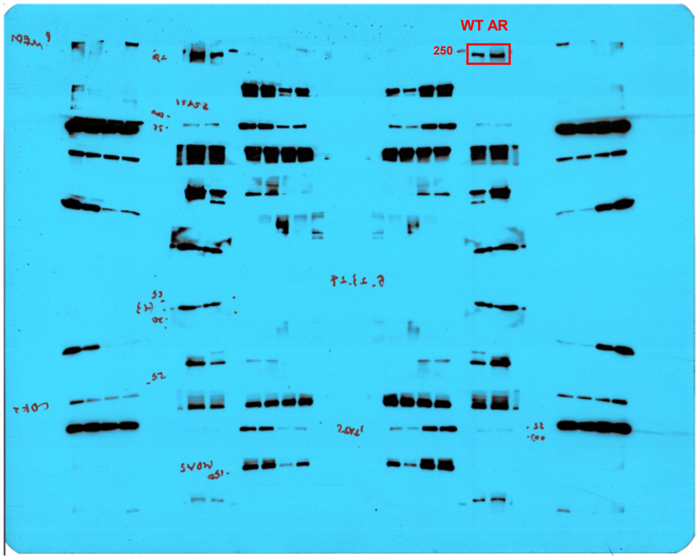

pCDK7(T170) MCF-7

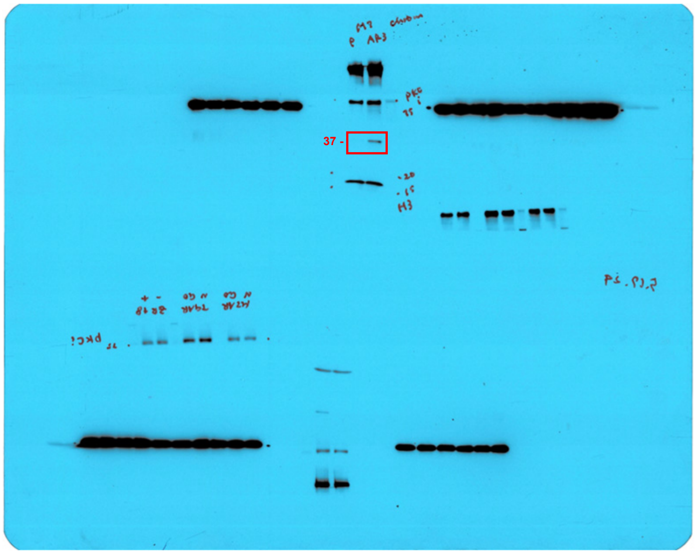

pCDK7(T170) T47D

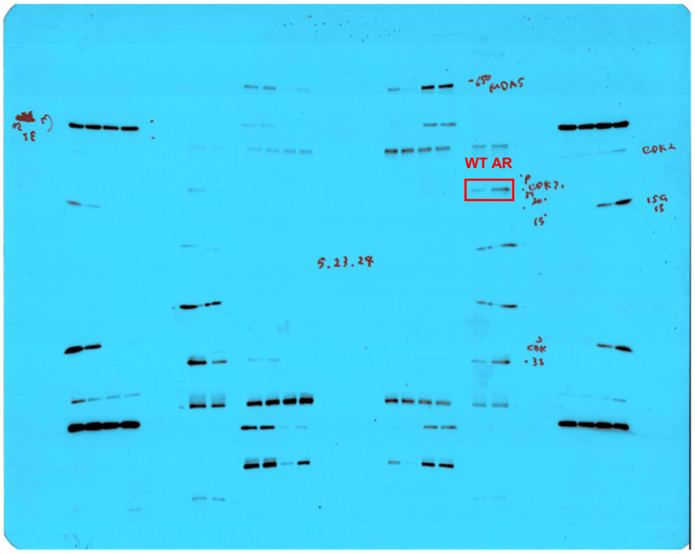

Figure 5e (continued)

CDK7 MCF-7

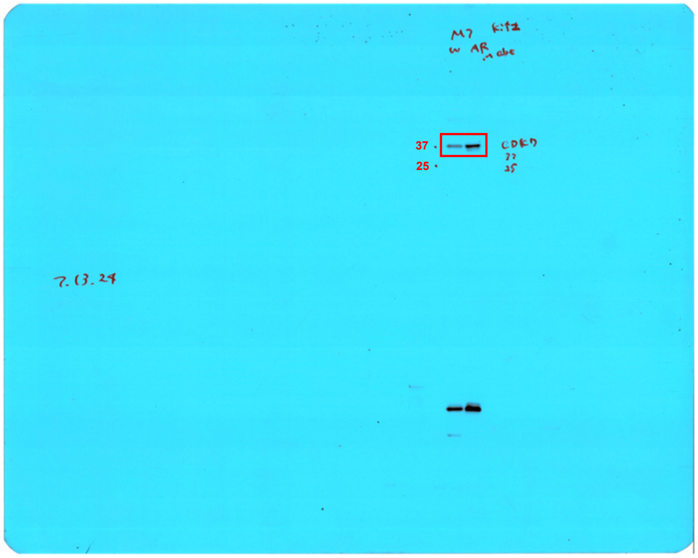

CDK7 T47D

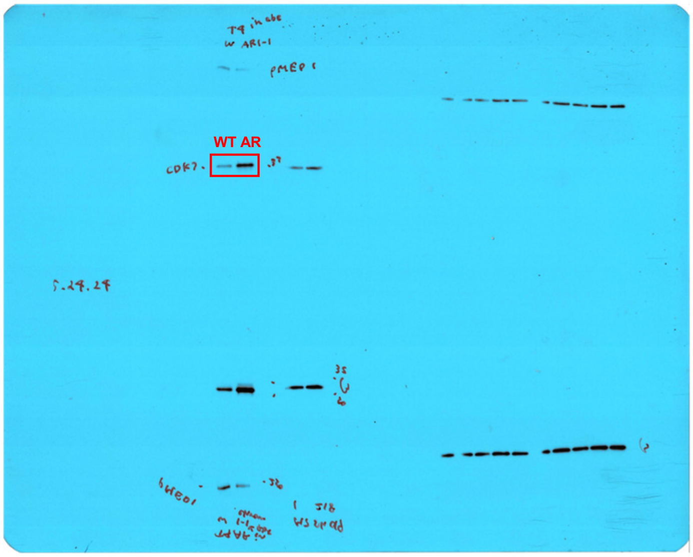

SRC-3 T47D MCF-7

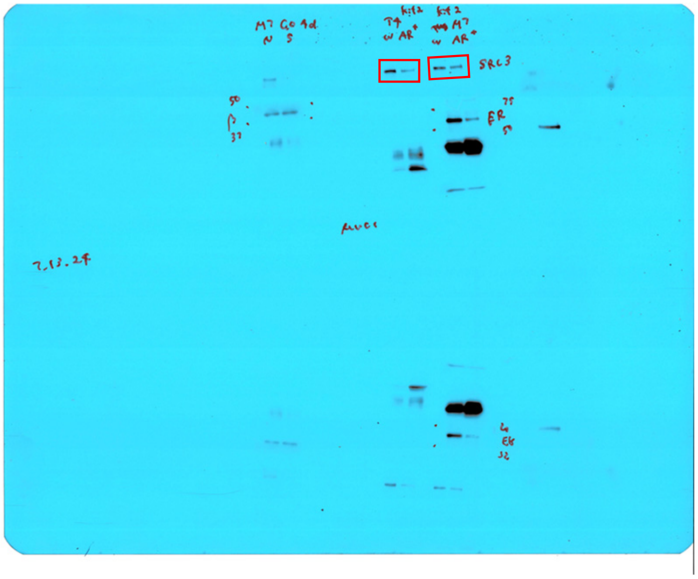

MYC MCF-7

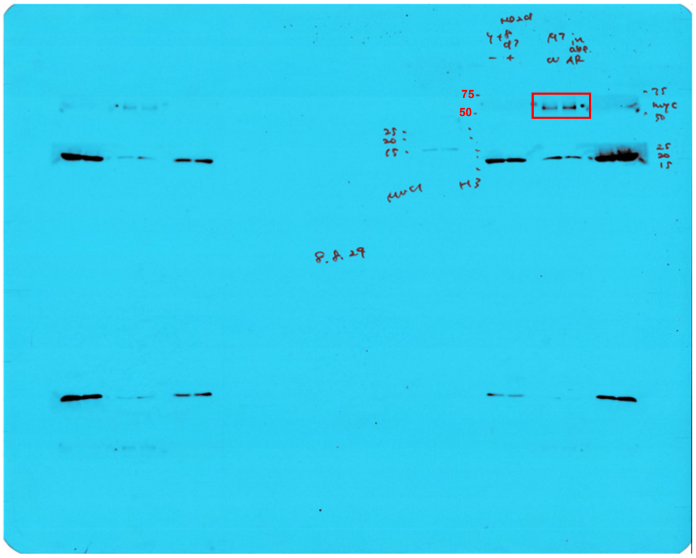

MYC T47D

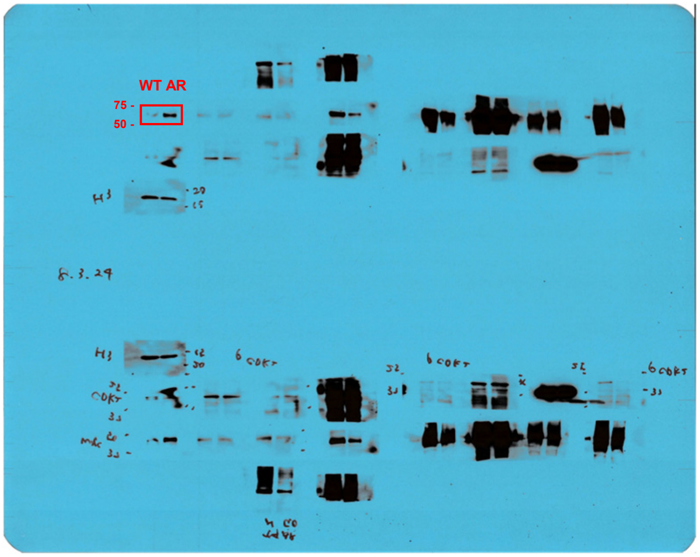

Figure 5e (continued)

H3 **MCF-7**

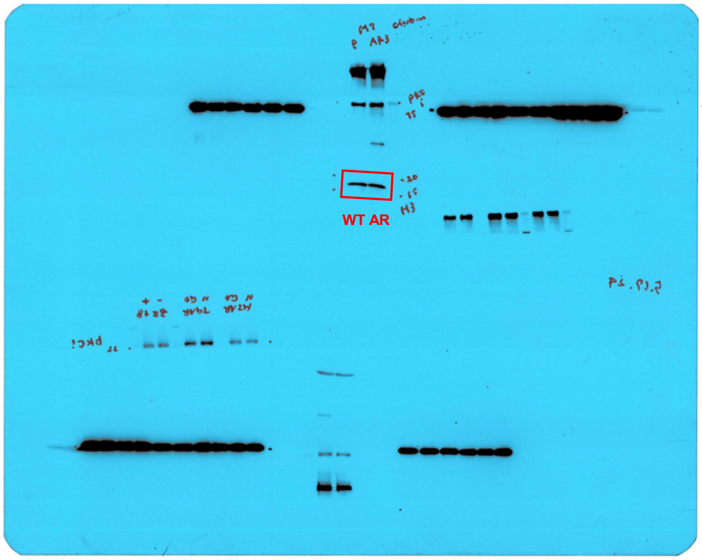

H3 **T47D**

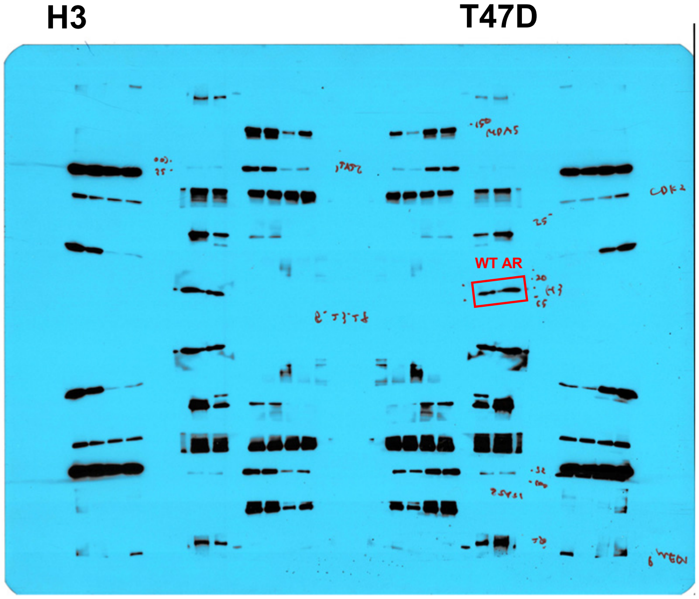

Figure 5f

MUC1-C

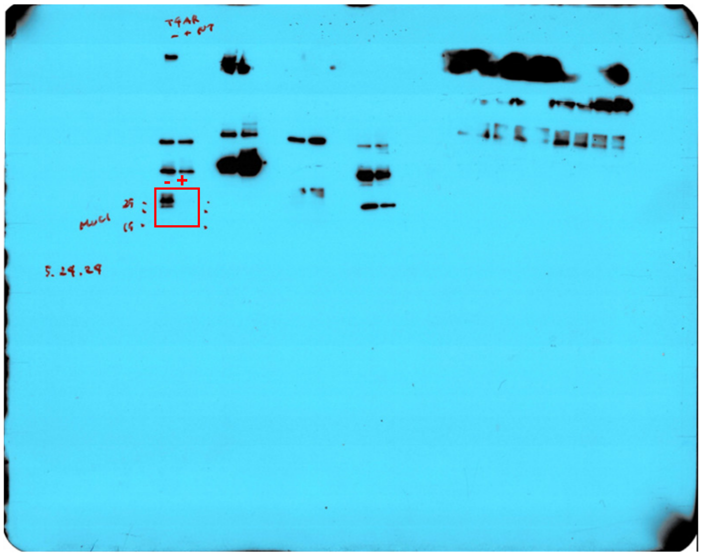

E2F1

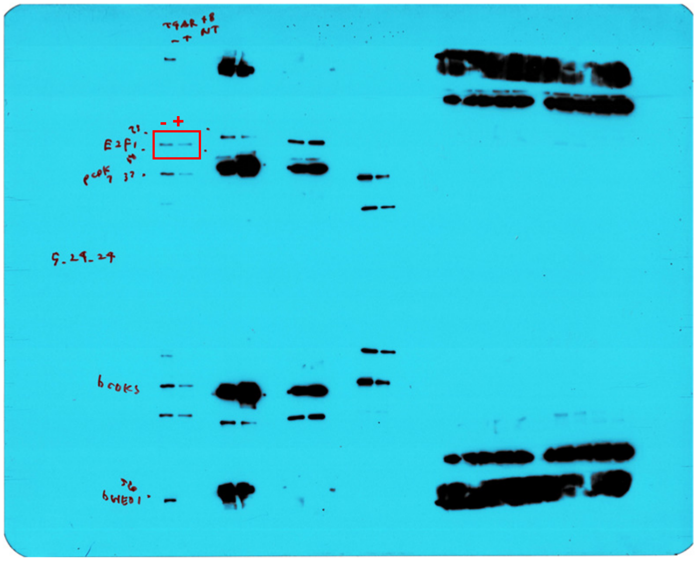

pRB (S780)

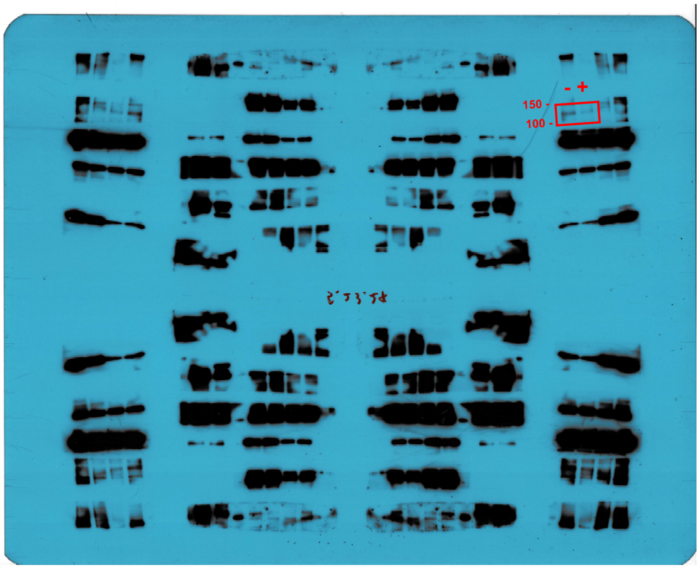

ER

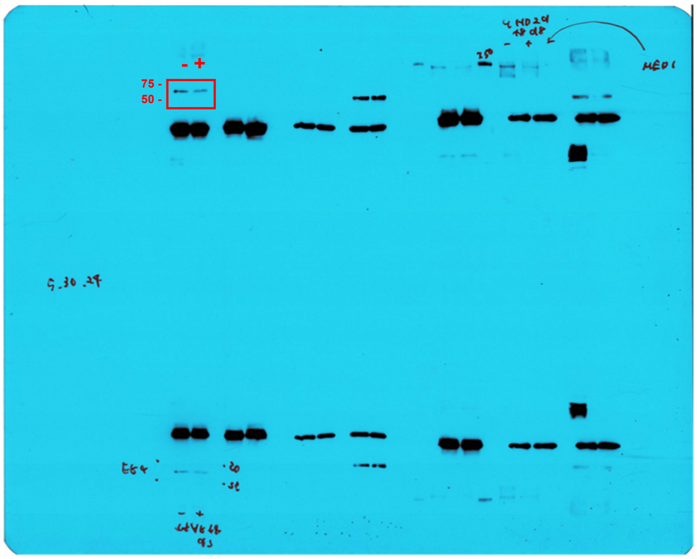

RB

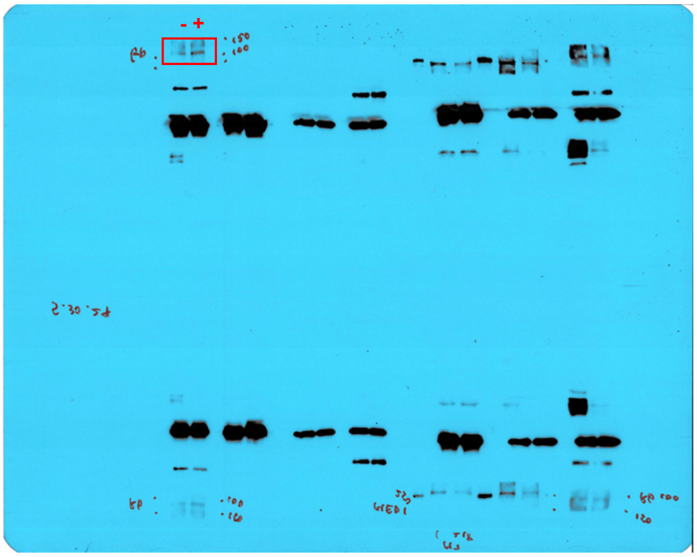

MK2

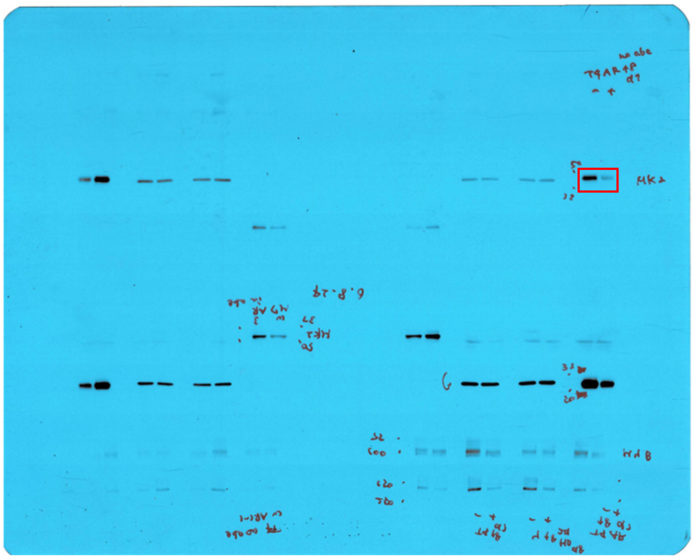

Figure 5f (continued)

SRC-3

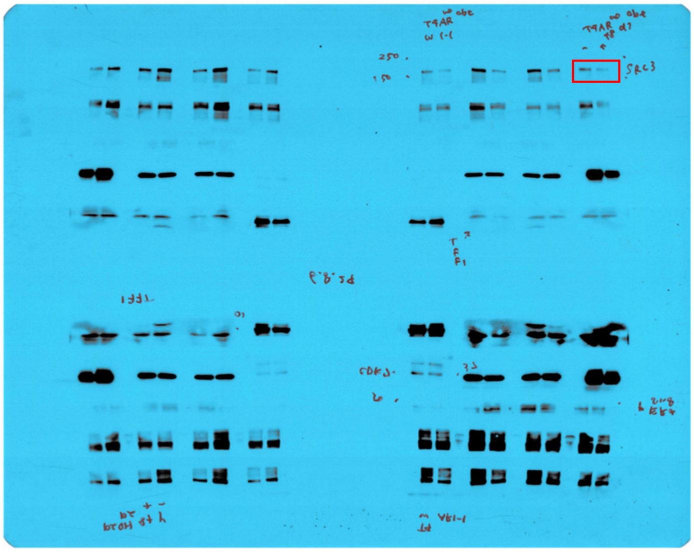

MED1

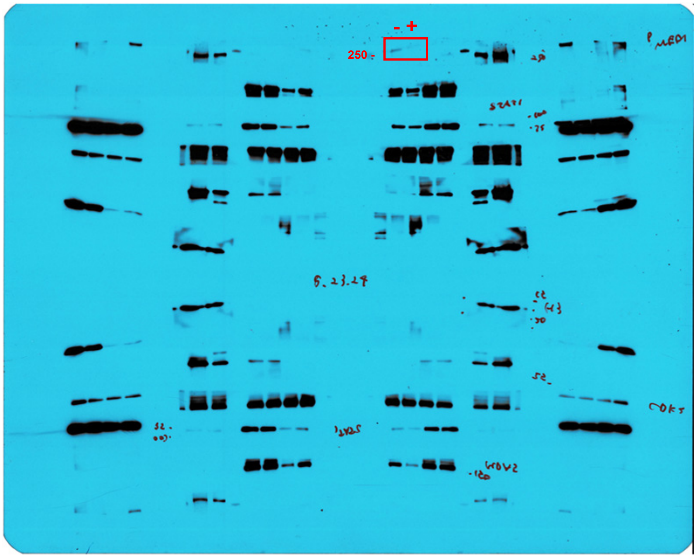

pCDK7 (T170)

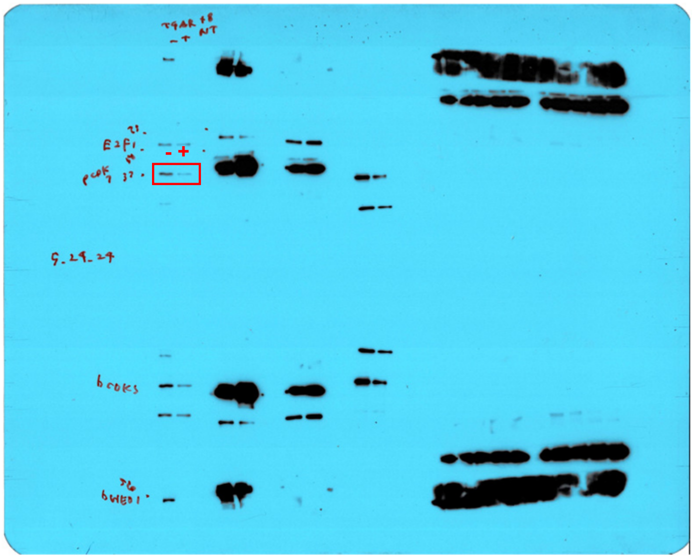

β-actin

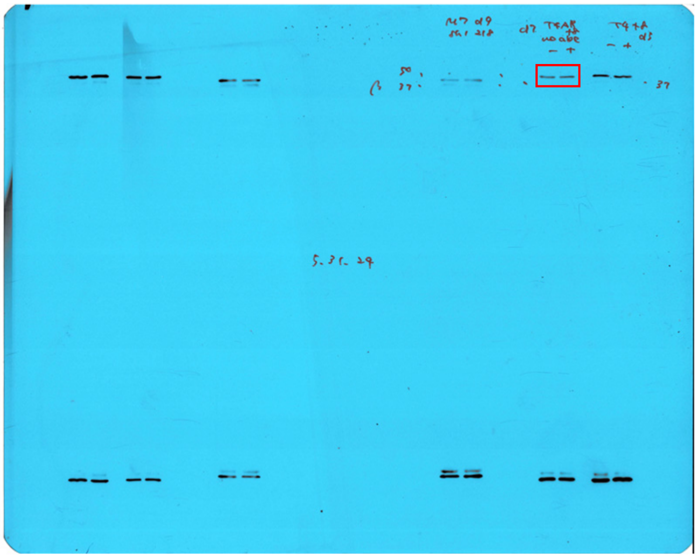

CDK7

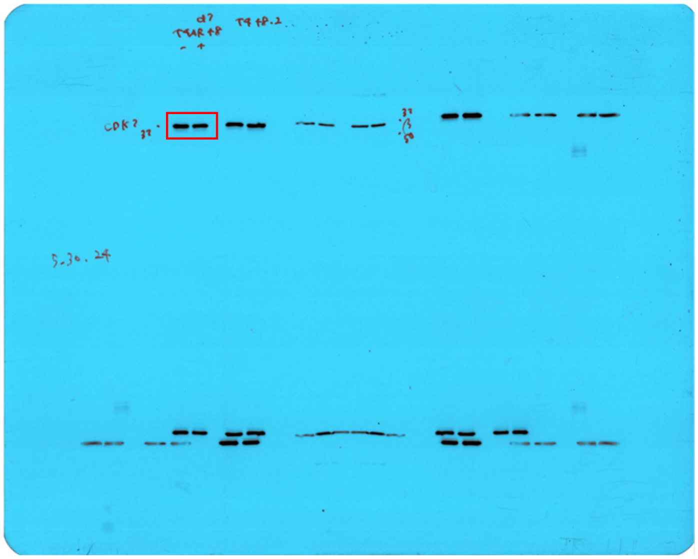

Figure 5g

MUC1-C

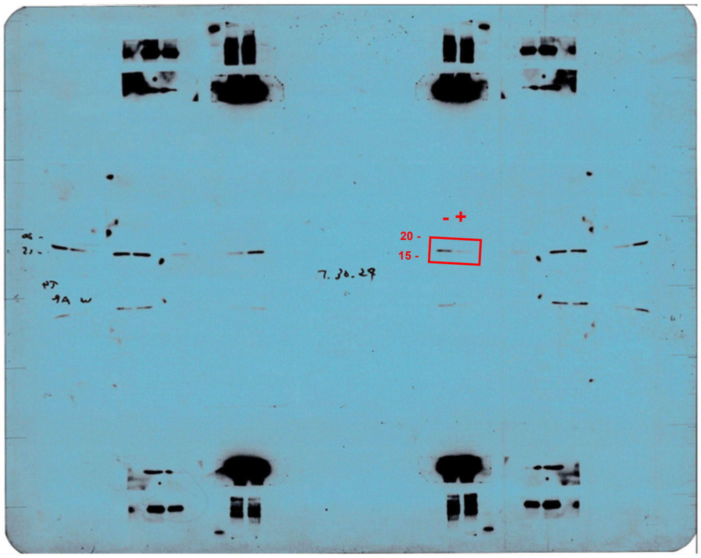

MED1

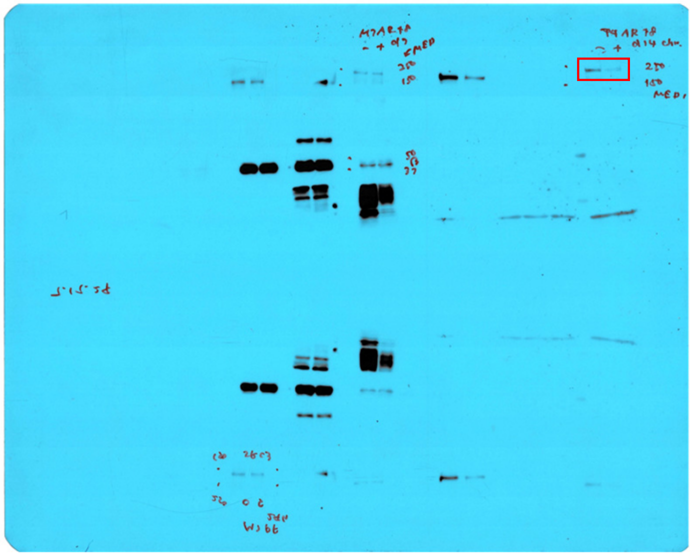

ER SRC-3

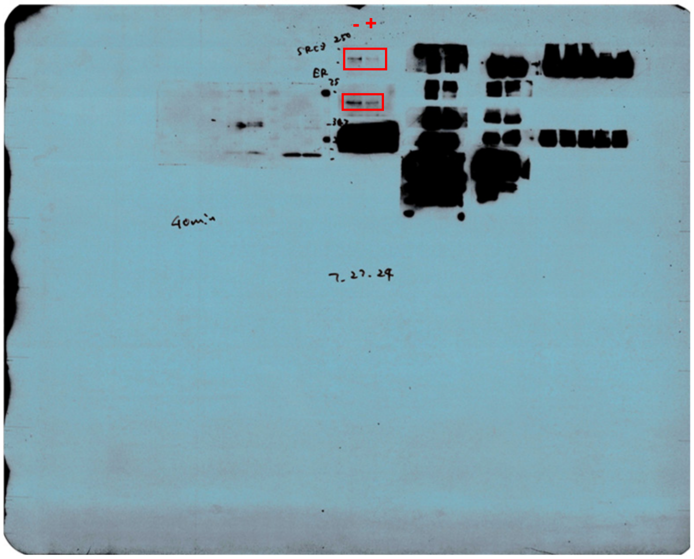

MYC

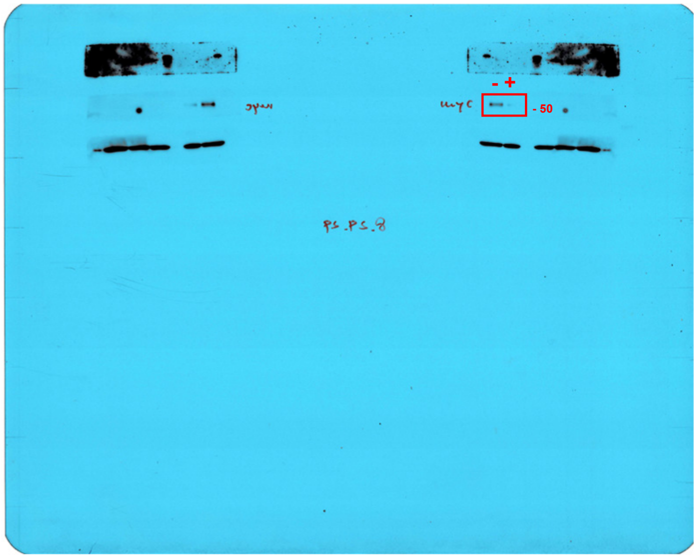

H3

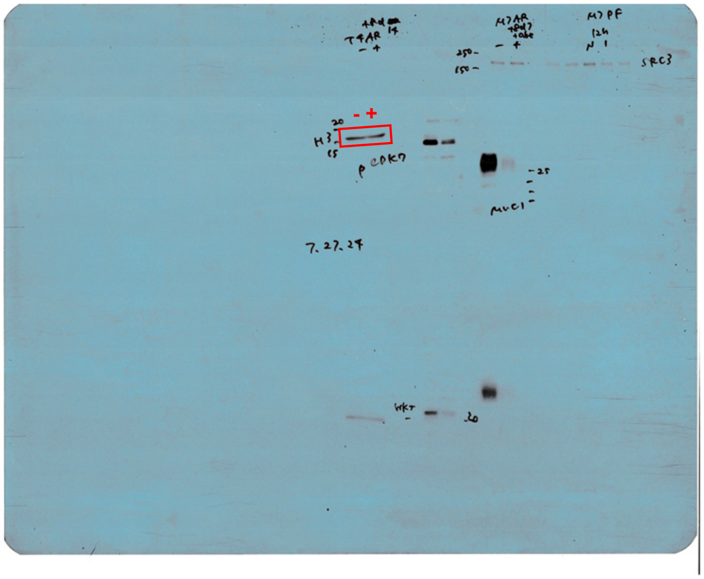

Supplemental Figure S8

Cropped images of western blots

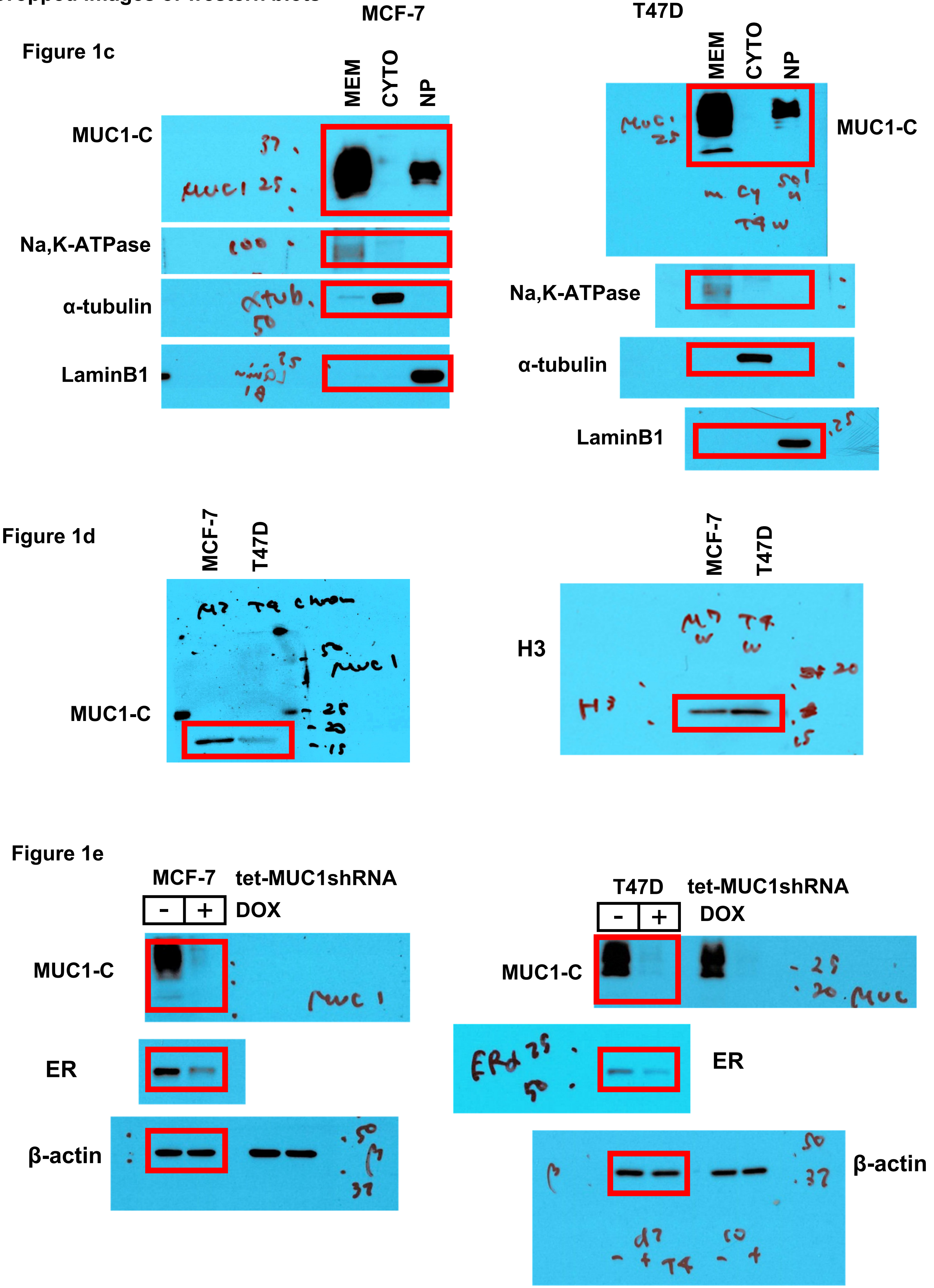

Figure 2a

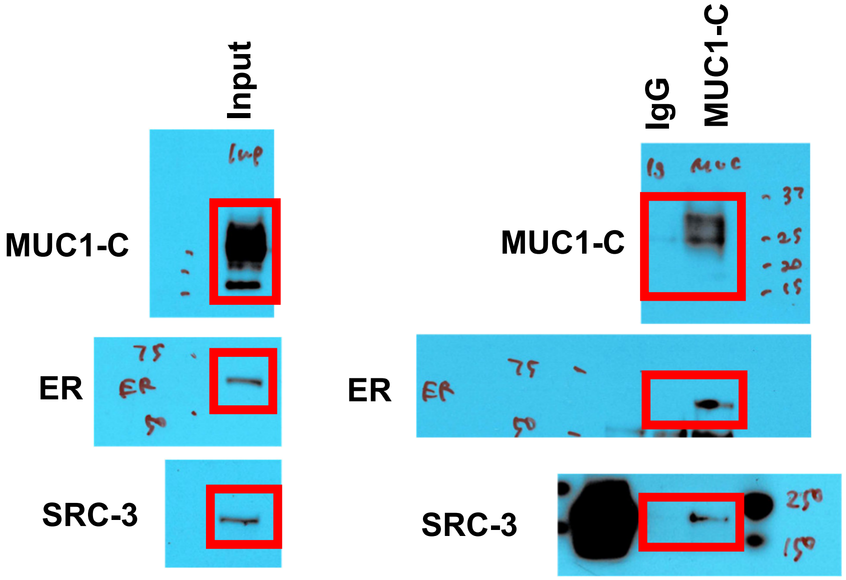

Figure 2b

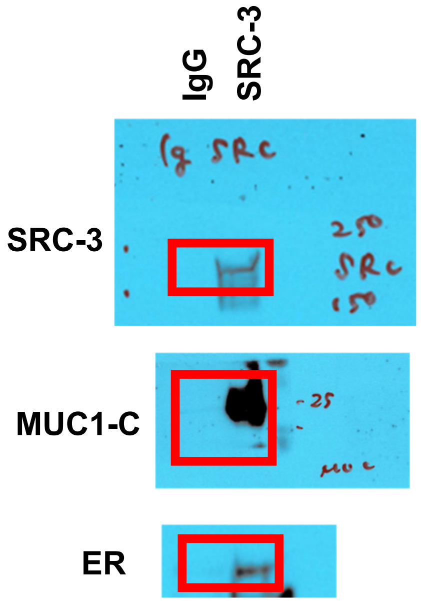

Figure 2c

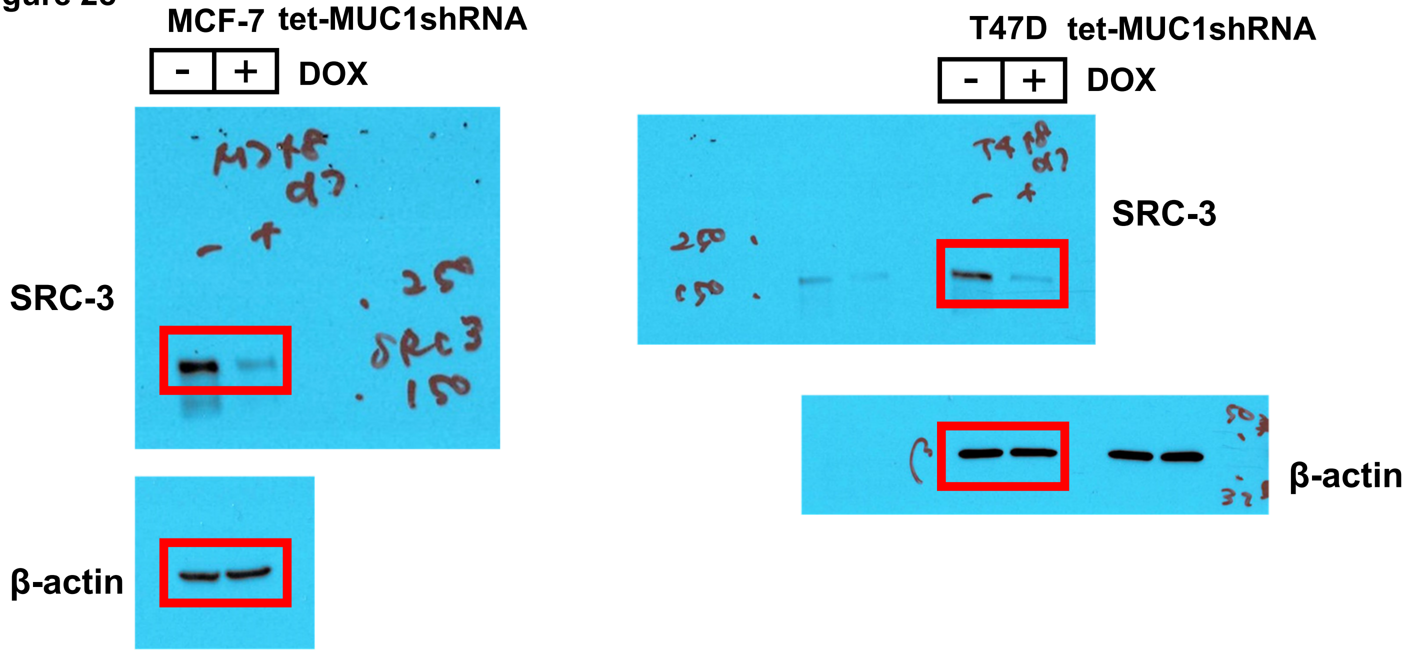

Figure 2d

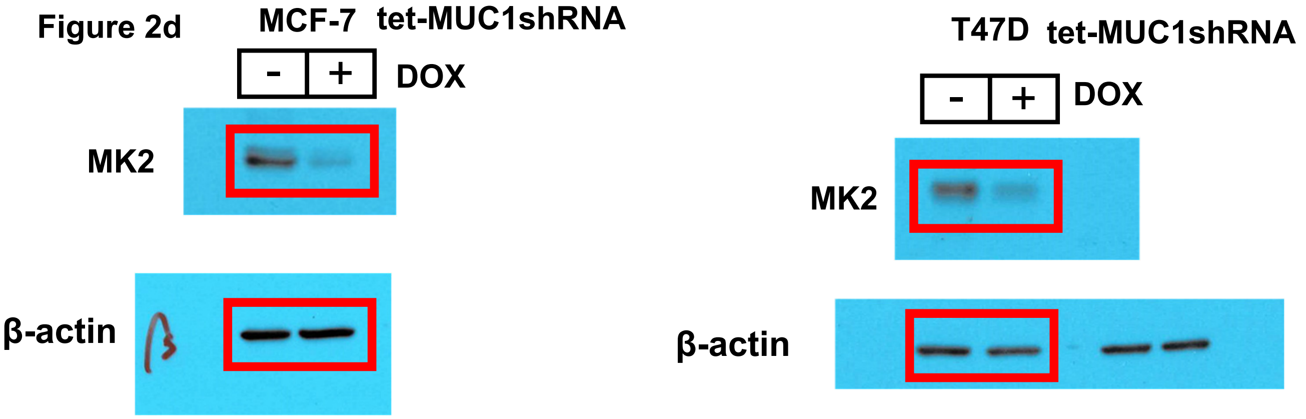

Figure 3a

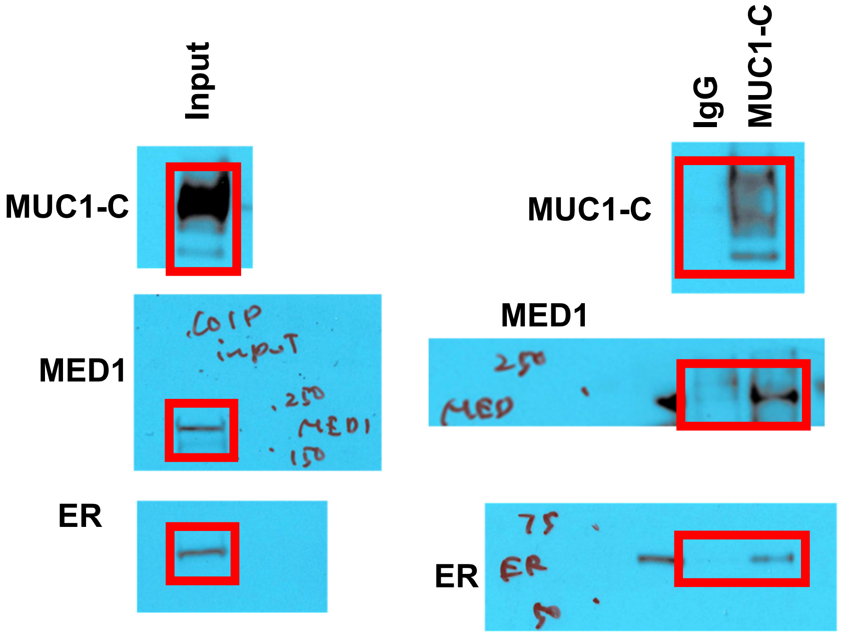

Figure 3b

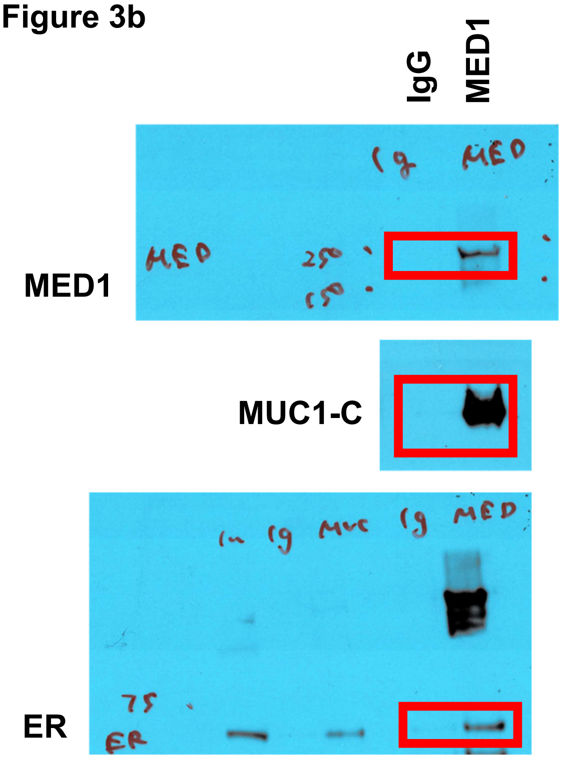

Figure 3c

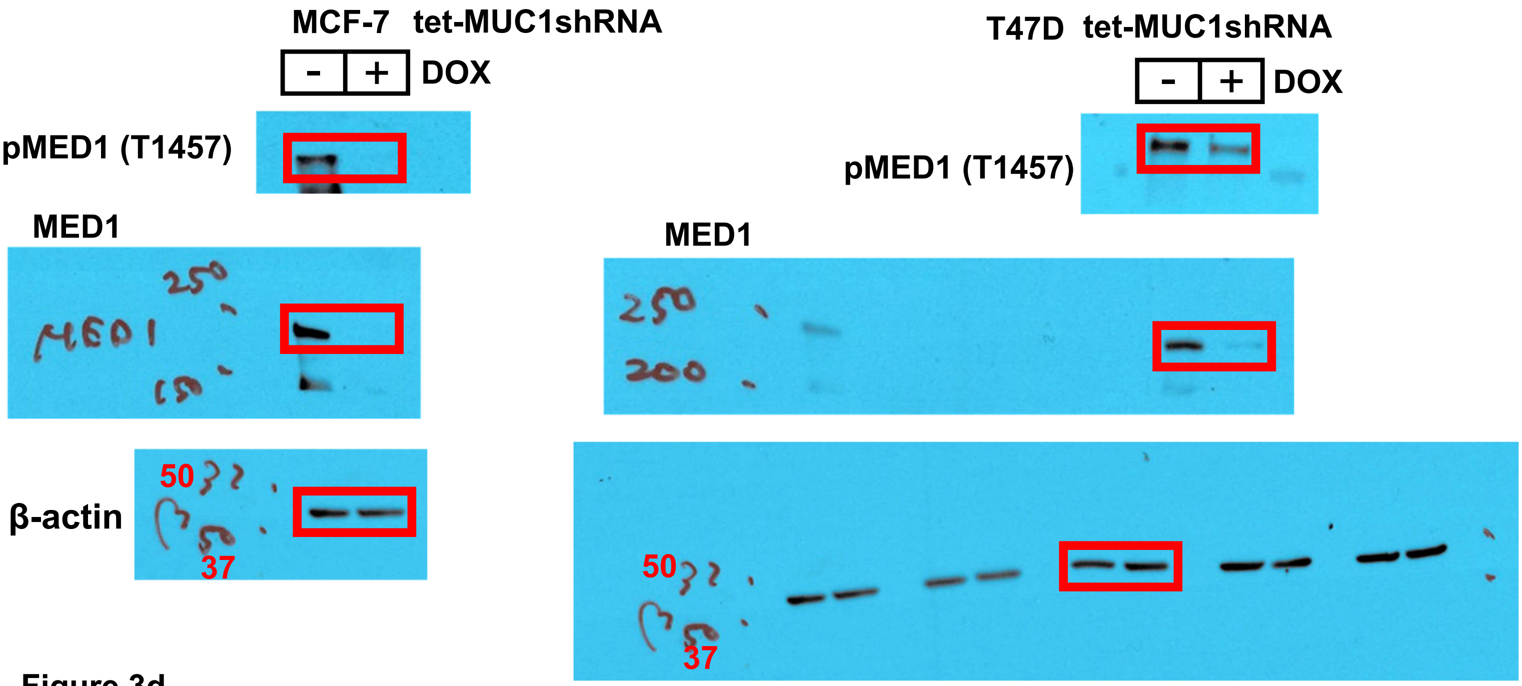

Figure 3d

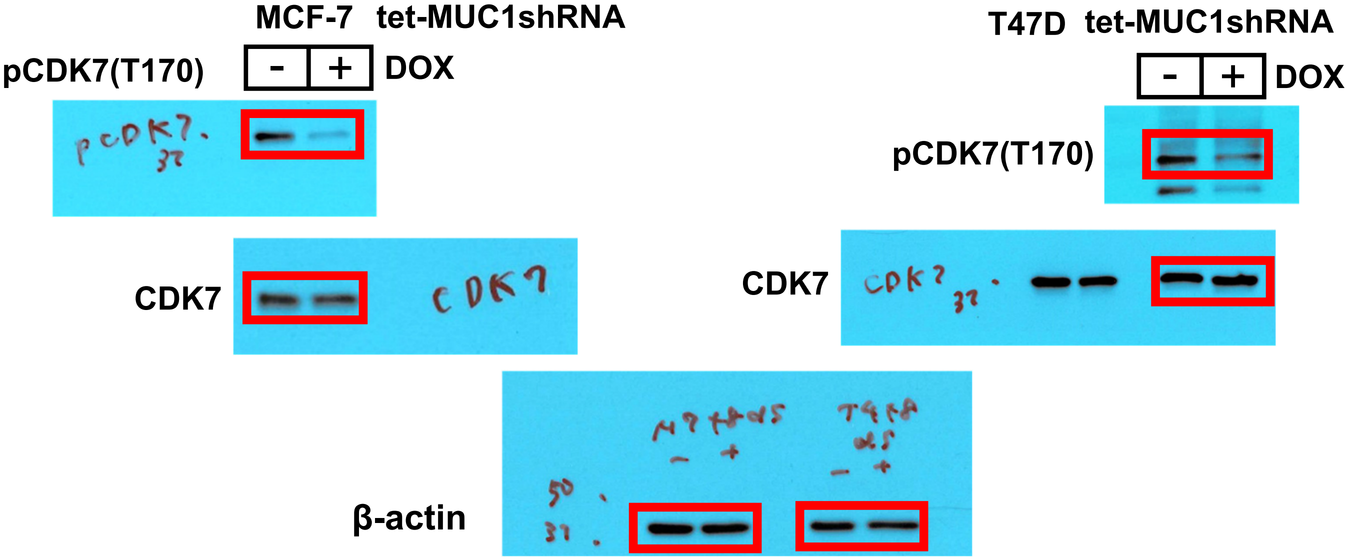

Figure 4a

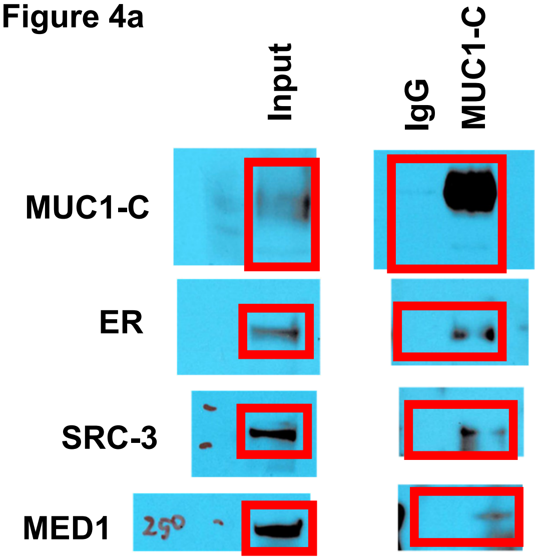

Figure 4b

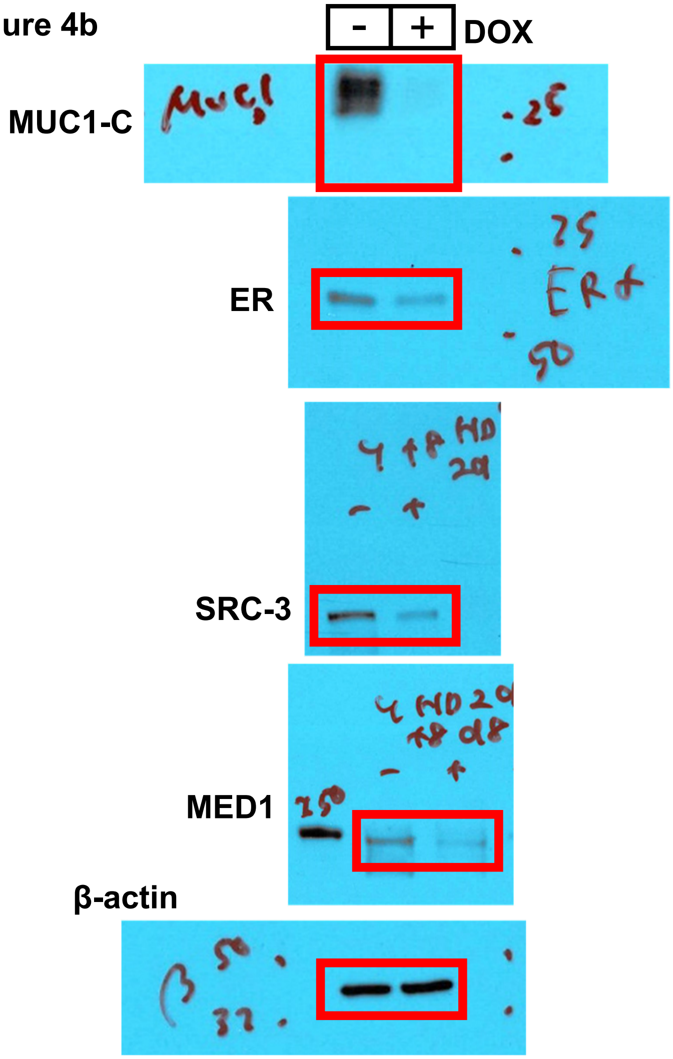

Figure 4c

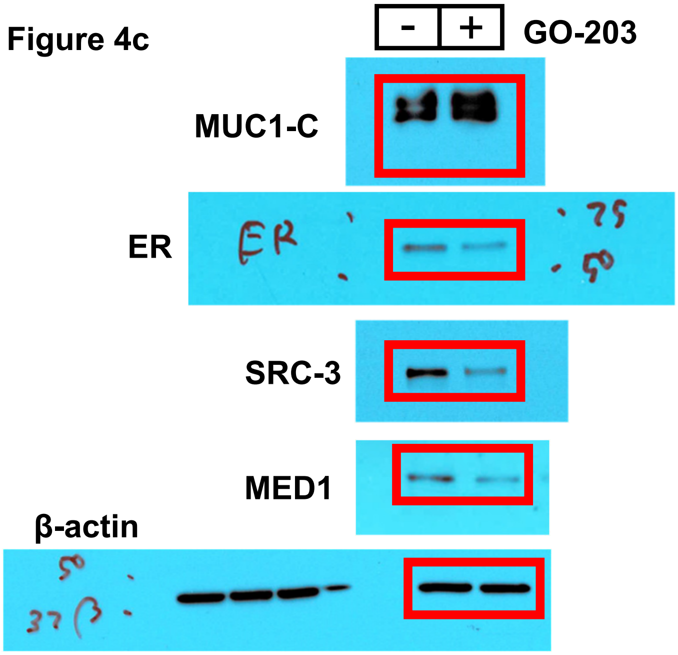

Figure 5a

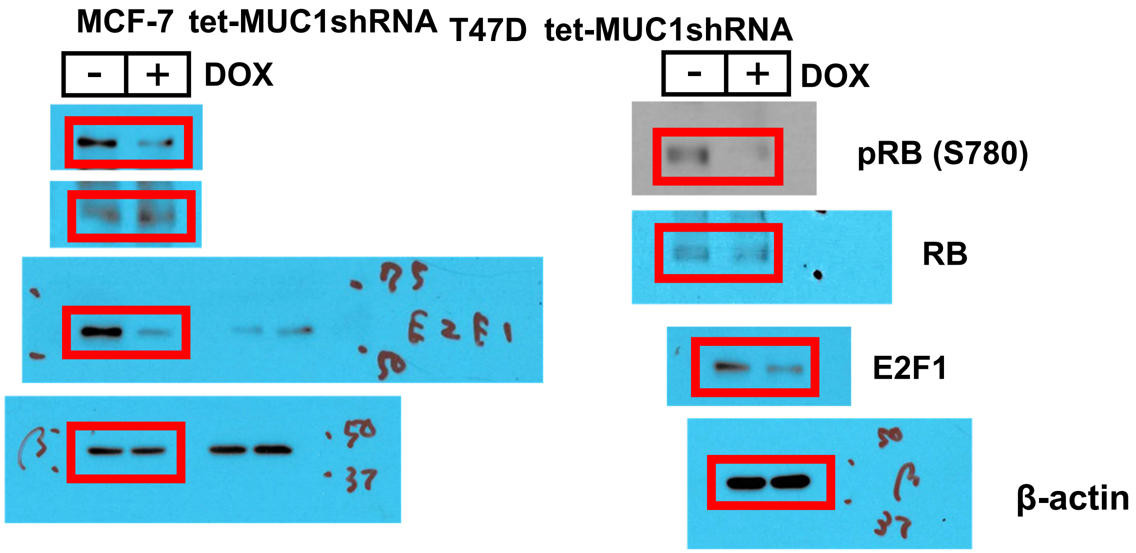

Figure 5d

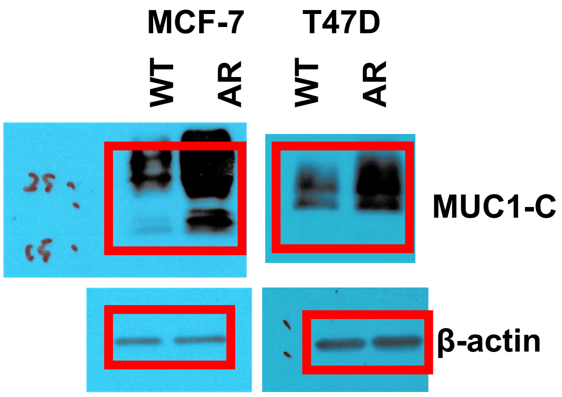

Figure 5e

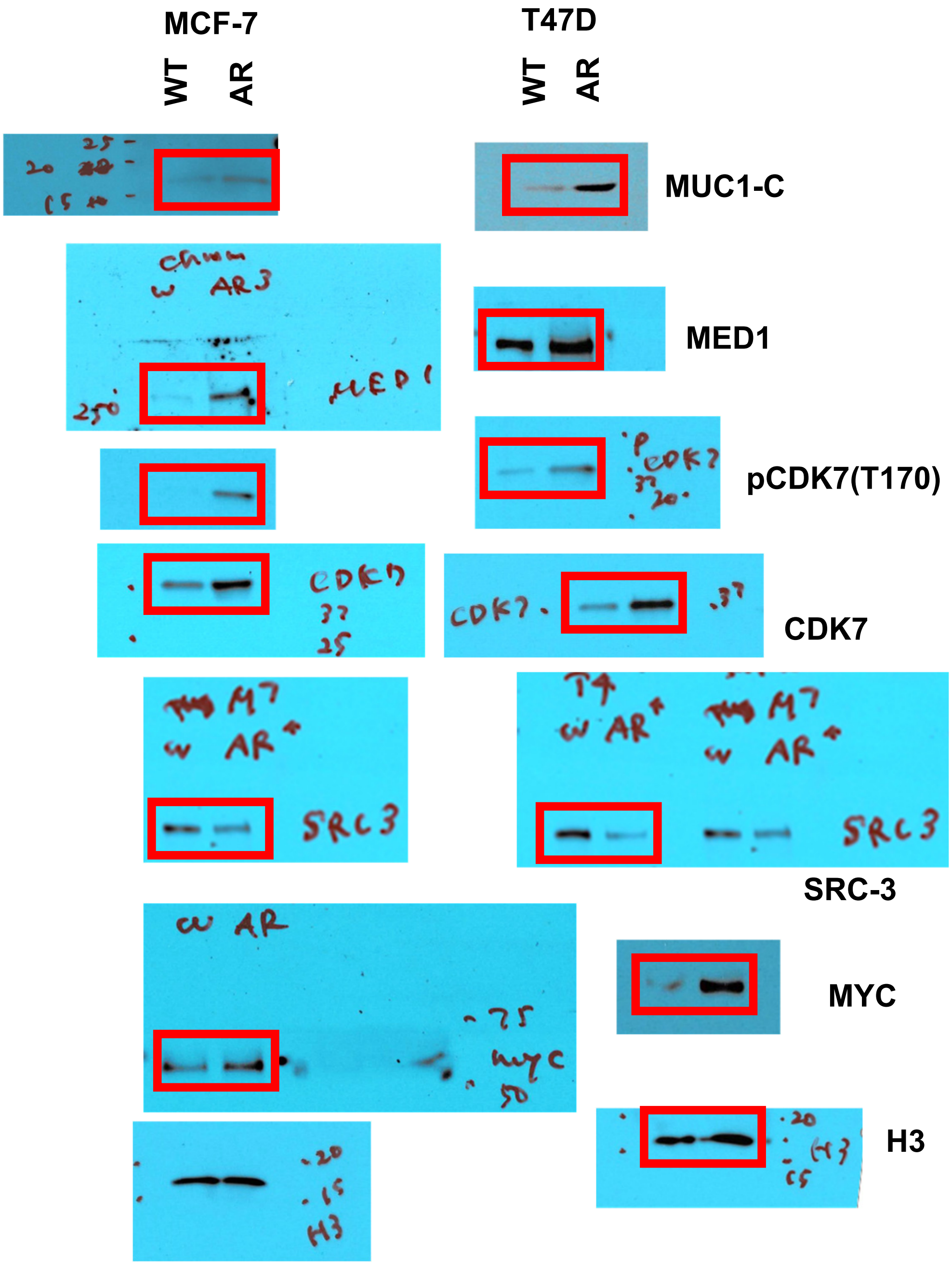

Figure 5f

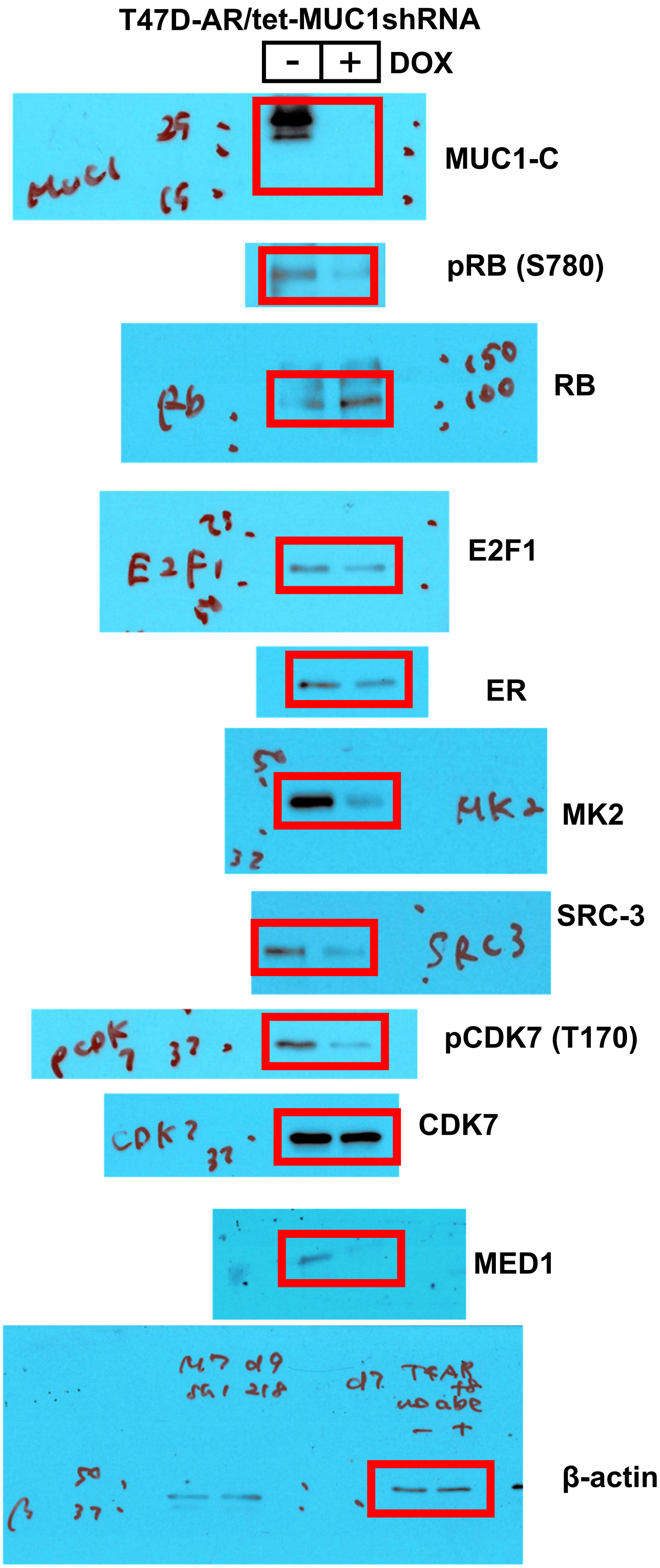

Figure 5g

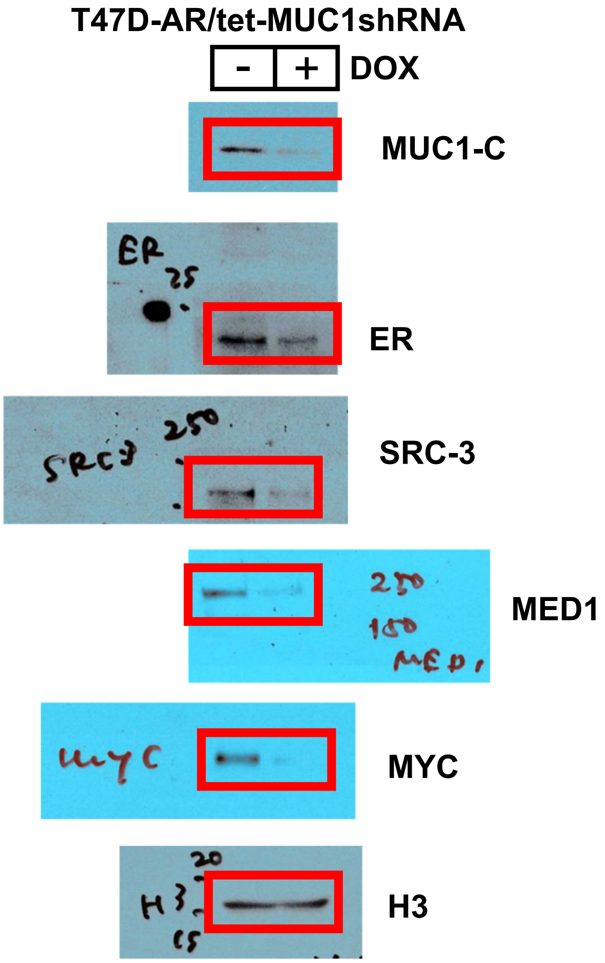

Supplement: Supplementary file 1 — Supplementary Figures 1–8, Supplementary Tables 1–3 [file 41523_2025_751_MOESM1_ESM.pdf]
